# Recent Advances in Self-Healing Hybrid Polyurethanes: From Molecular Design to Intelligent Functional Materials

**DOI:** 10.3390/ma19214522

**Published:** 2026-10-27

**Authors:** Justyna Gołąbek, Michał Strankowski

**Affiliations:** 1 Department of Polymer Technology, Faculty of Chemistry, Gdańsk University of Technology, 80-233 Gdańsk, Poland; micstran@pg.edu.pl; 2 Beckman Institute for Advanced Science and Technology, University of Illinois at Urbana-Champaign, Urbana, IL 61801, USA

**Keywords:** self-healing, hybrid polyurethanes, nanoparticles, MXenes and MOFs

## Abstract

Self-healing materials (SHMs) have attracted the attention of scientists as they represent a diverse type of novel functional materials in the field of smart polymers. They are capable of repairing physical damage, resulting in an extended service life of materials. Among various polymers, polyurethanes (PUs) have emerged as a highly versatile class of polymeric materials due to their intrinsic structural tunability, mechanical robustness, and compatibility with dynamic chemical motifs. However, it is still challenging to achieve excellent mechanical properties and excellent healing efficiency at the same time. To address this challenge, dynamic covalent bonds and dynamic non-covalent interactions have been incorporated into polyurethane networks with varying degrees of success. Consequently, hybrid strategies have emerged as a promising approach. Such hybrid systems can be achieved through the integration of dynamic covalent bonds and reversible non-covalent interactions within a single polymer network, enabling synergistic optimization of mechanical strength, adaptability, and healing efficiency. Alternatively, hybrid polyurethane composites can be developed through the incorporation of functional nanofillers and additives (including carbon-based materials, two-dimensional materials such as MXenes, where M represents a transition metal and X represents carbon or nitrogen, metal–organic frameworks (MOFs) and covalent organic frameworks (COFs)), which impart additional functionalities and broaden the application scope of polyurethane systems. This review summarizes recent progress in self-healing hybrid polyurethanes, highlighting molecular design strategies, structure–property relationships, and emerging approaches toward intelligent multifunctional materials.

## 1. Introduction

Plastics have revolutionized and significantly influenced our society and current lifestyle, allowing us to use a variety of polymer-based materials that have become a cheaper substitute to previously used metals, ceramics and glasses, wood-based composites, etc. However, the difficulty lies in managing their lifecycle, which often ends after just one cycle [1]. According to the Organization for Economic Co-operation and Development Global Plastics Outlook, the recycling rate for 2023 was only 9%, while the rest was either incinerated (19%), mismanaged (22%) or in the worst case landfilled (49%) [2]. According to the future estimations, recycling rate is expected to improve up to 17% by 2060 [3]. Therefore, plastic waste management is now one of the main global challenges which requires decisive action especially in the term of developing new technologies and coming up with new strategies [4]. There are a number of detailed studies and reviews focused on bio-based “eco-friendly” plastics [5,6,7,8,9] and mechanical and chemical recycling technologies [10,11,12,13,14]. Recycling undoubtedly plays a very important role in the polymer end-of-lifecycle in a circular economy; however, considering that only about 7.87 mln tons out of 87 mln tons of plastic produced in 2022 were recycled, doubts and questions arise about the effectiveness of such solutions [15].

A new class of material emerged as a solution to these challenges. Self-healing polymers are novel functional materials in which self-healing refers to the ability to repair damage in the form of microcracks, plastic yield or fracture and restore (completely or to some extent) original function autonomously (spontaneously). Damage is triggering the healing process autonomously or non-autonomously through external stimulus such as heat, light, mechanical force and chemical change [16]. In 2015, van der Zwaag [17] introduced the concept of three factors that have an impact on self-healing: localization, temporality and mobility. Localization refers to the position and size of the damage; temporality is the coefficient of the time relationship between the occurrence of damage and repair; and mobility refers to polymer chains motions which promote the reversibility of bonds. The fourth concept—mechanism—is related to the classification of self-healing, extrinsic and intrinsic [18,19].

**Figure 1:**
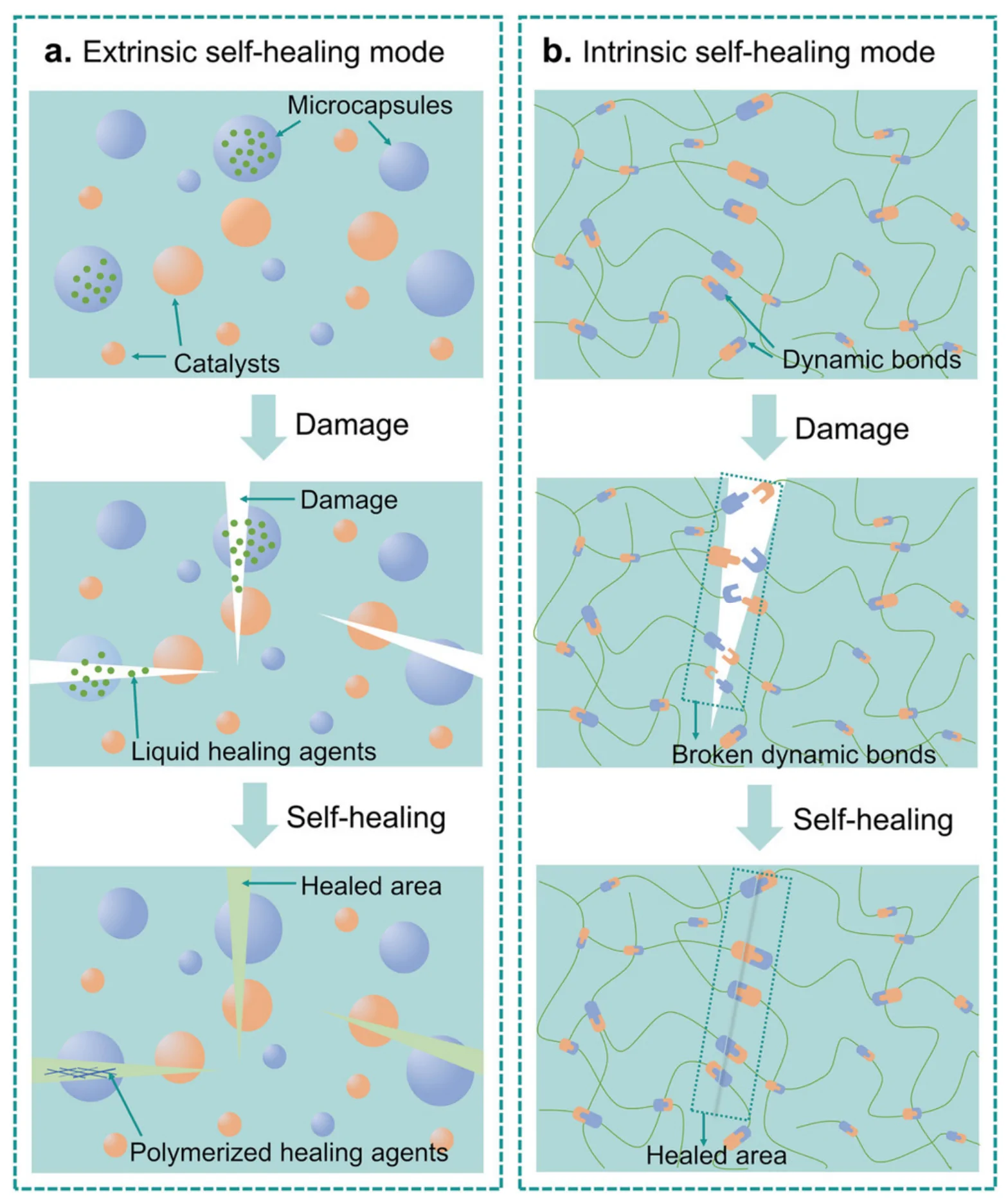
Extrinsic self-healing mode (**a**) and intrinsic self-healing mode (**b**). Reprinted with permission from [19]. Copyright 2021 Wiley-VCH GmbH.

The extrinsic self-healing method (Figure 1a) is based on the addition of external agents in the form of capsules or vascular networks filled with healing agents [19]. In the case of microcapsule embedment, when the damage occurs, the microcapsules break and release the healing agent with the catalyst, which flows toward the crack and spreads due to the surface tension between the two separated surfaces, and through polymerization it seals the damage. The catalyst interacts with precursors and forms a network that prevents the spread of the fracture and restores the mechanical properties. However, after the depletion of the local healing agent no more healing action can occur in this region [20]. In the case of microvascular systems, the healing agent is incorporated into a network composed of hollow fibers or a mesoporous structure incorporated into the composite material. This technique enables multiple local healing as the network may be refilled; however, the healing agent must relocate and mix by diffusion over considerable distances to reach the damaged zone [20,21]. The benefits of hollow-fiber-based self-healing include the vast amount of repairing agents and numerous activation methods. The limitations are that without the fracture of hollow fibers the discharge of the repairing agent is impossible and a high viscosity of repairing agent hinders fiber infiltration [20]. The extrinsic mechanism is used especially for thermosets like epoxy-based composites [22] and found application in coatings [23], instrument panels in automobiles [24] and sponges [25].

On the other hand, the intrinsic self-healing process (Figure 1b) is based on the properties of the material resulting from its composition and structure, where no external addition is required for the healing process to occur. Such a process is a very beneficial feature used especially for elastomers in new applications like nanogenerators [26,27], sensors [28,29], conductive materials [30,31], and smart coatings [32]. The healing mechanism relies on the fracture and recombination of dynamic reversible covalent bonds or non-covalent bonds triggered by external factors such as temperature, electromagnetic radiation, pH change, humidity or ionic strength changes [33]. Compared to the former mechanism, the intrinsic self-healing method may occur several times through inherent reversible covalent bonds or supramolecular interactions [34]. For this reason, intrinsic-based materials are more durable and reliable compared to extrinsic-based ones. Moreover, the repeatability and universality of intrinsic self-healing processes give them greater practical potential. Nevertheless, introducing either extrinsic or intrinsic mechanisms results in a trade-off with essential polymer properties such as mechanical strength [35].

In recent years, polyurethanes have been particularly popular among synthetic materials with self-healing properties. Polyurethanes, which are composed of two types of segments—soft (mainly polyether- or polyester-based) and hard (composed of diisocyanate and chain extender)—undergo microphase separation creating two domains. One of them acts as a physical crosslink and the second one acts as a matrix [36]. Polyurethanes are characterized by superior properties such as thermal stability, chemical and harsh environmental resistance, low production cost, a vast variety of components, good mechanical properties combined with high elasticity, ease of processing (as typical thermoplastics) and more. By carefully selecting the components and controlling the ration of hard to soft segments, the phase-separated structure can be tuned, which in turn allows the material properties and potential applications to be tailored to a certain extent [37]. However, their durability and end-of-life issues significantly affect the economic losses associated with premature aging of the material, but also increase the amount of pollution impacting air, land and water. Thus, a proper design aimed at improving these properties and extending the lifecycle of polymer materials is crucial [38].

To overcome the limitations associated especially with the trade-off between mechanical performance and self-healing efficiency, an increasingly important strategy involves incorporating functional nanomaterials into polyurethane matrices, such as carbon-based nanomaterials, MXenes, metal–organic frameworks (MOFs) and covalent organic frameworks (COFs). These additives can serve a complementary purpose: reinforcing the polymer network, improving interfacial interactions and barrier properties, or introducing additional functionalities. What is more, selected nanomaterials can also assist the activation of dynamic bonds, promoting polymer-chain mobility and the rearrangement or exchange of reversible interactions [39,40,41,42].

Carbon-based nanomaterials, including graphene derivatives and carbon nanotubes (CNTs), are promising additives for self-healing polymer composites due to their high mechanical strength, flexibility, electrical conductivity, and potential photothermal properties. They can enhance polymer reinforcement and assist self-healing through reversible interactions or external stimuli, such as heat, electricity, and light. However, challenges related to dispersion, interfacial compatibility, and processing remain critical for developing durable, multifunctional self-healing materials [39,40]. MXenes can be incorporated into polyurethane matrices to enhance electrical conductivity, photothermal conversion, and mechanical functionality while supporting light- or heat-triggered self-healing. Their surface functional groups promote interfacial interactions with PU chains, facilitating nanosheet dispersion and the formation of stable conductive networks. However, preventing MXene aggregation and ensuring strong interfacial compatibility remain essential for achieving durable, multifunctional PU composites [43]. Metal–organic frameworks can be incorporated into polyurethane matrices as multifunctional additives to improve mechanical strength and support self-healing. Their porous structures enable the loading of healing agents or catalysts, while their tunable chemical functionality can promote interactions with polymer chains and support reversible bonding. Depending on their design, MOFs can assist intrinsic or extrinsic healing and introduce additional functionalities, such as electrical conductivity and sensing. However, their effectiveness depends on dispersion and interfacial compatibility with the PU matrix [41]. Covalent organic frameworks are porous crystalline materials whose chemical structure and functionality can be tailored for polymer composites [44]. This is a relatively new development in the field of self-healing polyurethanes; however, the literature indicates that they can be incorporated as dispersed fillers to improve mechanical performance and introduce photothermal functionality [45].

There are several review papers on self-healing polyurethanes that concentrate on distinguishing between the two mechanisms—covalent and non-covalent—and describing their different interactions separately [46,47,48,49,50,51,52]. However, in our opinion, the literature regarding the utilization of hybrid systems comprising both interactions (molecular hybridization), with particular attention paid to the introduction of additives such as graphene derivatives, carbon nanotubes, MXenes, MOFs and COFs (nano-interfacial hybridization), is currently limited.

Therefore, the objective of this review is to comprehensively analyze and summarize recent advances in self-healing hybrid polyurethanes. First, we outline the fundamental interactions involved in intrinsic self-healing and discuss the characterization methods used for the quantitative evaluation of healing performance. We then compare dynamic covalent and non-covalent self-healing mechanisms, highlighting their synergistic effects when combined within a single material, as well as their ability to withstand repeated healing cycles, and discuss their reprocessing and recyclability and the trade-off between healing efficiency and mechanical performance. Subsequently, we examine two main strategies for designing hybrid materials: molecular hybridization and nano-interfacial hybridization. Particular attention is paid to the incorporation of carbon-based nanomaterials, including graphene derivatives and carbon nanotubes (CNTs), MXenes, as well as metal–organic frameworks (MOFs) and covalent organic frameworks (COFs). The emphasis is put on their roles in enhancing mechanical properties, self-healing and introducing additional functionalities. Finally, we identify the main challenges limiting their practical application and outline future research directions for the development of durable, multifunctional and sustainable hybrid systems.

## 2. Intrinsic Self-Healing Mechanisms

### 2.1. Characterization and Quantitative Evaluation of Self-Healing Performance

Self-healing refers to the ability of a material to recover properties or functions degraded by damage through the re-establishment of interaction or bonds across damaged interfaces. In polyurethanes, healing performance can be evaluated through visual crack closure, mechanical recovery, or the restoration of functional properties, depending on the intended application. However, the disappearance of a visible crack does not necessarily indicate complete recovery of the material’s mechanical integrity [21].

There are many different metrics and definitions that can be used to describe the extent of recovery. A commonly used quantitative parameter is the self-healing efficiency η, defined as the ratio of property recovery after healing to the property loss caused by damage [21]:

$$\eta =\frac{f_{healed}-f_{damaged}}{f_{virgin}-f_{damaged}};$$

where f is the analyzed property of the pristine, damaged, and healed material.

It is most often expressed as a percentage, in accordance with the formula [53]:

$$Healing\;efficiency\;\left(\%\right)=\frac{f_{healed}}{f_{damaged}}\times 100;$$

where f is the property value.

The property used for evaluation must be specified, for example as the recovery of tensile strength, elongation at break, fracture toughness, or barrier performance, and may differ considerably. Consequently, healing efficiencies obtained using different properties or experimental protocols should not be directly compared without considering the testing conditions. In this context, quantitative measurements should be complemented by structural characterization to distinguish apparent crack closure from the actual recovery of material properties [21].

A reliable assessment of self-healing performance requires more than the visual observation of crack closure. Although microscopy and other imaging techniques can provide useful evidence of changes in crack geometry and surface morphology, the apparent disappearance of a defect does not necessarily demonstrate the restoration of the original mechanical or functional properties. Accordingly, structural observations should be distinguished from quantitative measurements of property recovery [21,53,54,55,56]:

(i) Visual and morphological characterization [53,55]: Optical microscopy and scanning electron microscopy can be used to examine the dimensions and morphology of damage before and after the healing treatment. These techniques can help to establish whether a visible defect has narrowed or closed. However, morphological evidence alone is insufficient to demonstrate complete mechanical recovery.
(ii) Mechanical characterization [54,56]: Mechanical testing provides a direct means of assessing the extent to which a selected property has been restored after damage. Depending on the intended application and the nature of the material, relevant methods may include tensile testing, measurements of elongation at break, modulus determination, and fracture tests. The selected property and testing protocol should be reported explicitly, and the same measurement method should be used before damage and after healing whenever a recovery ratio is calculated.
(iii) Chemical and spectroscopic characterization [55,56]: Techniques such as Fourier-transform infrared spectroscopy, Raman spectroscopy and nuclear magnetic resonance spectroscopy may be used to investigate changes in chemical structure or to obtain evidence consistent with the proposed bonding mechanism. Their interpretation should be mechanism-specific: spectroscopic changes may support a chemical explanation for healing, but they do not independently establish the extent of mechanical recovery.
(iv) Thermal and dynamic characterization [54]: Differential scanning calorimetry, dynamic mechanical analysis, and stress-relaxation measurements can provide complementary information about thermal transitions, viscoelastic behavior, and network dynamics. These methods are particularly useful when the proposed healing mechanism depends on segmental mobility or dynamic network rearrangement. Nevertheless, the relevance of each technique depends on the specific polymer architecture.
(v) Repeated damage-healing tests [21,54,56]: Repeated cycle experiments are necessary to determine whether a material retains its healing capability and functional properties during repeated use. Such experiments should document the number of cycles, the damage protocol, the healing conditions, and the property measured after each cycle. Reporting only the best result or the first healing cycle does not establish long-term durability.

Taken together, these characterization approaches provide complementary forms of evidence. A rigorous evaluation should combine direct measurements of property recovery with appropriate structural or chemical characterization, while recognizing that no single technique is sufficient for every self-healing mechanism or application [57].

### 2.2. Intrinsic Self-Healing

The intrinsic self-healing mechanism operates through repeated cycles of dynamic bond dissociation and reformation, enabling autonomous recovery of the material’s structure and restoration of its original functional properties following damage. In the context of self-healing polyurethanes based on the intrinsic mechanism, there are two types of bonds—dynamic reversible covalent bonds or non-covalent bonds [58]. Dynamic covalent bonds are based on the mechanisms of ditelluride bonds [59,60], diselenide bonds [61,62], disulfide bonds [63,64], imine bonds [65,66], boroxine bonds [67,68], alkoxyamine bonds [69,70], and Diels–Alder bonds [71,72]. Dynamic non-covalent bonds are listed as multiple hydrogen bonds [73,74], metal–ligand coordination bonds [75,76], π-π stacking [77], ionic bonds [78] and host–guest interactions [79].

#### 2.2.1. Dynamic Covalent Bonds

**Figure 2:**
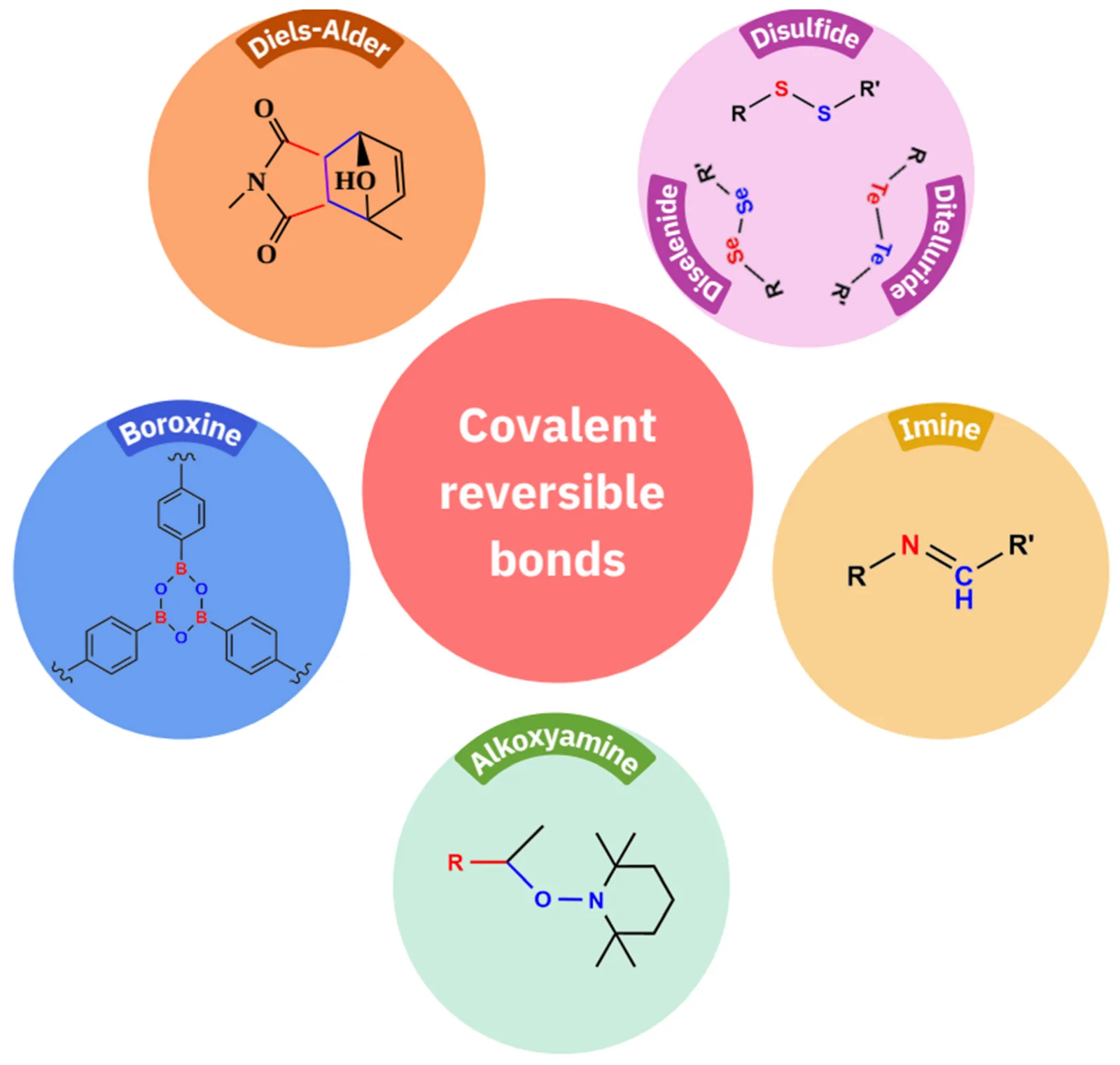
Covalent reversible bonds. The different colors indicate the type of reversible covalent bonds: pink represents disulfide, ditelluride, and diselenide bonds; yellow represents imine bonds; green represents alkoxyamine bonds; blue represents boroxine bonds; and orange represents Diels-Alder bonds.

Introducing reversible covalent bonds with relatively high bond energy into polyurethane molecular chains imparts the material with a self-healing ability and mechanical robustness. There are five groups of covalent-based interactions introduced into polyurethane systems, as shown in Figure 2 [80].

##### Ditelluride, Diselenide, and Disulfide Bonds

**Figure 3:**
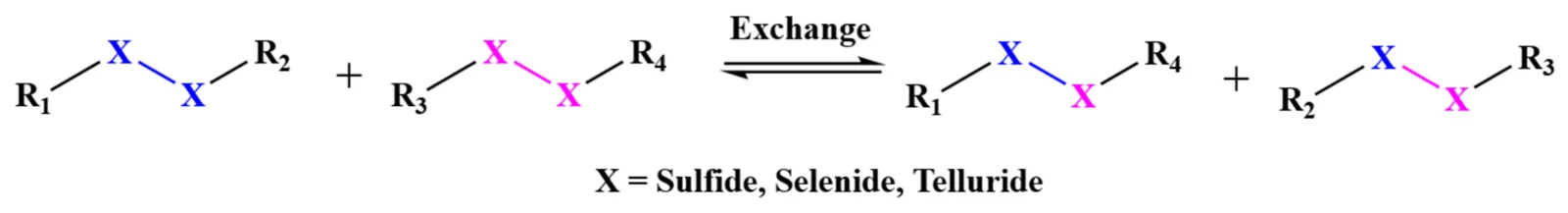
Disulfide/diselenide/ditelluride bond exchange. Based on [52].

Ditelluride bonds (126 kJ/mol), diselenide bonds (172 kJ/mol) and disulfide bonds (240 kJ/mol) are based on a simple exchange reaction of ditelluride, diselenide and disulfide radicals, simultaneously (Figure 3). The ditelluride bonds exhibit more sensitive stimulus responsiveness compared to the other two bonds because of the larger size of the atomic radius, thus exhibiting stronger dynamic characteristics [81].

Among dynamic covalent linkages, ditelluride (Te-Te) bonds remain comparatively unexplored. Compared with diselenide and disulfide analogues, ditelluride bonds have a significantly lower bond dissociation energy, enabling metathesis under comparatively mild conditions [82]. Visible light irradiation induces metathesis of ditellurides via formation of stable tellanyl radicals, which can recombine even in the absence of light, highlighting their potential for photoresponsive self-healing polymers, hydrogels, and sensors [81]. Zeng et al. [60] designed multifunctional waterborne polyurethane DTe-WPU films (polytetramethylene ether glycol PTMEG, dimethylolpropionic acid DMPA, isophorone diisocyanate IPDI, and 1,4-butanediol BDO) by introducing ditelluride bonds into the main chain, exhibiting rapid self-healing under visible light at room temperature, with healing efficiency of 85.4% stress recovery and 91.99% strain recovery within 30 min, maintaining strong tensile strength of 17.5 MPa. The film was processed into the form of a leather coating, possessing good antibacterial activity against Escherichia coli and Staphylococcus aureus (90.2% and 92.5%, respectively).

Diselenide bonds (Se-Se) undergo metathesis under mild stimuli such as heating (~70 °C) or visible light (>600 nm), enabling remote self-healing (e.g., laser irradiation) [83,84]. Owing to their lower bond dissociation energy, Se-Se bonds exhibit a more dynamic exchange reaction [52]. A key limitation remains the shallow light penetration depth, which restricts practical use mainly to coatings and thin films. Liu et al. [61] developed bio-based polyurethane networks incorporating castor oil, hexamethylene diisocyanate (IPDI), tolylene-2,4-diisocyanate (TDI), hexamethylene diisocyanate (HDI) and synthesized di(1-hydroxyethylene) diselenide serving as a chain extender. The Se-Se bonds enabled reversible metathesis under mild heating (70 °C), allowing full damage recovery within 2 h. These materials showed triple shape memory properties, with a shape fixation ratio exceeding 99%, recovery ratio above 60%, notable tensile strength (6.5 MPa), and high elongation at break (up to 468%). Dynamic Se-Se exchange also imparted recyclability and reprocessability, demonstrating the potential of diselenide chemistry in high-performance bio-based PUs.

Based on the literature research [85,86,87,88], disulfide bonds (S-S) are one of the most studied and developed examples of covalent bonds. They can be divided into aliphatic or aromatic bonds. Aliphatic S-S bonds undergo metathesis reaction under a UV light source [89], whereas aromatic S-S bonds do not require the use of any external agents [90]. Xian et al. [91] prepared castor oil-based polyurethanes (D-CPU) using 2,2’-Diaminodiphenyl disulfide (DTDA) as a chain extender. As the DTDA content increased [0.6–1.1-NH_2_(DTDA) molar ratio)], the tensile strength of the PUs progressively increased (10.53 MPa to 28.78 MPa, respectively) while elongation at break decreased (770.19% to 208.99%, respectively). Healing efficiency showed a maximum at intermediate S-S content, because higher density promoted interfacial exchange, while increased hard segment content and hydrogen bonding restricted chain mobility. The optimized sample achieved a healing efficiency above 99% (healing at 100 °C for 4 h). Zhang et al. [92] fabricated a self-healing perovskite film by introducing IPDI and 2,2’-dithiodiethanol (HEDS) monomers into perovskite precursor solutions from PU additives. Moreover, a flexible device based on a perovskite solar cell with a structure composed of polyimide (PI)/ITO/SnO_2_/MAPbI_3_/carbon was assembled. The device maintained 83.1% of its initial power conversion efficiency (PCE) after 2000 bending cycles and recovered to 94.6% after overnight storage at room temperature.

##### Imine Bonds

**Figure 4:**
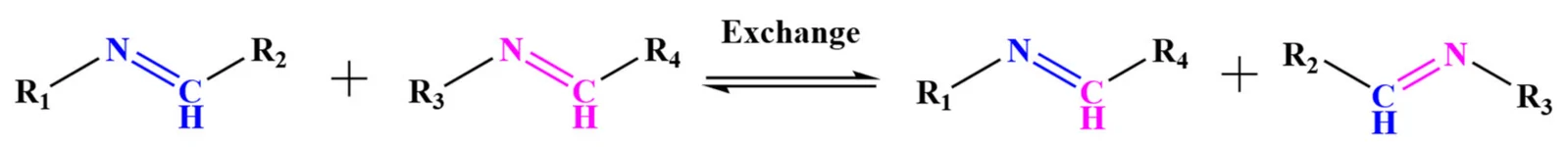
Imine bond exchange. Based on [52].

The reversible imine bond known as a Schiff base with a bond dissociation energy of 147 kJ/mol is a structure obtained by the condensation of aldehydes or ketones with primary amines, hydrazine or hydroxyl-mine under mild conditions, often acid-catalyzed conditions (Figure 4) [93,94]. In self-healing materials, they undergo reversible hydrolysis/condensation, transamination, and amine methathesis [52]. However, the rapid dynamics of the reversible imine bond negatively impact mechanical properties. Although increasing the crosslinking density of polyimine vitrimers improves strength, it also raises the glass transition temperature and thus reduces self-healing properties. Thus, polyurethanes with microphase separation represents a suitable candidate for strong C=N double bond to develop an elastomer with satisfactory self-healing and mechanical performance [94].

Recently, a series of light-cured bio-based polyurethane coatings incorporating imine bonds were synthesized using T-NH monomers from p-phthalaldehyde and poly(propylene glycol) bis(2-aminopropyl ether). PU formulations were prepared under UV curing from castor oil, epoxy soybean oil, glycerol monostearate, and sorbitan monooleate. All fabricated coatings possessed some self-healing ability (up to 60% efficiency) at room temperature, with performance enhanced to more than 75% self-healing efficiency by heating to 50 °C. The best-performing coating, derived from glycerol monostearate, achieved a self-healing efficiency of 90% and could be healed through four cycles [95]. Another group synthesized a bio-based waterborne PU films by incorporating a vanillin-derived diol chain extender with imine bonds using a one-step condensation of vanillin and ethylenediamine (VAN-OH), DMPA, polytetrahydrofuran (PTHF) and IPDI. The dynamic linkages enabled surface scratches to fully repair within 30 min at 80 °C through reversible imine bond exchange. Moreover, these films achieved a tensile strength of 12.8 MPa, compared to 4.3 MPa control, and higher thermal stability, which gives them potential in protective coatings, high-performance adhesives, and flexible elastomers [96].

##### Boroxine Bonds

**Figure 5:**
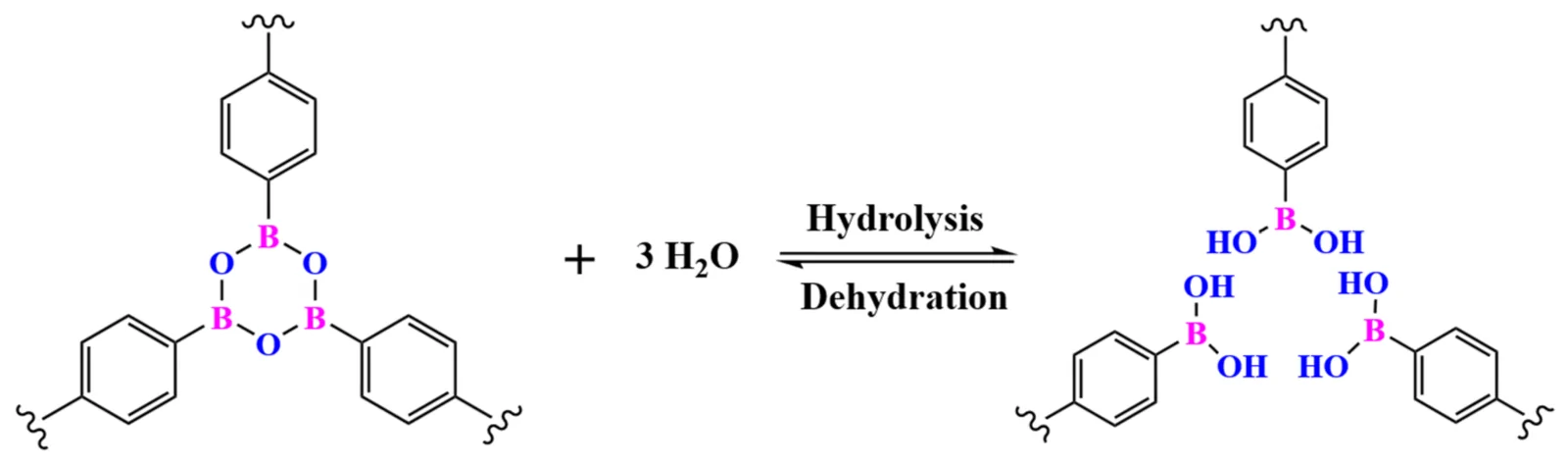
Boroxine bond hydrolysis/dehydration. Based on [52].

Boroxine bonds are obtained by dehydration and condensation of boronic acid (e.g., phenylboric acid) with a characteristic tripodal molecular architecture (Figure 5), having the ability to increase the crosslinking density of the doped polymer. The boroxine and boronic acid can be converted into each other through hydrolyzation or dehydration; however, the balance between these two is affected by factors such as temperature and the addition or removal of Lewis base or water, which influence the self-healing performance [52,68].

By far, compared to other covalent bonds, the most interesting aspect of the boroxine bond is how the self-healing process can be promoted—through water and temperature. It has been used by Longfang et al., who incorporated boroxine into a PU system (polypropylene glycol PPG, IPDI, BDO) to impart self-healing behavior. The boroxine-based polyurethane (BPU) films were cut, exposed to water for 1 min, and healed after 20 min at 60 °C (92% healing efficiency). Healing occurs through water-assisted hydrolysis and thermally reversible boroxine bond dissociation and recombination, which enhances chain mobility and restores the original material structure [68]. In another study, a boroxine crosslinked polyurethane (PBU) coating was developed by integrating phenylboronic acid groups into polyurethane chains (IPDI, ester-modified polytetramethylene ether glycol EPTMEG, and N,N-bis(2-hydroxyethyl) methylamine MDEA) through dehydration condensation. The BPU exhibited a healing efficiency of 94% (50 min at room temperature), a recycling efficiency of 93% and strong adhesion to cotton fabric (1.42 MPa). Those fabrics exhibited reversible photoisomerism and excellent UV protection function (UPF = 85.8), highlighting potential for multifunctional textiles [97].

##### Alkoxyamine Bonds

**Figure 6:**
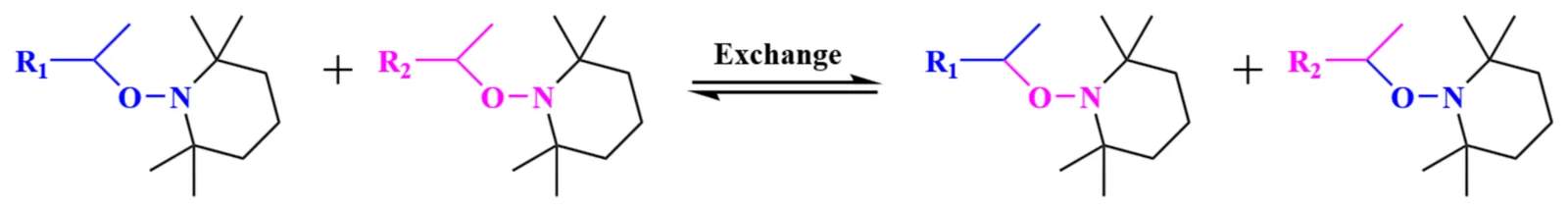
Alkoxyamine bond exchange. Based on [52].

Alkoxyamines (C-ON) are reversible structures derived from nitroxide radicals (e.g., TEMPO, TIPNO, SG1 and DPAIO) and carbon-centered radicals. These two types of groups can recombine together, undergo a dynamic exchange with the addition of other nitroxide radicals or initiate polymerization after thermal dissociation of a molecular bond by monomer addition [69]. The dissociation energy is at approximately 140 kJ/mol and carbon and oxygen radicals recombine above 60 °C; therefore, self-healing can be achieved through the reversible process of breaking and recombining C-ON bonds at relatively high temperature [98,99]. The reaction scheme is shown in Figure 6.

One of the first examples of incorporating polyurethane with an alkoxyamine bond was introduced by Yuan et al. [100] Alkoxyamine moieties with hydroxyl groups were integrated with polyurethane prepared by reaction of trifunctional HDI with diol and PEG with different molecular weights. A PU elastomer was synthesized, where dynamic reversible equilibrium between division or recombination of C-ON bonds allowed multiple reversible crosslinking of molecular chains and thus repeated healing at a temperature of 80 °C. Self-healing efficiency during tensile stress–strain evaluation was 70.7%, 57.9% and 42.6% after three healing cycles. However, the main reason for the extremely small number of reports on self-healing PUs with alkoxyamine bonds is their limited stability under the influence of oxygen, which hinders rebonding and reprocessing, restricting application possibilities [69,70].

##### Diels–Alder Bonds

**Figure 7:**
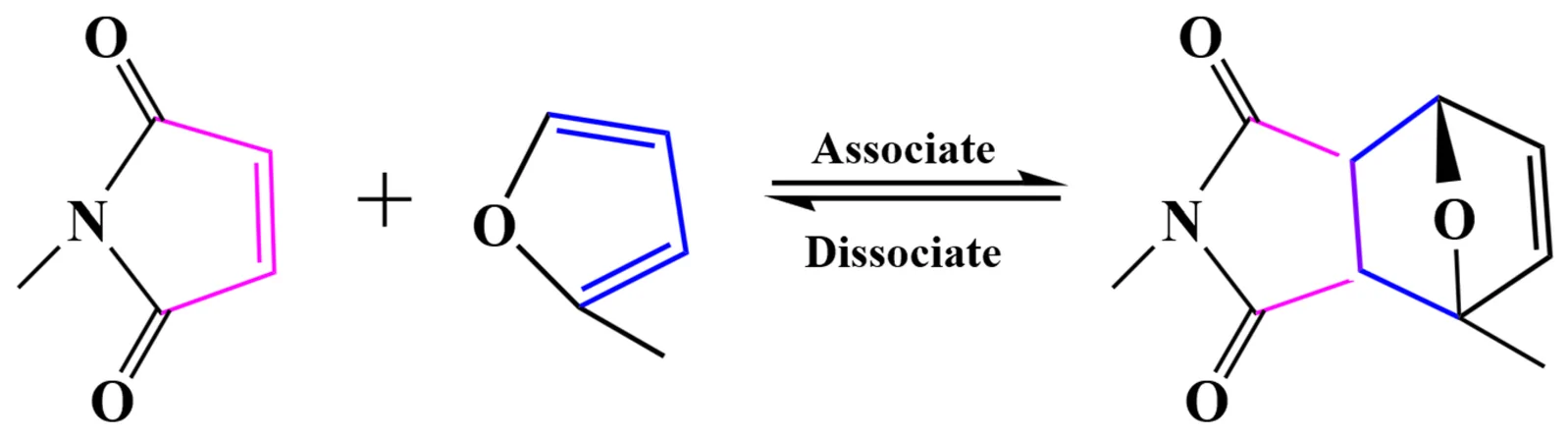
Diels–Alder associate/dissociate. Based on [52].

Thermally reversible Diels–Alder (D-A) cycloaddition reaction is the reaction that occurs between a conjugated diene (4π electron) and a dienophile (2π electron), in which a substituted cyclohexene derivative is formed (Figure 7) [52,101]. The D-A bond energy requires a high-temperature reaction for self-healing to occur, although no additional catalyst or coupling reagent is necessary [102]. The design for self-healing PU is based on either introducing D-A moieties into a PU backbone (by reaction between a diol adduct with polyol and di- or poly-isocyanate) or by chain-addition or pendant-addition of moieties during the first step of obtaining the polymer (maleimide/furfuryl alcohol, amine and thiol, or maleimide/furan isocyanate), followed by the addition of a thermoresponsive crosslinker in linear/crosslinked polyurethane [33,72].

A reaction between furfuryl alcohol (FA) and bismaleimide (BMI) named DAPU has been used by Xi and Wang [103] to obtain self-healing PU adhesives. The formation of Diels–Alder covalent bonds increased its crosslinking density and the rigidity of the material, highly improving the mechanical strength (~90 MPa). Its self-healing abilities were achieved by synergistic work via association/dissociation of hydrogen bonds and D-A bonds at a temperature of 130 °C. The synergistic effect of hydrogen bonds and Diels–Alder bonds was also adopted by Du et al. [104], where a series of PU-based electrolytes was prepared. Prepolymer was synthesized through a one-step synthesis between furan-2,5-diyldimethanol and HDI, then a D-A reaction was completed by introducing methoxy polyethylene glycol maleimide and N,N’-(4,4’-diphenylmethane) bismaleimide, and finally polyurethane polymer electrolyte was obtained by mixing prepolymer containing D-A bonds with lithium bis (trifluoro-methane sulfonyl) imide. The self-healing abilities were evident even after 10 s of scratching the sample at room temperature, where hydrogen bonds are responsible for repair between the interfaces, while D-A bonds restore the mechanical properties.

#### 2.2.2. Non-Covalent Bonds

**Figure 8:**
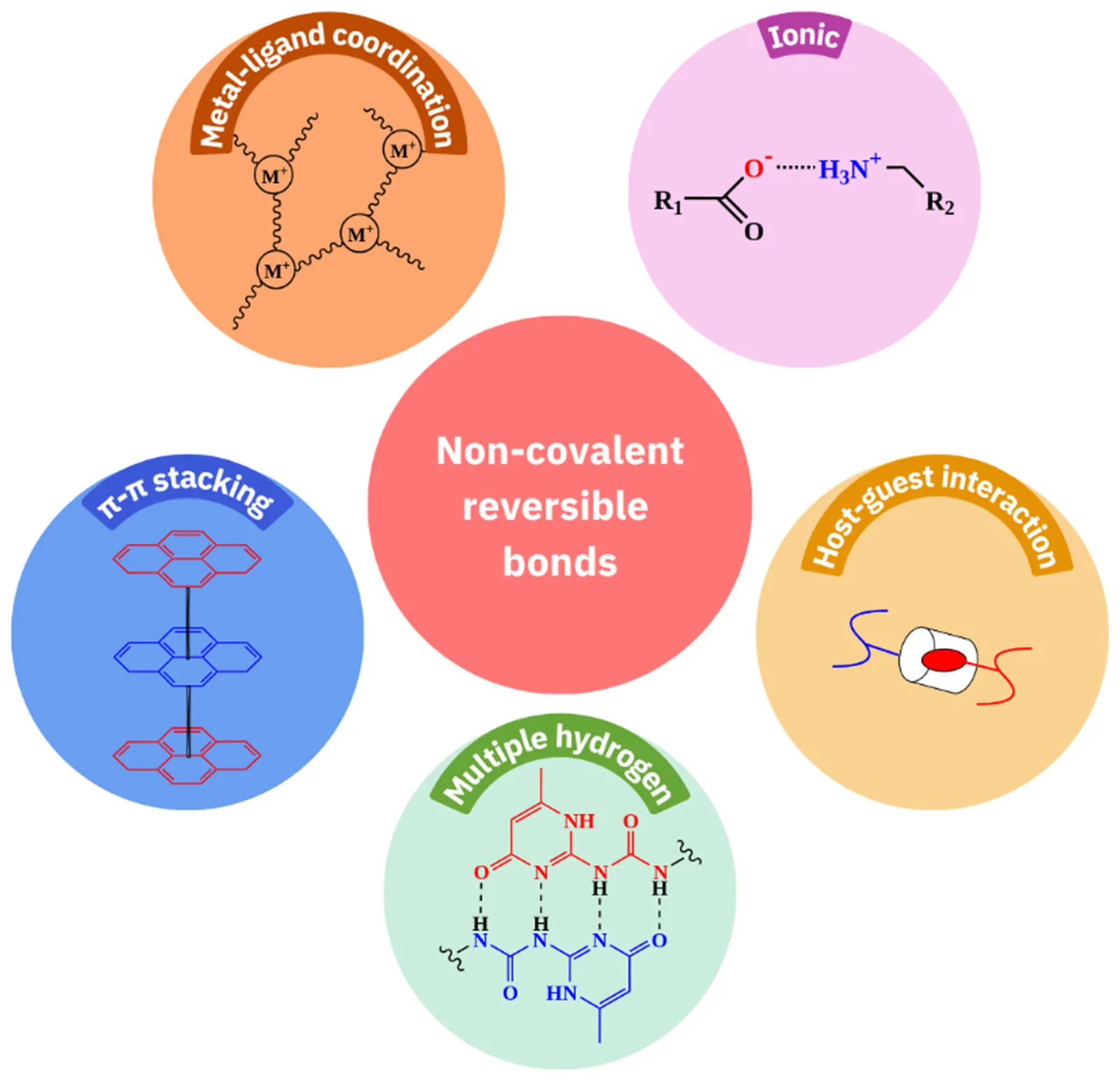
Non-covalent reversible bonds. The different colors indicate the type of non-covalent bonds: pink represents ionic bonds; yellow represents host-guest interactions; green represents multiple hydrogen bonds; blue represents π-π stacking; and orange represents metal-ligand coordination bonds.

Dynamic non-covalent interactions (Figure 8), including multiple hydrogen bonding, metal–ligand coordination bonds, host–guest interactions, ionic bonds and π-π stacking, are characterized by relatively low binding energies that enable reversible association and dissociation in response to external stimuli. These stimuli can be temperature changes, light, pH, or mechanical pressure, enabling repeated healing cycles [105]. Although the individual interactions are relatively weak, their collective and reversible nature provides a balance between mechanical integrity and network mobility required for efficient damage recovery. Introducing those interactions into polymeric materials supports their reprocessability, recyclability, and healing performance, making them attractive for the design of adaptive and multifunctional materials [105,106].

##### Multiple Hydrogen Bonds

**Figure 9:**
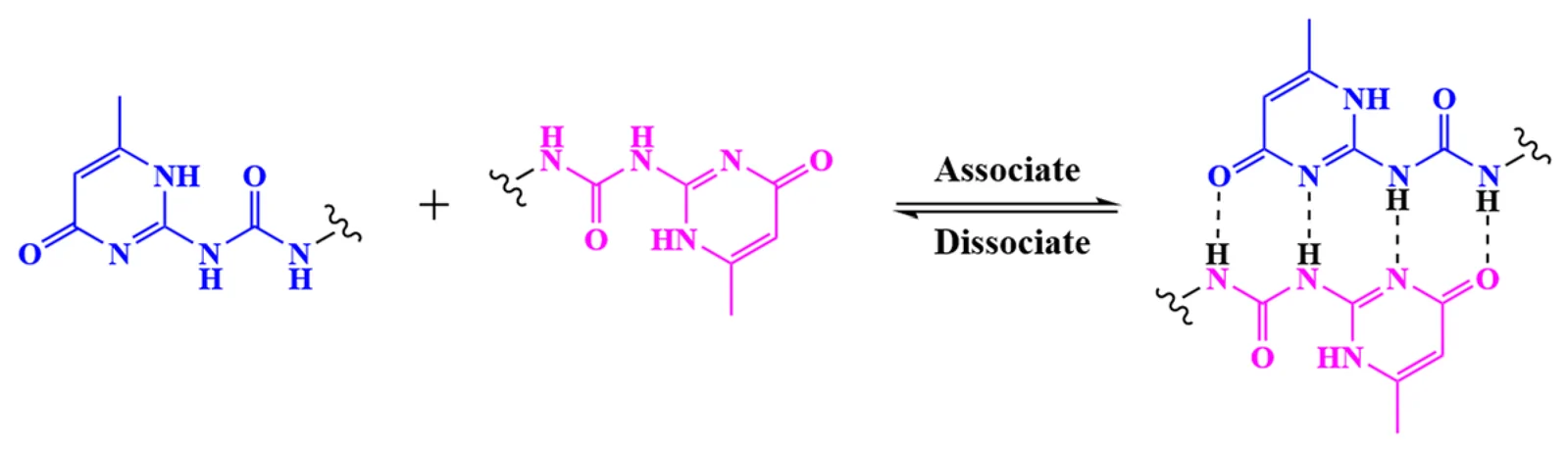
Multiple hydrogen bonds associate/dissociate. Based on [52].

Among non-covalent bonds, hydrogen bonds are by far the most commonly used types of supramolecular bonds for self-healing polyurethanes. The strength of the hydrogen bond is relatively weak (4 kJ/mol to 40 kJ/mol) [107] and depends on the nature of the proton donor (X-H) or proton acceptor (Y), where hydrogen bonds are expressed as X–…Y [108]. In polyurethanes, hydrogen bonds form between polymer chains through self-assembly of N-H (proton donors) and C=O (proton acceptors) in carbamate or urea groups. The reaction scheme is presented in Figure 9. Single hydrogen bonds yield low-strength materials with weak self-healing. However, multiple hydrogen bonds with strong interactions provide dense crosslinking within polymer networks and rapidly reform at fracture sites. However, those stronger physical crosslinks promote a more ordered structure, which can limit the chain, reducing healing efficiency [109,110].

In polyurethanes, hydrogen bonds are typically formed by carbamate, ureido-pyrimidinonone (UPy), and amide groups. Alavijeh et al. [111] reported a supramolecular shape memory-assisted self-healing PU coating with an isocyanate functionalized ureido-pyrimidinone (UPy-NCO) unit that introduced strong quadruple hydrogen bonding into the polymer networks. Fourier-transform infrared spectroscopy (FT-IR) confirmed the formation of non-covalent interactions, and increasing UPy-NCO lowered the glass transition temperature (T_g_), enhancing flexibility. A formulation with 45 wt% UPy-NCO exhibited pronounced shape recovery and macroscale crack healing efficiency up to 94%. Incorporation of PEG-400 as an additional polyol further reduced T_g_ and improved room-temperature flexibility, resulting in a durable, flexible coating with balanced mechanical resilience and rapid self-healing. Another group [112] published an article about a self-healing waterborne polyurethane elastomer (WTA) designed by 2-deoxythymidine (2’-dT) and isophorone diamine (IPDA), to introduce sextuple H-bonds between 2’-dT and urethane units and quadruple H-bonds among urethane group themselves. These hierarchical interactions improved the mechanical strength (from ~2.5 MPa for a WTA-0 to 15.3 MPa for WTA-3 containing 2’-dT and IPDA), toughness (13.94 to 100.75 MJ/m^3^) and healing efficiency (83.6%, 100 °C for 4 h). When compounded with multi-walled carbon nanotubes (MWCNT), a conductive device was fabricated that was able to light a small bulb, even after the sample had been cut and healed, illustrating potential for flexible electronic device applications.

##### Metal–Ligand Coordination Bonds

**Figure 10:**
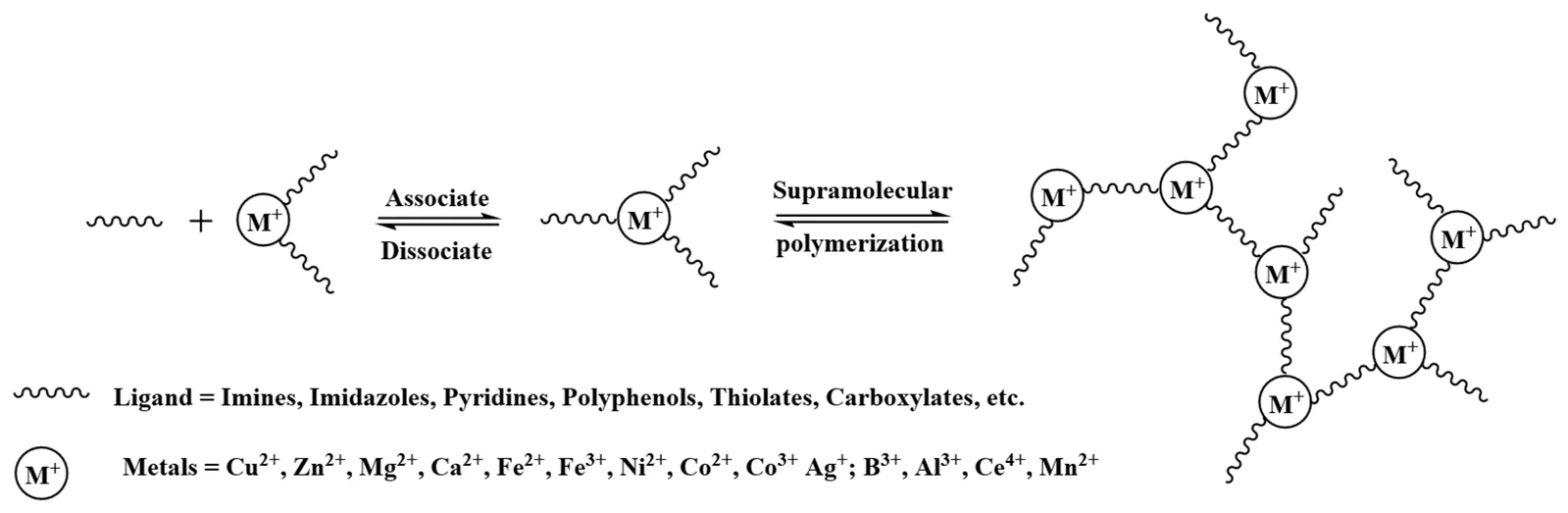
Metal–ligand coordination bonds. Based on [113].

Metal–ligand coordination bonds are formed from interactions between positively charged metal ions (Fe^3+^, Zn^2+^, and Cu^2+^) and partially negative ligand groups (catechol carboxylic acid, pyridine, and imidazole), which create coordination complexes (Figure 10), with moderate bond energy (50–200 kJ/mol) [46,113]. Coordination bonds strengthen intermolecular interactions and act as sacrificial bonds by increasing crosslink density and energy dissipation during stretching. Under external force, they dissociate and rapidly recombine, enhancing mechanical strength [46]. Such metal–ligand elastomers are highly stretchable, making them promising for soft robotics applications [114].

Li et al. [115] recently reported a polyurethane-based solid polymer electrolyte, in which the 2,6-diaminopyridine (DAP) ligand was used as a chain extender in reaction with PTMEG and IPDI, and subsequently crosslinked through dynamic coordination with Zr^4+^, Cu^2+^, or Zn^2+^, forming metal–ligand reversible coordinated PU. Self-healing arises from the dynamic dissociation and reformation of M-N_amide_ and M-O_amide_ coordination bonds; after damage, chain interdiffusion across the interface followed by recoordination enables recovery of ~90–95% of mechanical strength while maintaining interfacial integrity with a Li metal battery. The materials are intended for solid-state lithium metal batteries. In another work, a pyridine-functionalized polyurethane-urea elastomer incorporating Al^3+^ with acetylacetonates and phosphorus small molecules has been developed and coated with polypyrrole to fabricate a stretchable, self-healing and conductive composite for an electronic skin strain sensor, demonstrating self-healing ability, a stable sensor signal upon 100 alternating stretching/releasing cycles at 50% strain and high sensitivity to bending/unbending motions of the wrist and finger [116].

##### Host–Guest Interactions

Host–guest interactions describe the selective binding between two structurally distinct but dimensionally complementary molecules, where one of them is a host molecule such as cyclodextrins, cucurbiturils, or calixarenes, while the second one is called a guest and is a matching pair engaging in various non-covalent interactions, mainly hydrogen bonding, electrostatic forces, van der Waals interactions and hydrophobic interactions [117].

Miyagawa and Shibata [118] reported a supramolecular self-healing thermoset PU constructed through reversible host–guest interactions between α-cyclodextrin (host) and monomethyl ether (MPEG; guest) moieties. The host is covalently incorporated into the polymer network, while the guest can reversibly thread into the host cavity to form inclusion complexes acting as dynamic physical crosslinks. Mechanically, the cured films exhibited very low tensile strength and stress (less than 1 MPa) and healing efficiency, reaching only 38% (room temperature) and 62% (80 °C) after 24 h. Such results are still insufficient to be implemented in any application (e.g., protective coatings proposed by the authors). However, there are some promising publications about hybrid systems incorporating host–guest interactions which hold significant potential for applications in protective coatings and flexible sensors [119,120].

##### *π-π* Stacking

**Figure 11:**
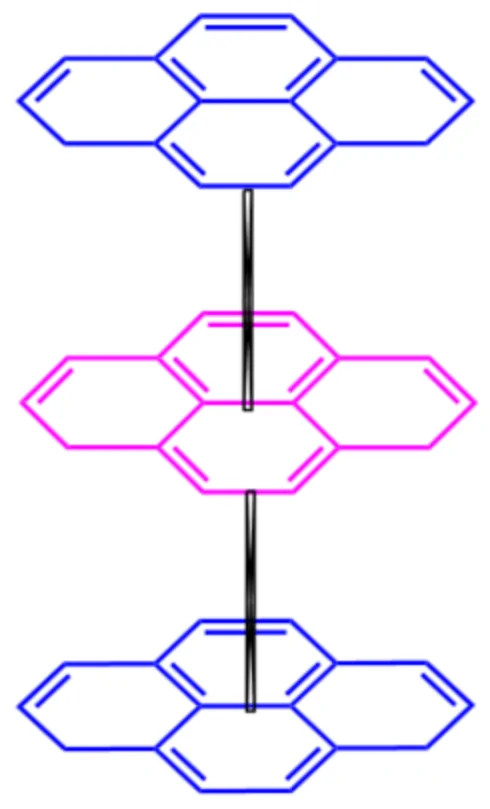
Simplified diagram of π-π stacking. Based on [122].

A π-π stacking interaction forms between two aromatic rings (one positively charged and one negatively charged), where the energy interaction is small (~8–12 kJ/mol) and thus easy to convert [51]. They are utilized in thermally-triggered self-healing supramolecular polymer networks [121]. A simplified schematic representation of π-π stacking interaction is presented in Figure 11.

Similarly to host–guest interactions, in recent years there have been very few reports on the use of π-π interactions as the sole mechanism in PUs worth mentioning [123]. A clear lack of interest in this type of interaction compared to, for example, disulfide bonds or multiple hydrogen bonds is likely associated with the fabrication of materials exhibiting insufficient mechanical strength and stiffness [124,125]. Consequently, research has shifted toward the development of hybrid systems, primarily incorporating disulfide bonds [126], multiple hydrogen bonds or the addition of nanofillers such as graphene [127] or cellulose nanocrystals [128], in order to promote a synergistic effect between different interactions, enabling higher self-healing efficiency and mechanical strength.

##### Ionic Bonds

**Figure 12:**
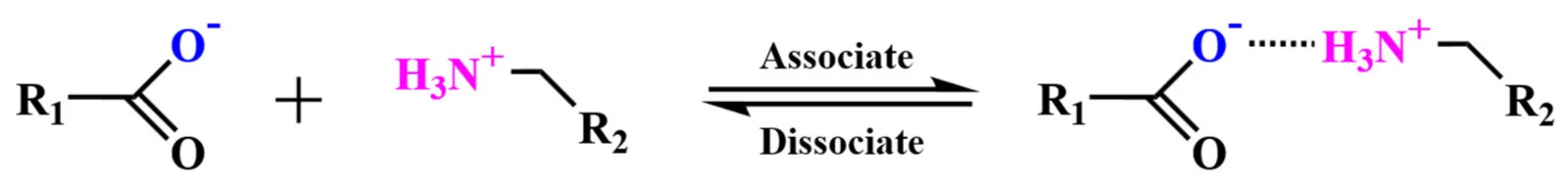
Ionic bond associate/dissociate. Based on [52].

Ionic interactions are created between positively charged cations that lose electrons and negatively charged anions that gain electrons, forming ionomers in the polymer matrix (Figure 12) [52]. Ionomers contain up to 15 mol% ionic groups that aggregate into clusters acting as reversible crosslinks [129]. Upon high impact, frictional heating is generated between the material’s surface and the projectile and is transferred to the nearest damaged location of the polymer network, leading to localized melting. The melted area then merges as highly mobile polymer chains interdiffuse across the interface [117].

Joo et al. [130] engineered a high-performance triboelectric nanogenerator (TENG) device using ionic polyurethane based on polycaprolactone (PCL), HDI and imidazolium ionic liquid diol. Ionic interactions between imidazolium cations and the PU backbone facilitate autonomous self-healing (healing efficiency of ~88% at 75 °C for 1 h), while simultaneously increasing the tribopositive behavior of the friction layer, which boosts power density (from 33 to 437 mW/m^2^). Moreover, the TENG device demonstrated capacitor-charging capabilities, charging capacitors of 1, 4.7, 10 µF within 60 s up to 5.7, 1.6 and 0.7 V, respectively. When connected to an array of 55 blue and 55 red LEDs, it successfully powered the LEDs, highlighting its potential for practical energy-harvesting applications. In another work [131], electrostatic interactions between trifunctional carboxylic acid and tertiary amino groups were used as a non-covalent crosslinking network within polyurethanes, exhibiting excellent tensile strength (20.49 MPa) along with high healing efficiency (97% at 60 °C for 1 h), stretchability (1007%) and toughness (50.38 MJ/m^3^). By integrating carbon nanotubes (CNTs), a flexible sensor was fabricated showing remarkable strain sensitivity and stability for wearable flexible electronics.

### 2.3. Comparative Analysis of Dynamic Covalent and Non-Covalent Self-Healing Mechanisms

The self-healing performance of polyurethanes depends not only on the type of reversible bond, but also on their exchange rate, distribution within the polymer network, and the mobility of the surrounding chains [132]. Dynamic covalent and non-covalent interactions provide complementary routes to intrinsic self-healing in polyurethane networks, but they differ in the balance between network integrity, chain mobility, and the conditions required for recovery [133]. Selected examples from Section 2.2.1, Dynamic Covalent Bonds and Section 2.2.2. Non-Covalent Bonds that illustrate the differences discussed are summarized in Table 1.

Dynamic covalent interactions, including ditelluride/diselenide/disulfide bonds, imine bonds, boroxine bonds, alkoxyamine bonds, and Diels–Alder bond, can reconnect polymer chains and help to restore the network after damage. Their main advantage is that they can recover network connectivity through bond exchange rather than relying only on chain diffusion and physical reassociation [134]. For example, disulfide exchange can occur under relatively mild conditions, whereas Diels–Alder networks often require heating to promote bond dissociation and subsequent rearrangement. Therefore, strong bonds alone do not ensure effective self-healing. The exchange rate must be compatible with the chain mobility and the time available for the damaged surfaces to come into contact [135]. Dynamic covalent bonds are particularly useful in coatings, adhesives, the biomedical field and as elastomers that require good mechanical strength, resistance to creep and the possibility of reprocessing [136].

Non-covalent interactions, including multiple hydrogen bonds, ionic interactions, metal–ligand coordination and host–guest interactions, can also contribute to self-healing by reforming when damaged surfaces are brought into contact. These interactions may enable repair at room temperature or under mild heating. They can also act as sacrificial bonds, breaking under stress and dissipating energy before permanent damage occurs. However, their reversible nature may reduce long-term stability under continuous loading, high humidity or elevated temperatures. In addition, strong supramolecular interactions can restrict chain mobility by promoting the formation of densely packed hard domains. This means that bond reversibility alone is not sufficient for effective healing; polymer chains must also be able to move across the damaged interface. Multiple hydrogen bonds and ionic interactions should therefore provide sufficient strength during use while allowing rearrangement during repair. Metal–ligand coordination offers further control through the selection of metal ions and ligands, although water, pH and competing ligands can affect the stability of these interactions [137].

**Figure 13:**
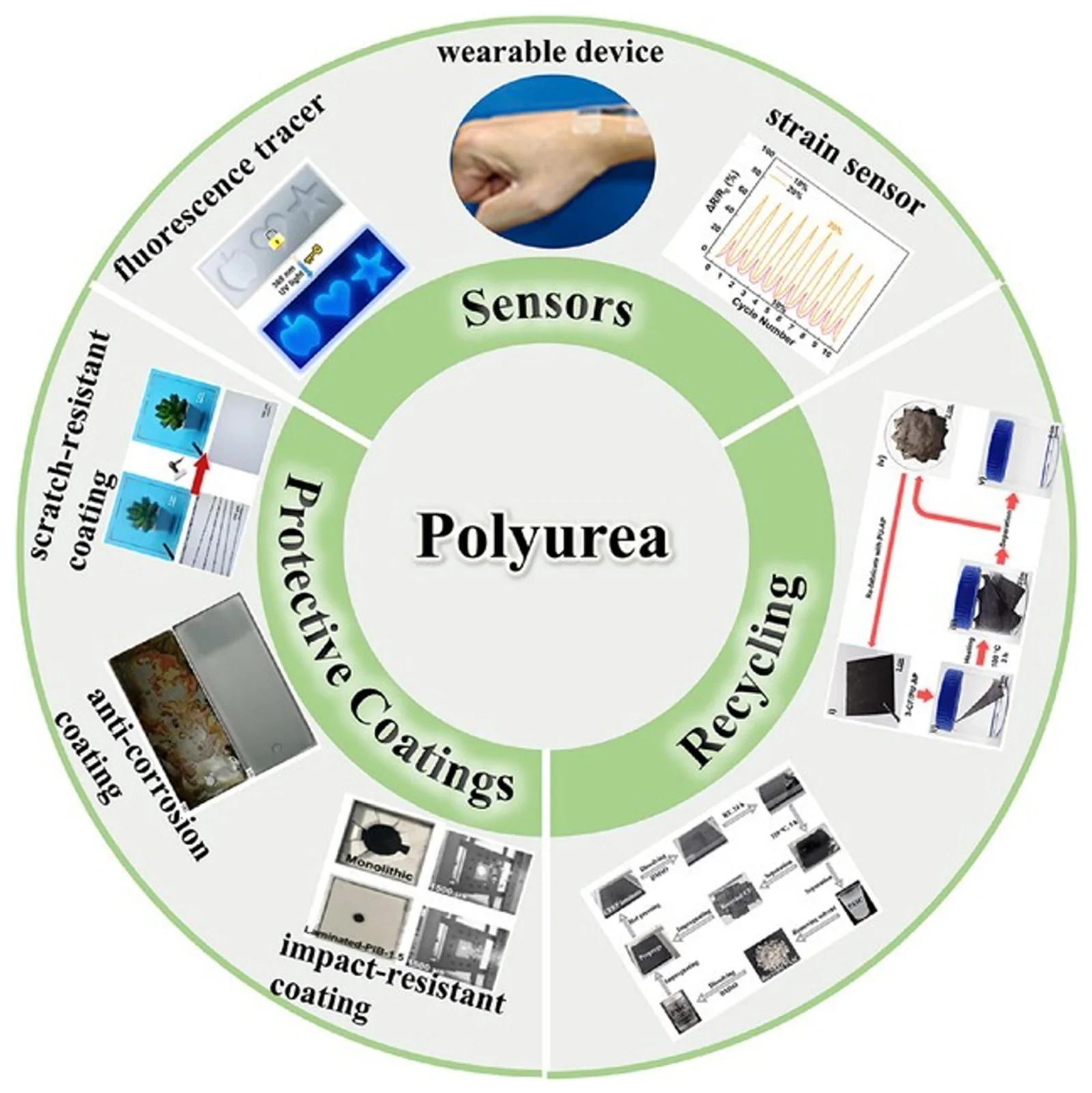
Application areas of mechanically robust self-healing polyurea. Reprinted with permission from [140]. Open Access—Creative Commons CC BY 3.0.

The hard- and soft-segment structure of PUs also plays an important role in self-healing. Hard segments, formed from diisocyanates or short-chain diols and chain extenders, often associate through hydrogen bonding and dipolar interactions, creating physical crosslinks that improve stiffness and tensile strength. However, excessive hard-segment content or strong hard-domain interactions can restrict chain movement and slow the contact and interdiffusion needed to repair damaged surfaces. As a result, a crack may close without fully recovering the original mechanical properties. Increasing the soft-segment content generally improves flexibility and chain mobility, which can support faster healing. However, too much soft-segment content may reduce strength, increase creep and impair dimensional stability [138,139]. A similar balance applies to crosslink density. Highly crosslinked networks provide better resistance to deformation but restrict the molecular rearrangements required for healing, whereas networks with fewer crosslinks may heal more easily but show lower mechanical stability. The optimal composition therefore depends on the intended application, the glass transition temperature of the soft segments, and the temperature at which the hard domains begin to relax or dissociate. These factors are particularly important when designing materials for applications ranging from flexible elastomers to structural adhesives and protective coatings, presented in Figure 13 [140].

Based on these relationships, several general design principles can be identified. (i) For self-healing at room temperature or under mild heating, low glass transition soft segments and sufficient chain mobility should be combined with moderately strong hydrogen-bonding or ionic interactions. This approach is suitable when rapid repair is a priority, although creep resistance may be limited [48]. (ii) For coatings and adhesives requiring high mechanical strength, dynamic covalent bonds can be combined with non-covalent interactions. The former help to maintain network connectivity, while the latter support faster reassociation at the damaged interface. This strategy is most suitable when the required activation temperature is compatible with the substrate and other material components [46,48]. (iii) Hard-segment content and crosslink density should be adjusted to provide the required mechanical performance without unnecessarily restricting chain mobility. Both tensile testing and stress-relaxation or dynamic mechanical analysis can help to assess this balance [48,141]. (iv) When combining different dynamic interactions, their activation conditions and effects on phase separation should be considered. A fast non-covalent interaction may restore contact between damaged surfaces, while slower covalent bond exchange can contribute to the recovery of mechanical strength. However, this combination is beneficial only when the different mechanisms can operate under compatible conditions [46,48]. (v) Healing efficiency should always be reported together with the measured property, damage geometry, healing temperature and time, applied pressure, and number of healing cycles. Comparisons between different studies also require consistent definitions of healing efficiency and sufficiently similar test conditions [142].

The studies discussed above illustrate the variety of molecular strategies used to achieve self-healing in polyurethane networks through dynamic covalent bonds and non-covalent interactions. To facilitate comparison of these approaches, Table 1 summarizes representative studies, highlighting the types of interactions used in the self-healing process. This overview provides a basis for comparing different design strategies and identifying the key factors behind self-healing performance.

### 2.4. Repeated Healing Cycles and the Trade-Off Between Healing Efficiency and Mechanical Performance

The evaluation of self-healing polyurethane materials should distinguish between the recovery achieved after a single damage-healing event and the retention of performance over repeated cycles. Although a high initial healing efficiency demonstrates that a particular damage event can be repaired under the reported conditions, it does not necessarily establish the long-term durability of the material. The relationship between healing efficiency and mechanical performance is influenced by the interaction between dynamic bonding mechanisms and the architecture of the polymer network. Molecular mobility and reversible interactions can assist the reestablishment of contacts across damaged interfaces, whereas the network must retain sufficient integrity to provide the mechanical properties required for its intended use. Consequently, the design of self-healing polyurethanes involves balancing the accessibility of the healing mechanism with the preservation of mechanical performance [48].

Repeated damage and healing cycles can reveal limitations that may not be apparent from single-cycle experiments. Although intrinsic self-healing polymers rely on reversible interactions that can reform after damage, their ability to undergo repeated repair without significant loss of performance must be verified experimentally [143]. Where available, changes in healing efficiency, tensile properties, fracture resistance, and other relevant functional parameters should be evaluated across successive cycles under clearly defined conditions. However, differences in testing protocols and the limited reporting of multi-cycle performance make it difficult to compare results systematically and draw general conclusions about long-term healing durability [144].

In several studies, the mechanical recovery of self-healing materials was assessed using stress–strain tests after three healing cycles, following scratching of the damaged surfaces [145,146,147,148,149,150,151]. The results reported in selected studies are presented in the following Table 2.

As shown in Table 2, the mechanical recovery of self-healing polyurethanes depends on the types of dynamic bonds and the applied healing conditions. Among the investigated systems, the polyurethane based on metal–ligand coordination showed the highest retention of mechanical properties over repeated healing cycles. After simulated sunlight exposure using a 50 W xenon lamp for 30 min, its tensile strength decreased only slightly, from approximately 28 MPa in the pristine state to 27.5 MPa after the first healing cycle and 26.5 MPa after the third cycle. The strain decreased from approximately 1770% to 1750% and 1630%, respectively, while the healing efficiency remained high at 98.6% and 95.5% [146]. Similarly, the polyurethane containing disulfide bonds and treated with near-infrared (NIR) irradiation retained a tensile strength of approximately 33 MPa after the first cycle and 32.5 MPa after the third cycle, compared with 35 MPa for the pristine material. Its healing efficiency remained close to 90% after repeated healing [148].

Polyurethanes combining different dynamic bonds also showed promising results, although their performance declined with repeated healing. For the system containing Diels–Alder and imine bonds, tensile strength decreased from approximately 27.5 MPa to 25 MPa after the first cycle and 21 MPa after the third cycle, while healing efficiency decreased from 94.3% to 79.2% [145]. A polyurethane combining disulfide and imine bonds exhibited a similar trend, with tensile strength decreasing from approximately 13 MPa to below 12 MPa and then below 10 MPa. Its healing efficiency decreased from 92.5% to 73.4% after the third cycle [147].

The Diels–Alder-based systems further illustrate the differences in mechanical recovery. One material exhibited a decrease in tensile strength from 6.55 MPa in the pristine state to 5.23 MPa after the first healing cycle and 3.78 MPa after the third cycle, with healing efficiency decreasing from 71% to 38% [149]. In comparison, a polyurethane combining Diels–Alder and disulfide bonds retained healing efficiencies of 90.6% and 71% after the first and third cycles, respectively, while its tensile strength decreased from approximately 10 MPa to 8.5 MPa and 7 MPa [150]. Another Diels–Alder-based system showed a healing efficiency of 96.4% after the first cycle and 71.4% after the third cycle, with tensile strength decreasing from approximately 14 MPa to 13.5 MPa and 10 MPa, respectively [151].

Overall, these results indicate that high healing efficiency after the first cycle does not necessarily ensure stable mechanical performance during repeated repair. Systems based on metal–ligand coordination and disulfide bonds showed relatively small changes in tensile strength, whereas some Diels–Alder-based materials exhibited more pronounced reductions in healing efficiency [146,148]. However, these differences cannot be attributed solely to the type of dynamic bond, as the healing temperature, treatment time, activation method and polymer network structure also influence the results. Therefore, the design of self-healing PUs should consider both the initial healing efficiency and the retention of mechanical properties over repeated healing cycles [149].

### 2.5. Reprocessing and Recyclability of Self-Healing Polyurethanes

Reprocessing is an important feature of intrinsically self-healing polyurethanes, as it allows the material to be reshaped and reused after its initial service life. Unlike conventional self-healing tests, which typically involve repairing a crack or reconnecting cut surfaces, reprocessing experiments usually involve cutting or shredding the polyurethane into small fragments and integrating them into a continuous specimen, most commonly by hot pressing. This process requires sufficient mobility of the polymer chains and reversible interactions that allow the polymer network to reorganize under the applied temperature and pressure. Depending on the material design, these interactions may include dynamic covalent bonds, such as disulfide or oxime-carbamate bonds, and reversible non-covalent interactions, such as hydrogen bonds. The recyclability of the resulting material is generally assessed by comparing its mechanical properties before and after reprocessing [152]. The selected examples described below are summarized in Table 3.

The recyclability of self-healing polyurethanes depends on the dynamic interactions within the polymer network and the applied reprocessing conditions. For example, polyurethane elastomers containing multiple hydrogen bonds and disulfide bonds were recycled by hot pressing at 100 °C under 5 MPa for 30 min. After three shredding and reprocessing cycles, their stress–strain behavior remained comparable to that of the pristine material. The material could also be recovered using N,N-Dimethylacetamide (DMAc) at 80 °C, and the resulting film supported a 5 kg load without breaking. Even after three recoveries, FTIR spectra showed no substantial changes in the main characteristic peaks [153].

Similarly, a lignin-based polyurethane (DLPU4.1) containing hydrogen-bonding interactions was reprocessed at 105 °C under 10 MPa for 10 min. FTIR and XRD analyses indicated that its main chemical functionalities and crystalline structure remained largely unchanged. Tensile-strength retention reached 94.0%, 92.4%, and 88.1% after the first, second, and third reprocessing cycles, respectively. The material also retained self-healing performance, with tensile-strength healing efficiencies of 92.1%, 88.3%, and 80.5% over three successive damage-repair cycles [154].

The importance of dynamic network design was further demonstrated by comparing two non-isocyanate polyurethane elastomers. A bio-based non-isocyanate polyurethane elastomer containing dual dynamic network (DDCAN-NIPU) consisting of disulfide bonds and imine bonds was hot-pressed at 140 °C under 20 MPa for 10 min. Its tensile strength increased from 3.22 MPa in the pristine state to 3.45, 3.99, and 4.34 MPa after three reprocessing cycles, corresponding to 107.1%, 124.0%, and 134.8% retention. However, elongation at break decreased from 234% to 206%, 165%, and 121%. Under the same conditions, SDCAN-NIPU (single dynamic network), based only on dynamic imine bonds, showed a gradual decrease in tensile strength from approximately 2.70 MPa to 2.34, 2.21, and 2.02 MPa, corresponding to 86.7%, 81.9%, and 74.8% retention. This comparison suggests that combining different dynamic covalent interactions can improve strength retention during reprocessing, although changes in elongation and other properties must also be considered [155].

A different approach was demonstrated for supramolecular interaction-based polyurethane elastomers (SPUEs), in which hydrogen bonds provide the dynamic interactions responsible for network rearrangement. Instead of hot pressing, the materials were dissolved in DMF at 110 °C for 20–30 min, followed by solvent removal to obtain new films. After the first recycling cycle, tensile strengths reached 59, 58, 58, and 25 MPa for SPUE1-SPUE4, corresponding to 155%, 134%, 94%, and 48% of their original values, respectively. After three cycles, the corresponding strengths were 31, 42, 44, and 22 MPa, representing 87%, 100%, 71%, and 42% retention. These results highlight the substantial influence of polymer composition and network structure on recycling performance [156].

Overall, these studies show that self-healing polyurethanes can often be reprocessed several times while retaining substantial mechanical performance. However, the outcome depends on the dynamic bonding chemistry, network structure, and processing conditions. Moreover, an increase in tensile strength does not necessarily indicate that all mechanical properties are preserved. Therefore, recyclability should be evaluated using multiple parameters, including tensile strength and elongation at break, while self-healing performance should be assessed separately on reprocessed samples to establish whether the material retains its ability to repair damage [157,158].

## 3. Hybrid Molecular and Nano-Interfacial Design Strategies

Although hundreds of studies have investigated the influence of various intrinsic self-healing mechanisms on the properties of polyurethanes, these advances remain insufficient to meet the requirements for large-scale implementation and practical applications. The development of highly efficient self-healing polyurethanes continues to face several major challenges, including complex and poorly scalable synthetic routes, the use of organic solvents and petroleum-based components, limited healing efficiency and/or insufficient mechanical performance. These limitations have highlighted that the overall performance of these materials depends not only on the presence of dynamic bonds, but also on the way different reversible interactions cooperate within the polymer network [46,48,52]. While individual healing mechanisms have been extensively studied, increasing attention is currently directed toward hybrid systems that combine multiple dynamic interactions to exploit their synergistic effect. Moreover, there is also an interfacial hybridization concept, involving the incorporation of functional nanofillers such as graphene derivatives, carbon nanotubes, metal and ceramic organic frameworks or MXenes. Those additives interface with the PU matrix and can modulate chain mobility, energy dissipation, dynamic bond exchange, or stimulus-responsive healing. Understanding the design principles behind those mechanisms is essential for developing next-generation polyurethane materials that simultaneously exhibit high mechanical strength, efficient damage recovery and long-term durability [46,52,159,160].

### 3.1. Molecular Hybridization Through Complementary Dynamic Interactions

The design of self-healing polyurethane materials has gradually evolved from systems based on a single reversible interaction toward hybrid polymer networks incorporating multiple dynamic mechanisms. Herein, hybrid polyurethane materials are defined as polyurethane networks that integrate complementary covalent and non-covalent interactions within the polymer architecture (molecular hybridization). The underlying concept is based on the hierarchical organization of dynamic bonds [161,162].

Reversible covalent bonds, such as Diels–Alder adducts, act as relatively stable crosslinking points that preserve the integrity of the polymer network and enable recovery after mechanical damage. Owing to their relatively high bond energy compared with supramolecular interactions, they function as the primary load-bearing connections within the polymer network, maintaining dimensional stability and permanent crosslinking under different conditions. Their reversible nature allows bond dissociation upon external stimuli, providing repeated healing without permanent degradation of the material. However, because bond exchange requires thermal activation, healing is generally slower and often occurs at elevated temperature, highlighting the need for complementary dynamic interactions with a faster response time [161].

In contrast, supramolecular interactions, particularly multiple hydrogen bonds, exhibit rapid association–dissociation dynamics under ambient conditions. Unlike dynamic covalent bonds, these reversible interactions continuously break and reform without requiring significant external stimuli, allowing the polymer network to respond quickly to mechanical damage. These types of bonds act as sacrificial bonds, where weak and reversible interactions dissociate under mechanical loading, dissipating applied energy and redistributing local stress, protecting the primary load-bearing network from irreversible damage. Following removal of the applied stress, the hydrogen bonds readily reestablish, promoting rapid network reconstruction and efficient recovery of mechanical properties. Despite their outstanding healing kinetics, the limited bond strength of supramolecular interactions generally results in lower stiffness and tensile strength compared with covalently crosslinked systems [163,164].

### 3.2. Synergistic Integration of Multiple Interactions

#### 3.2.1. Hydrogen Bonding–Disulfide Bond Hybrid Networks

Among various molecular hybridization strategies, the combination of hydrogen bonding and dynamic disulfide chemistry has emerged as one of the most effective approaches for designing self-healing polyurethanes. This strategy brings out the complementary roles of the two interactions. UPy-urea provides high strength but limits chain mobility and self-healing; disulfide bonds enable room-temperature self-healing but may reduce mechanical strength. However, together, hydrogen bonds rapidly reform across damaged interfaces, promoting chain diffusion and crack closure, while disulfide exchange gradually reconstructs the covalent network and restores its mechanical integrity. Consequently, the synergistic effect addresses the intrinsic trade-off between healing efficiency and mechanical robustness [46,161,162].

The synergistic combination of dynamic disulfide bonds and hydrogen bonding has been exploited to develop self-healing polyurethane elastomers for flexible sensing applications. Wen et al. [165] reported a PU elastomer in which disulfide bonds were introduced through a disulfide-containing chain extender, while hydrogen-bonded urethane groups provided additional reversible intermolecular interactions. The optimized PU exhibited a tensile strength of 3.35 MPa and an elongation at break of 334%, while surface cracks could be healed within 3 h at room temperature and completely repaired within 15 min at 60 °C. To impart electrical functionality, the PU surface was subsequently modified with a 3,4-ethylenedioxythiophene polystyrene sulfonate (PEDOT:PSS) conducting layer, yielding flexible sensors with a gauge factor of 14.75 at 70–90% strain. The sensors successfully detected various human motions, demonstrating how molecularly engineered self-healing PU networks can serve as mechanically durable substrates for wearable electronics.

Another group [166] designed bio-based polyurethane with multiple hydrogen bonds by incorporating UPy-modified cellulose and disulfide bonds by introducing 4,4’-diamino-diphenyl disulphide. Herein, a dense hydrogen-bonding network provides reversible, sacrificial crosslinks that rapidly reform upon damage and disulfide bonds act as dynamic covalent linkages, undergoing bond exchange reactions that restore polymer chain continuity across crack interfaces. This supramolecular elastomer showed self-healing efficiency of 93% under low-temperature conditions (12 h at −10 °C), although its mechanical performance leaves much to be desired (tensile strength below 1 MPa). As a single-electrode friction nanogenerator, the voltage output (20 V) was stable across various impact frequencies (1–5 Hz) and could charge a commercial capacitor leading to powering an LED lamp and LED arrays, before and after damage. What is more, by arranging a system consisting of copper wires, supramolecular elastomer film, signal processing module, Bluetooth transmission module, speaker, and a digital display and shell printed by a 3D technique, a high-precision wireless target shooter was successfully assembled.

Hydrogen-disulfide exchange between 2-ureido-4-pyrimidone moieties and bis(4-hydroxyphenyl) disulfide was also successfully implemented in another work [162] to enhance room-temperature self-healing and mechanical toughness in the presence of ionic liquids [(1-ethyl-3-methylimidazolium bis(trifluoromethylsulfonyl)imide (EMIM TFSI)/3-ethyl-1-methyl-1H-imidazol-3-ium Bis(fluorosulfonyl)azanide (EMIM FSI)]. The resulting polymer and ionic conductor exhibited exceptional properties: 700/850% stretchability, 34/30 MPa fracture strength and ~78/87 MJ/m^3^ toughness, respectively. On top of that, they were integrated into self-healing multimodal sensors for electronic skin, which could independently detect mechanical (10–50% strain) and thermal (30–60 °C) stimulation before and after damage, without signal loss.

#### 3.2.2. Disulfide Bonds and Metal–Ligand Coordination

Beyond disulfide and hydrogen-bond combinations, other dynamic interactions can be introduced to further tailor the balance between chain mobility and healing kinetics. Disulfide exchange enables dynamic reconstruction of the covalent polymer network, while metal–ligand coordination can act as a stronger yet reversible physical crosslink that increases intermolecular cohesion and dissipates mechanical energy. This complementary behavior is particularly attractive because increasing the density of coordination bonds can improve strength and toughness, while the dynamic disulfide bonds facilitate chain mobility and network rearrangement during healing [167,168].

A recent example [169] reported a polyurethane–urea elastomer incorporating disulfide bonds, urea-based hydrogen bonding, and Zn^2+^–ligand coordination, together with a UV-crosslinked network. In this design, disulfide exchange provides dynamic network rearrangement, whereas hydrogen bonding and metal coordination introduce additional reversible crosslinks that contribute to cohesion, energy dissipation, and mechanical strength. The resulting material could be rapidly UV-cured within 100 s while maintaining high optical transparency (>92%), a tensile strength of 11.8 MPa, and an elongation at break of 2423%. The elastomer also exhibited rapid recovery and thermally activated self-healing, reaching 93% healing efficiency after 8 h at 80 °C. The combination of self-healing capability, high resilience, and rapid UV curability makes this polyurethane–urea elastomer a promising candidate for applications in flexible sensing and wearable electronics.

Cheng et al. [170] incorporated a PU elastomer with disulfide bonds and metal–ligand coordination bonds, resulting in exceptional toughness (85 kJ/m^3^) and high healing efficiency above 85%. The self-healing PU-based TENG delivered 12 µA, 120 V, and 38.5 nC from a 2 × 2 cm^2^ device, achieving a power density of 2.1 W/m^2^ at 20 MΩ. Even after damage, and subsequent self-repair, the device retained 95% of its electrical power. The TENG device demonstrated excellent durability and practical applicability, maintaining stable performance over 20,000 cycles and successfully illuminating LED lights while enabling noncontact self-powered sensing.

#### 3.2.3. Dual Dynamic Covalent Networks

A complementary strategy is the integration of two different dynamic covalent chemistries. Disulfide bonds are attractive dynamic crosslinks because their relatively low bond dissociation energy enables reversible disulfide exchange and molecular rearrangement under moderate conditions. In contrast, Diels–Alder chemistry, typically based on the reversible reaction between furan and maleimide groups, provides thermally reversible covalent crosslinks that can increase network cohesion and mechanical stability, while retro-DA reactions at elevated temperature enable network rearrangement. The combination of these two chemistries therefore introduces dynamic covalent bonds with different exchange kinetics and activation requirements, providing multiple pathways for network reconstruction, and can broaden the accessible healing conditions, especially for anticorrosion applications [171,172].

Mao et al. [173] proposed a self-healing PU combining dynamic disulfide bonds [2,5-bis(4-hydroxyphenyl) disulfide, BHPS] and Diels–Alder interactions [furandimethanol (FDME) and bismaleimide (BMI)]. Disulfide metathesis enables scratch healing under mild conditions (~60 °C), while D-A bonds provide high crosslink density for load bearing and undergo retro D-A near 100 °C to allow full network rearrangement and reprocessing. During self-healing evaluation, the scratch was still visible after healing at 60 °C for 3 h; however, at 100 °C for 30 min, not only did the cut disappear, but the tensile strength recovered to 25.5 MPa, corresponding to a healing efficiency of 82%. Moreover, as a ~200 µm anticorrosion coating, healed films recovered low-frequency impedance to ~3.6 × 10^9^ Ωcm^2^ (compared to ~4.1 × 10^9^ Ωcm^2^ in the original sample), showing almost complete restoration of the surface integrity and anticorrosion properties.

**Figure 14:**
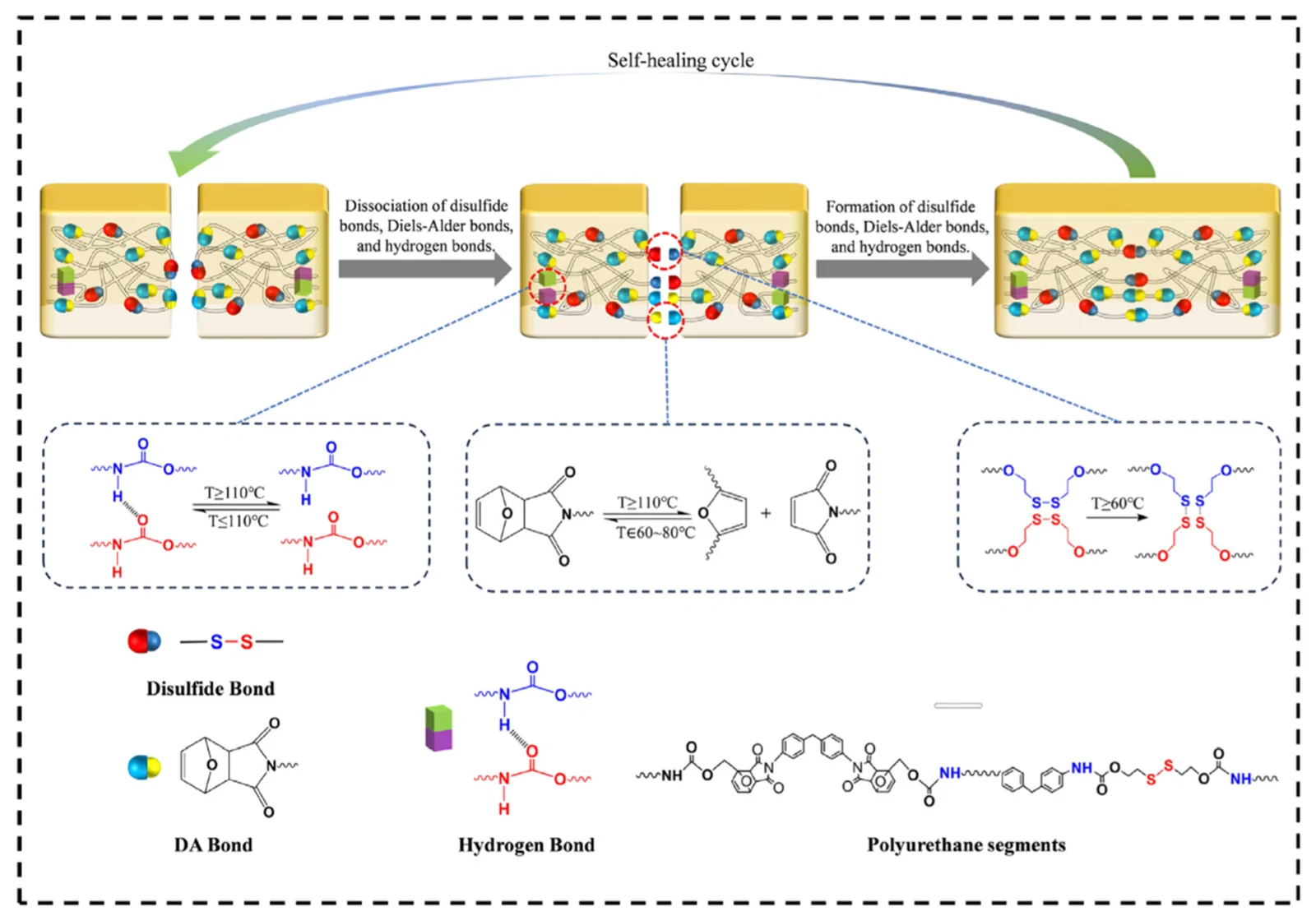
Self-healing mechanism schematic of polyurethane containing dual dynamic covalent bonds. Reprinted with permission from [174]. © 2026 by the authors. Licensee MDPI, Basel, Switzerland.

In another work [174], a self-healing polyurethane (SSDA-PU) was developed, incorporating furfuryl alcohol and bismaleimide as a Diels–Alder interaction source and bis(2-hydroxyethyl) disulfide as a disulfide bond source. The self-healing process was attributed to the synergistic action of disulfide bond exchange, hydrogen-bond dissociation/reformation, and the reversible D-A reaction, providing multiple routes for network rearrangement and damage repair (Figure 14). The optimized SSDA-PU exhibited a tensile strength of 21.1 MPa and an elongation at break of 755.17%. The material achieved an initial self-healing efficiency of 90.38% and retained 59.21% efficiency after three consecutive damage–healing cycles. The combination of disulfide exchange and thermoreversible D-A chemistry also enabled healing over a relatively broad temperature range (60–120 °C), while the dynamic network provided good recyclability. Overall, this study demonstrates that combining dynamic covalent bonds with different exchange mechanisms can provide a more versatile healing network than relying on a single dynamic bond, offering an effective strategy for balancing mechanical performance, healing efficiency, and recyclability in self-healing polyurethane systems.

Another example of dual dynamic covalent networks was reported by Ye et al. [147], who developed a self-healing polyurethane incorporating both dynamic disulfide (2,2′-diaminodiphenyl disulfide) and imine bonds (Schiff base reaction between terephthalic aldehyde and ethanolamine) called PUAS. The combination of these two exchangeable covalent linkages provided complementary pathways for network rearrangement and damage repair, while maintaining high mechanical integrity. The resulting polyurethane exhibited a tensile strength of 12.95 MPa and an elongation at break of 623%, together with a high fracture energy of approximately 100.7 kJ/m^2^.

#### 3.2.4. Multi-Dynamic Molecular Networks: Integration of Three or More Reversible Interactions

Beyond binary combinations of dynamic interactions, recent studies have explored multi-dynamic molecular networks in which three or more reversible interactions are integrated within a single polyurethane architecture. Such designs aim to exploit the distinct kinetics, bond strengths, and activation conditions of different dynamic motifs to achieve a more balanced combination of mechanical robustness, molecular mobility, and self-healing capability [158,175].

For example, Tahir et al. [176] engineered hybrid hydroxyl-terminated polybutadiene-based (HTPB) polyurethane coatings based on quadruple dynamic mechanisms between disulfide exchange, hydrogen bonding, thiourethane linkages and metal-coordination (Zn^2+^-N/O) bonds. During self-healing evaluation, a cut interface disappeared completely after 24 h at 25 °C, demonstrating sufficient polymer chain mobility and dynamic bond reformation at the damage interface. This process promoted interchain entanglement that led to macroscopic crack closure, underscoring the synergistic role of thiourethane/hydrogen exchange and metal–ligand interactions in enabling autonomous recovery. The tensile strength increased slightly (1.90 to 2.54 MPa) due to effective reinforcement driven by dynamic zinc cation–ligand coordination. According to the Tafel experiment, increasing ZnCl_2_ content improved the anticorrosive performance as an anticorrosive coating and enhanced the self-healing efficiency of polyurethane coating through Zn^2+^–urethane coordination interactions, with optimum self-healing effect observed at 0.4 mmol ZnCl_2_, beyond which healing efficiency decreased. The rate of corrosion drops from ~0.15 mm/year for pristine coating to 0.036 mm/year for hybrid coating, highlighting the protective effect of metal–ligand passivation and the development of a denser, more compact polymer network.

In another work, a triple dynamic effect was achieved by Zhang et al. [177] by synthesizing a PU-based [PTMEG, methylene diphenyl diisocyanate (MDI), 4,4′-dihydroxybiphenyl (BP)] material combining π-π interactions and hydrogen bonds together with phenolic urethane bonds through one-pot polymerization. During self-healing performance tests, it was concluded that phenolic urethane bonds exhibit dynamic behavior at room temperature, accompanied by the formation of intermolecular hydrogen bonds. In addition, π-π stacking interactions between benzene rings reduce intermolecular distances, enabling the synergistic action of multiple reversible interaction, resulting in a self-healing efficiency close to 95% (5 h at 30 °C) and a tensile strength of almost 15 MPa. This material was used as a positive triboelectric layer in a TENG device (PUB-TENG), consisting of polydimethylsiloxane (PDMS) as a negative layer and a copper sheet acting as a conductive electrode layer. Such a design was successfully used to drive 26 green LEDs and 3 LEDs with shape memory performance, with an increasing dielectric constant with a decreasing diphenyl unit. Future plans are focused on designing high-temperature monitoring and alarm warnings in the automotive industry.

One design [178] was based on crosslinked polyurethane (HDI, PEG 4000) solid–solid phase-change materials (Cu^2+^-OUPCMs) prepared by introducing three types of dynamic bonds—oxime–urethane interactions, metal–ligand coordination bonds and hydrogen bonds within one polyurethane system [dimethylglyoxime (DMG), anhydrous copper chloride (CuCl_2_), and pentaerythritol (PTOL)]. Synthesized Cu^2+^-OUPCMs exhibited moderate phase-change properties with a melting enthalpy around 83 J·g^−1^ (compared to ~176 J·g^−1^ for pure PEG 4000), reflecting restricted crystallization due to hydrogen bonding and crosslinked network formation. Cu^2+^ incorporation slightly increased the phase transition temperatures and thermal and shape stability. Cu cations endowed the material with photothermal conversion performance and exceptional mechanical strength above 20 MPa, while the synergistic effect between triple dynamic interactions provided a self-healing efficiency close to 90%. The excellent self-healing, reprocessing, and thermal stability of Cu^2+^-OUPCMs reduce material waste and enhance sustainability, making them promising for solar thermal storage, battery thermal management, and electronic heat dissipation applications.

On the other hand, an even more complicated system was previously designed by Wang et al. [179], who prepared a bio-based polyurethane elastomer by combining Diels–Alder adducts, disulfide bonds, hydrogen bonds and van der Waals forces, enabling excellent self-healing efficiency (95% at 60 °C) and recyclability. When used as the friction layer in a flexible triboelectric nanogenerator, the device achieved a power density of 0.52 mW/m^2^ and, after self-healing, almost full recovery of output voltage (~36 V). These results confirm that the dynamic networks preserve device performance after damage. This work illustrates how a careful and thoughtful design approach to TENG materials could extend device lifetime and reduce waste in energy harvesting applications intended for outdoor wearable devices.

The studies discussed in this section illustrate the diversity of strategies for incorporating multiple bond interactions and nanofiller components into self-healing polyurethane networks. These approaches combine different molecular interactions with the specific properties of nanomaterials to tailor healing behavior and functional performance. For an overview of the approaches described above, Table 4 summarizes representative studies on self-healing polyurethane networks based on multiple bond interactions, highlighting their key design features and reported healing performance. This comparison provides a basis for assessing the advantages and limitations of different hybrid strategies.

### 3.3. Nano-Interfacial Hybridization

The interfacial hybridization concept involves the incorporation of functional nanofillers into polyurethane matrices. In particular, the incorporation of carbon-based nanofillers, MXenes and metal–organic frameworks has opened new pathways toward multifunctional materials with performance levels that are difficult to achieve using conventional approaches. These functional additives interact with the PU matrix and can modulate chain mobility, energy dissipation, dynamic bond exchange, and stimulus-responsive healing, thereby contributing to the design of advanced self-healing materials [40,41,180]. Moreover, in our opinion, covalent organic frameworks also deserve attention as a promising class of porous, structurally tunable materials, although their application in self-healing polyurethanes remains relatively unexplored. Nevertheless, their distinctive structural characteristics and potential for functionalization suggest promising opportunities for future developments in this field [181].

#### 3.3.1. Carbon-Based Nano Additives

According to the dimensional division, carbon-based nano additives can be divided into zero-dimensional (0D) nanomaterials such as fullerenes and nanodiamonds, one-dimensional (1D) nanomaterials such as carbon nanotubes (CNTs), nanorods and nanofibers, two-dimensional (2D) nanomaterials including graphene and three-dimensional (3D) nanomaterials represented by nanohybrids [182]. Among these additives, the most popular ones for self-healing polyurethanes according to Scopus or WoS are graphene derivatives.

##### Graphene and Its Derivatives

**Figure 15:**
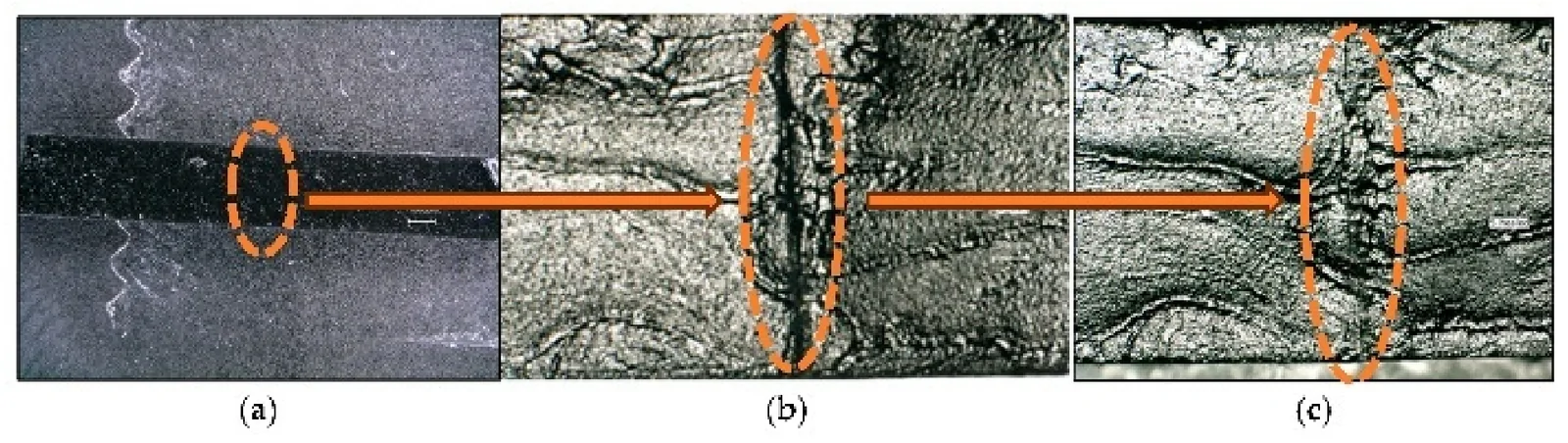
PU specimens with 2% graphene content. (**a**) Material before cutting, (**b**) material after cutting, and (**c**) material after the self-healing process at 80 °C for an hour. Reproduced under terms of the CC-BY license [183]. © 2025 by the authors. Licensee MDPI, Basel, Switzerland.

Few studies have investigated non-functionalized graphene as a primarily additive in self-healing polyurethane. We undertook such a synthesis ourselves by introducing 1–3 wt% graphene nanoparticles into polyurethane enhanced by multiple hydrogen bonds from UPy. The self-healing mechanism is based on the dissociation and reformation of hydrogen bonds, supported by increased polymer-chain mobility upon heating. The primary function of graphene was to reinforce the material and ensure good processability. Mechanical characterization demonstrated that all nanocomposites possessed high self-healing efficiencies, ranging from 88 to 91%. Notably, the polyurethane nanocomposite containing 2% graphene (Figure 15a) exhibited the highest initial mechanical strength (approximately 5 MPa) compared to that of a pristine PU (~2 MPa), although these figures are not among the highest, while still maintaining high self-healing efficiency (88%). Furthermore, a complete recovery of a material after the cut (Figure 15b) in the 2% graphene material was achieved after thermal treatment at 80 °C for 1 h (Figure 15c), indicating a rapid healing response. Three-dimensional printing was successfully employed to evaluate the processability of the materials and its influence on their self-healing performance. The results indicate that the 3D printing process does not compromise the intrinsic regeneration ability, confirming that the material can be utilized while maintaining the desired dimensional accuracy and geometric complexity [183].

**Figure 16:**
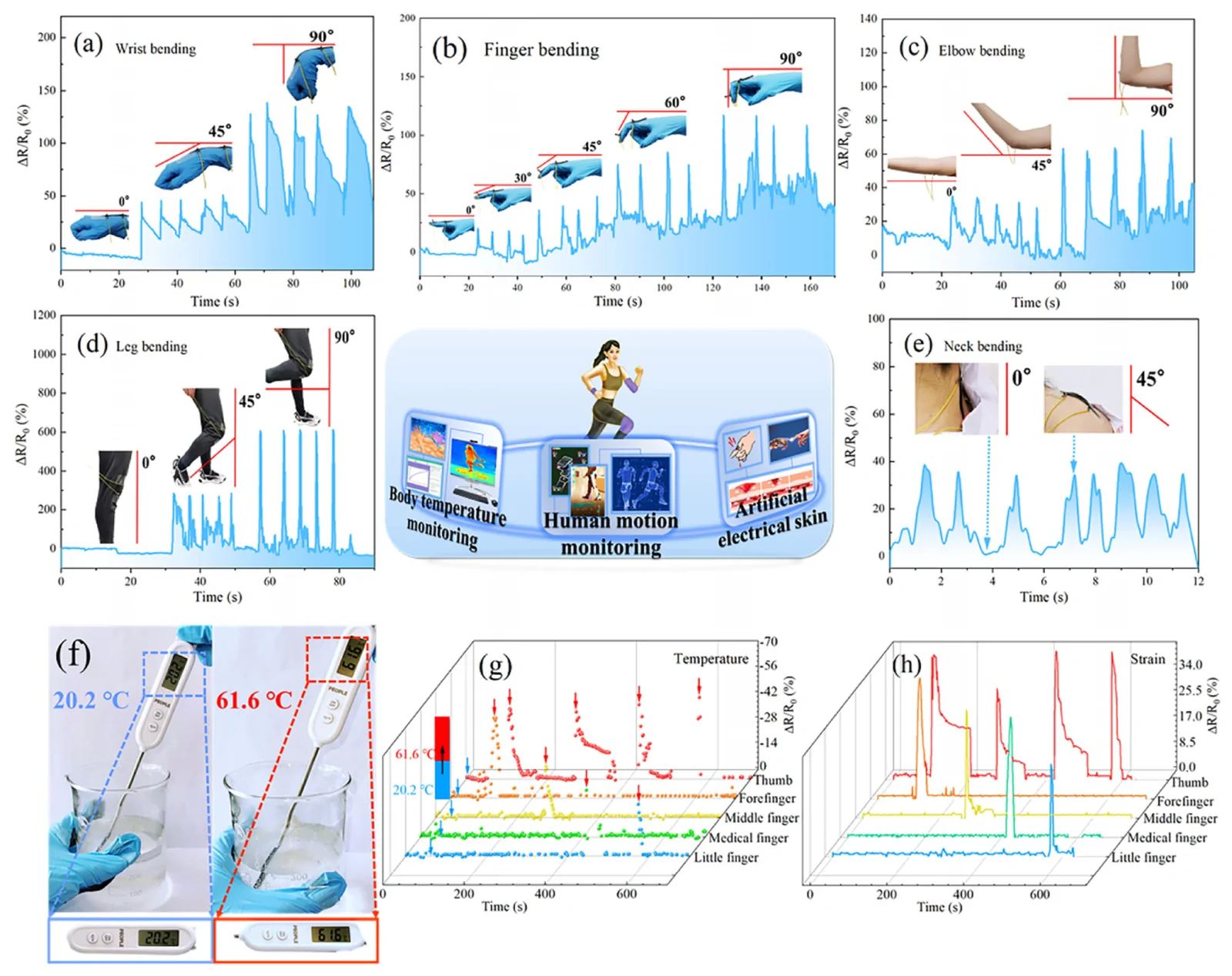
Applications of the PUU/E-GNP sensor to human motion monitoring: the ΔR/R_0_ response of (**a**) wrist bending, (**b**) finger knuckle bending, (**c**) elbow bending, (**d**) knee bending, and (**e**) neck bending. The images (**f**) of two beakers with 20.2 °C and 61.6 °C, and real-time monitoring of resistance response of the glove alternately touching the beakers: (**g**) temperature response and (**h**) strain response. Reprinted with permission from [184]. © 2024 The Authors. Published by Elsevier Ltd.; Creative Commons CC-BY license.

In another example [184], the authors developed a highly stretchable and self-healing PUU/E-GNP nanocomposite by incorporating amine-functionalized graphene nanoplatelets into a hydrogen-bonded PUU matrix. The optimized 7 vol% E-GNP sensor exhibited a tensile strength of 19.73 MPa, 361.76% strain, and a gauge factor of 17.57, while maintaining stable performance over 20,000 cycles. The conductive graphene network enabled simultaneous strain and temperature sensing, while the hydrogen-bonded matrix allowed recovery of 65.36% tensile strength, 77.15% elongation, and 77.80% electrical conductivity after healing. The resulting multifunctional sensor was used for real-time monitoring of human motion and skin temperature. As shown in Figure 16a–e, the nanocomposite-based sensor exhibits rapid and stable responses to different bending angles and human motions, including bending of a wrist, finger, elbow, knee, and neck. As an integrated glove, it worked both as a real-time monitoring (Figure 16f) temperature sensor (Figure 16g) and strain sensor (Figure 16h), demonstrating its potential for wearable and biomedical applications.

Graphene oxide has been successfully incorporated into polyurethane matrices for many years, as evidenced by a substantial number of scientific publications [185,186]. In our opinion, the following, among others, deserves special attention: Zhu et al. [39] reported a polyurethane–graphene oxide supramolecular network combining disulfide exchange and sextuple hydrogen bonding for self-healing. The GO nanofiller enhanced interfacial interactions and load transfer, yielding a tensile strength of 78 MPa, toughness of ~505 MJ/m^3^, elongation of 1273%, and ~89% self-healing efficiency after 24 h at 25 °C. The material also enabled an NIR-actuated crawling robot that recovered its motion after damage, demonstrating potential for durable, flexible actuators. Huang et al. [187] developed polyurethane phase-change materials (GO-Cu^2+^-OUPCMs) integrating a triple-effect mechanism based on hydrogen bonds, coordination bonds, and oxime–urethane linkages, together with covalently grafted graphene oxide. Hydrogen bonds provide reversible physical crosslinking, coordination bonds enable dynamic crosslinking and mechanical reinforcement, and oxime–urethane linkages undergo bond exchange to reconstruct the polymer network. Covalently attached GO further enhances mechanical strength and thermal conductivity while preserving self-healing, resulting in ~92% healing efficiency and a tensile strength of 18.65 MPa. In Li-ion battery tests, GO-Cu^2+^-OUPCMs reduced the surface temperature from 62.5 °C to 40.2 °C after the first cycle and ~58 °C after the third cycle, demonstrating effective thermal regulation and improved cycling stability. This idea provides a new insight into designing phase-change materials in efficient thermal energy storage and management.

**Figure 17:**
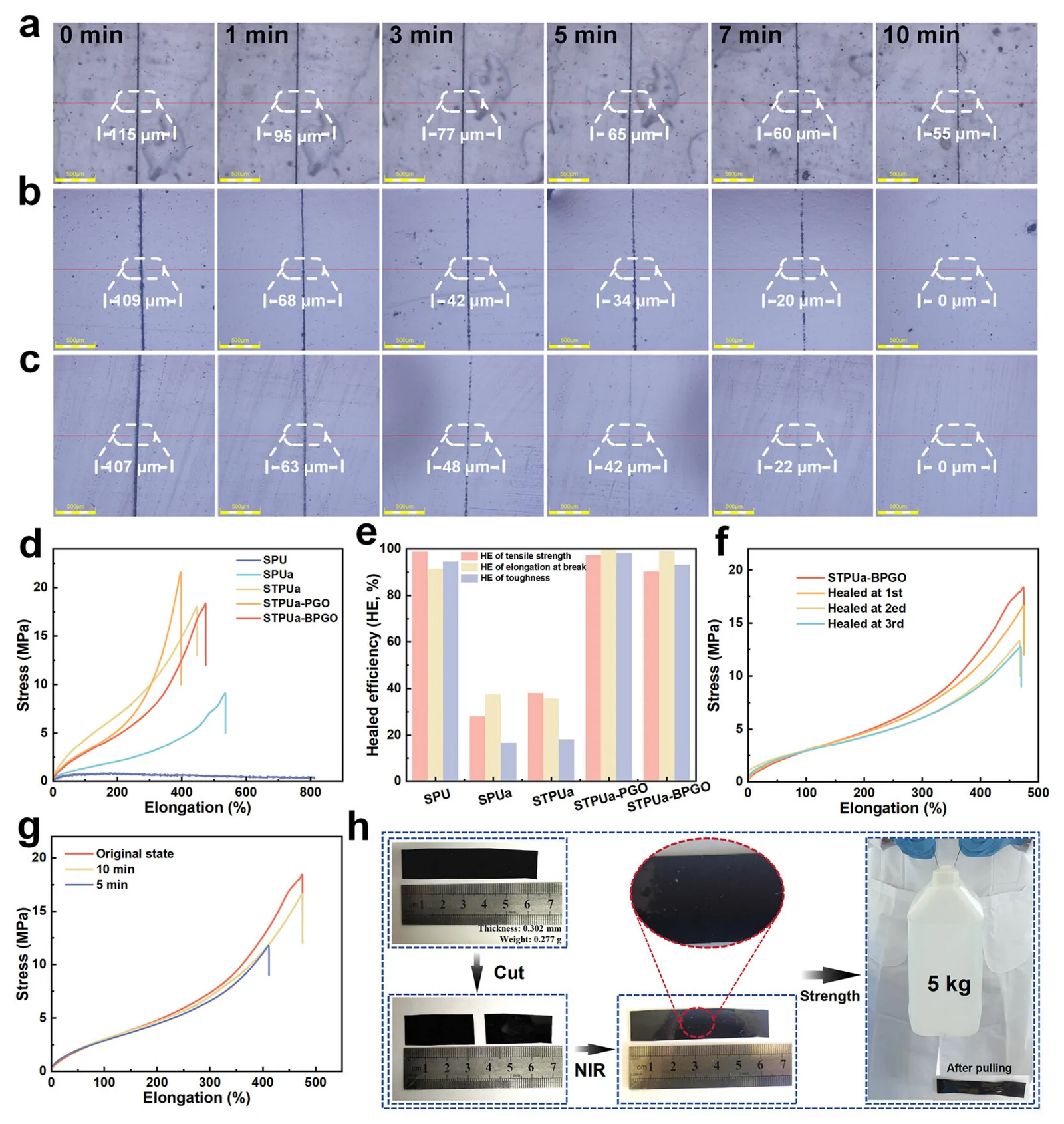
Self-healing property of the polymers. Images of the scratch healing process of (**a**) STPUa, (**b**) STPUa-PGO and (**c**) STPUa-BPGO. (**d**) The stress–strain curves of the samples. (**e**) The HE of SPU, SPUa and STPUa healed at 80 °C for 24 h, and STPUa-PGO and STPUa-BPGO healed under NIR for 10 min. The stress–strain curves of healed STPUa-BPGO (**f**) for multiple times and (**g**) at different times. (**h**) Images of STPUa-BPGO healing process and pulling up heavy objects after healing. Reprinted with permission from [189]. © 2026 Wiley-VCH GmbH.

Beyond graphene and graphene oxide, reduced graphene oxide (rGO) has also emerged as a promising carbon-based nanofiller for enhancing the multifunctionality and self-healing performance of polyurethane-based materials. Tian et al. [188] developed a polyurethane/functionalized reduced graphene oxide (M-PU) coating using a hierarchical hybrid crosslinking strategy based on dynamic Diels–Alder bonds and multifunctional maleimide-modified rGO. After 30 days in 3.5 wt% NaCl, M-PU exhibited a three-order-of-magnitude increase in low-frequency impedance compared with the unmodified PU, indicating markedly improved electrochemical corrosion resistance. The hierarchical network also reduced mass loss by 50.4% after 48 h of cavitation erosion and by 64.6% after 24 h of tribological wear. Importantly, the dynamic crosslinks provided energy dissipation, self-repairing capability, and recyclability, demonstrating the potential of hybrid PU/rGO coatings for multifunctional protection of marine components. On the other hand, Cui et al. [189] developed a dopamine-functionalized rGO polyurethane coating that combined photothermal self-healing, superhydrophobicity, corrosion protection, and antifouling functionality. As shown in Figure 17a–c, the coating retained its hierarchical surface morphology and superhydrophobicity after abrasion and exhibited stable photothermal responses under repeated NIR irradiation (Figure 17e). The stress–strain relation is shown in Figure 17d,f,g. The coating achieved a self-healing efficiency of 90.29% within 10 min while simultaneously pulling 5 kg object after healing (Figure 17h) and maintained an impedance of 2.80 × 10^8^ Ω cm^2^ after 30 days in 3.5% NaCl, demonstrating its potential for multifunctional marine protective applications.

##### Carbon Nanotubes

Carbon nanotubes are structures of single graphite sheet (single-walled nanotubes, SWNTs) rolled seamlessly into a cylindrical form or multi-layers of graphene (multi-walled nanotubes, MWNTs) cylinders arranged coaxially, resembling the layered structure of tree rings, with either open or closed ends. Their exceptional properties, including high Young’s modulus approaching 1 TPa, tensile strength of 100 GPa, and high electrical and thermal conductivity make them excellent multifunctional nanofillers [190,191].

The introduction of CNTs into hybrid polyurethane networks significantly alters both the physical and chemical properties of the material. From a mechanical perspective, CNTs act as nanoscale reinforcements that generally raise modules, tensile strength, and toughness through efficient load transfer. At the same time, CNTs influence the dynamics of the polymer network. Their presence can restrict chain mobility, hindering the healing process to some extent if the network becomes too rigid. Thus, CNT concentration, dispersion and surface chemistry need to be strictly controlled and optimized to preserve a balance between physical reinforcement and healability [192].

**Figure 18:**
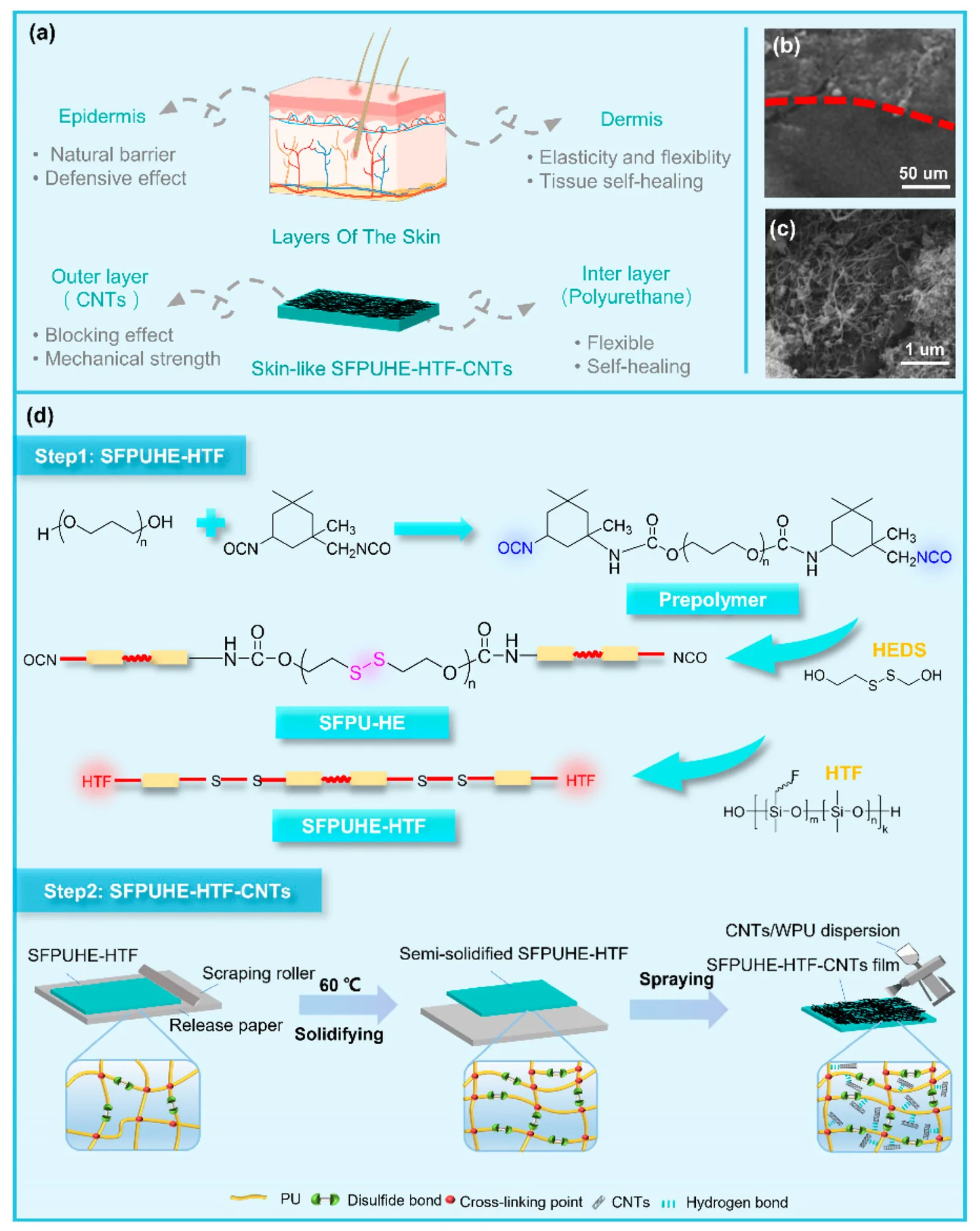
(**a**) Schematic diagram of the design concept for preparing the skin-inspired, bilayer solvent-free polyurethane/carbon nanotubes composites (SFPUHE-HTF-CNTs). The SEM images of (**b**) the bilayer structure and (**c**) the outer CNT layer. (**d**) The fabrication process of SFPUHE-HTF-CNTs. Reproduced under terms of the CC-BY license [193]. © 2022 by the authors. Licensee MDPI, Basel, Switzerland.

In the work of Kong et al. [193], a bilayer coating system with disulfide bonds and fluorinated polysiloxane segments was designed as presented in Figure 18a,d and enabled efficient self-healing under mild conditions (60 °C for 16 min or UV irradiation at an energy value of 200 for 48 h). Additionally, CNTs/WPU dispersion was sprayed onto a bilayer coating (Figure 18b) to provide electrical conductivity and to work as a corrosion protection layer with real-time damage monitoring. The CNT-based outer layer (Figure 18c) was characterized by a low corrosion current density (8.94 × 10^−9^ A⋅cm^2^), a relatively positive corrosion potential (−0.38 V), and a high impedance modulus |Z| = 4.79 × 10^5^ Ω⋅cm^2^). Notably, the coating retains strong barrier properties after healing, with |Z| remaining at 4.54 × 10^4^ Ω⋅cm^2^, confirming effective restoration of protective function. What is more, damage-induced changes in CNT spacing alter conductivity, enabling real-time monitoring of defects, which supports applications in intelligent anticorrosion coatings and flexible sensing systems.

CNTs were also incorporated into a PU elastomer based on a dynamic boronic ester bond (nitrogen-coordinating cyclic boronic diester compounds, NCBCs) and a hydrogen bond (1,8-menthane diamine, MD) to prepare conductive composite and a wearable sensor capable of identifying human activities such as elbow/finger bending, facial expression changes, speaking, and walking. CNTs act as mechanical reinforcement fillers, improving tensile strength (~17.5 MPa for PU/CNT compared to 15.4 MPa for PU/NCBC/MD/CNT) and stiffness. The strain-sensing behavior reveals two distinct linear regions with gauge factors of 7.04 and 23.66, indicating high sensitivity and elasticity [194].

CNTs can also introduce electromagnetic wave absorption, as demonstrated by Zeng et al. [195]. Their ionic-liquid-functionalized CNT/PU composite, combining dynamic disulfide exchange and strong hydrogen bonding, exhibited 85–90% mechanical recovery after healing at 80 °C for 4 h. CNT incorporation increased tensile strength to ~12.5–22.5 MPa and enhanced thermal transport and healing kinetics. The composite also showed strong electromagnetic absorption (reflection loss < −40 dB; effective bandwidth ~4.2 GHz) and high extensibility (>1000%), enabling wearable strain sensing of finger, wrist, elbow, and knee movements. These properties may be useful for rehabilitation monitoring applications and highlight the potential of CNT/PU hybrids for multifunctional wearable electronics, EMI shielding, and durable self-healing sensing systems.

##### MXenes

**Figure 19:**
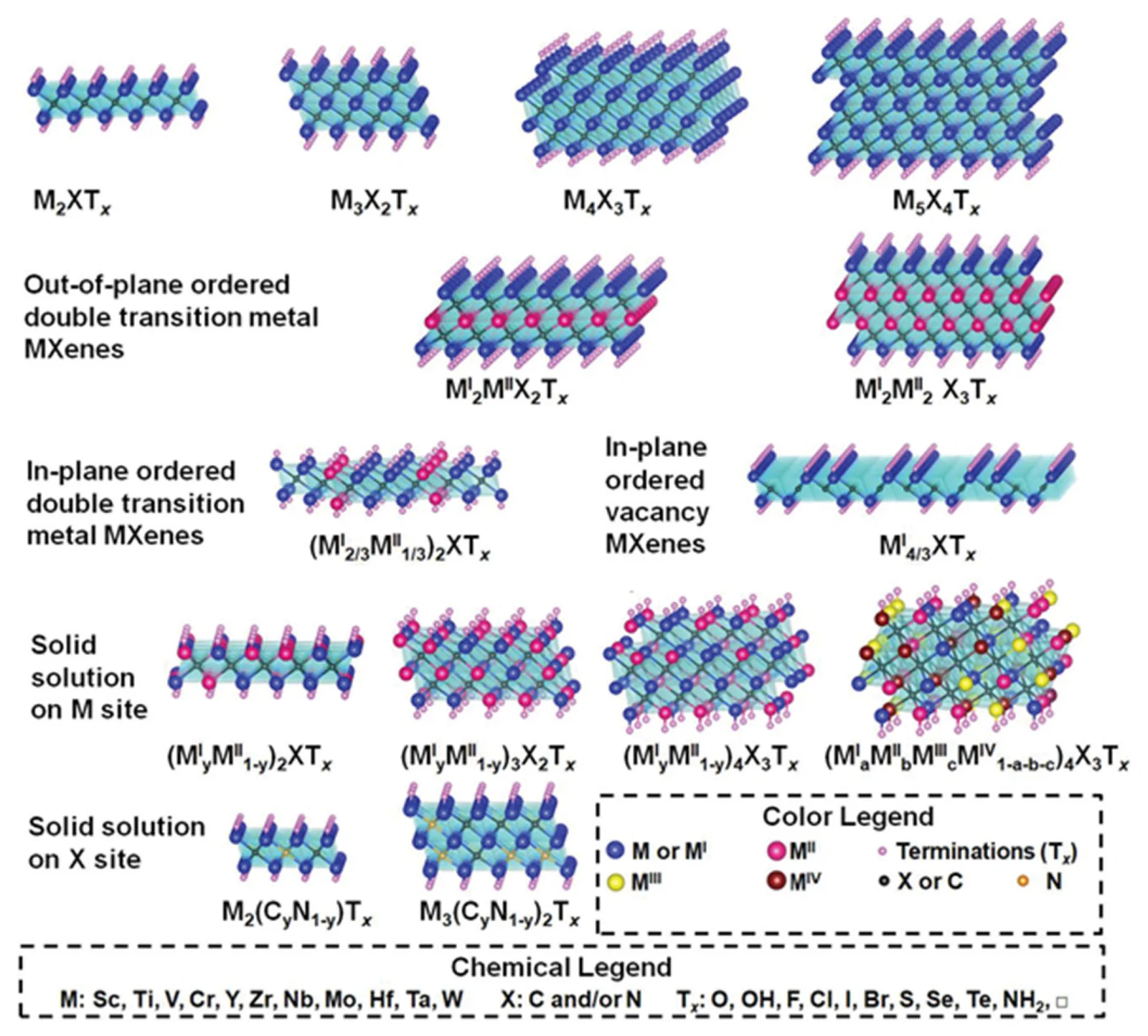
Typical MXene structures and compositions. MXenes have a formula of M_n+1_XnTx, where M is an early transition metal, X is C or N, and Tx represents surface terminations. The n value can vary from 1 to 4. The M sites can be occupied either by one, two, or more transition metal atoms, forming solid solutions or ordered structures. The ordered double transition metal MXenes exist as in-plane ordered structures (i-MXenes), e.g., (Mo 2/3Y1/3)2CTx,in-plane vacancy structures, e.g., Mo2/3CTx, and out-of-plane ordered structures (o-MXenes) where one or two layers of MII transition metal are sandwiched between layers of M I transition metal, e.g., Cr2TiC2Tx or Mo2Ti2C3Tx. Reprinted with permission from [205]. © 2021 Wiley-VCH GmbH.

The discovery of 2D transition metal carbides and/or nitrides called MXenes (M_n+1_Xn, where M symbolizes transition metals and X represent carbon and/or nitrogen) depicted in Figure 19 has opened up new possibilities that were not previously possible [196,197]. This is due to an impressive number of properties that these materials possess, including hydrophilic character, chemically tunable surfaces, metal-like conductivity, ion-holding capacity, and large surface area, among others [198]. The latest concepts which deserve special attention are wearable electronic fabrics [199], bioelectronic interfaces and implants [200], ultrafast energy storage systems [201], programmable desalination and ion-selective membranes [202], smart wound dressing and therapeutic biointerfaces [203], and machine-learning-assisted screening of catalysts [204].

**Figure 20:**
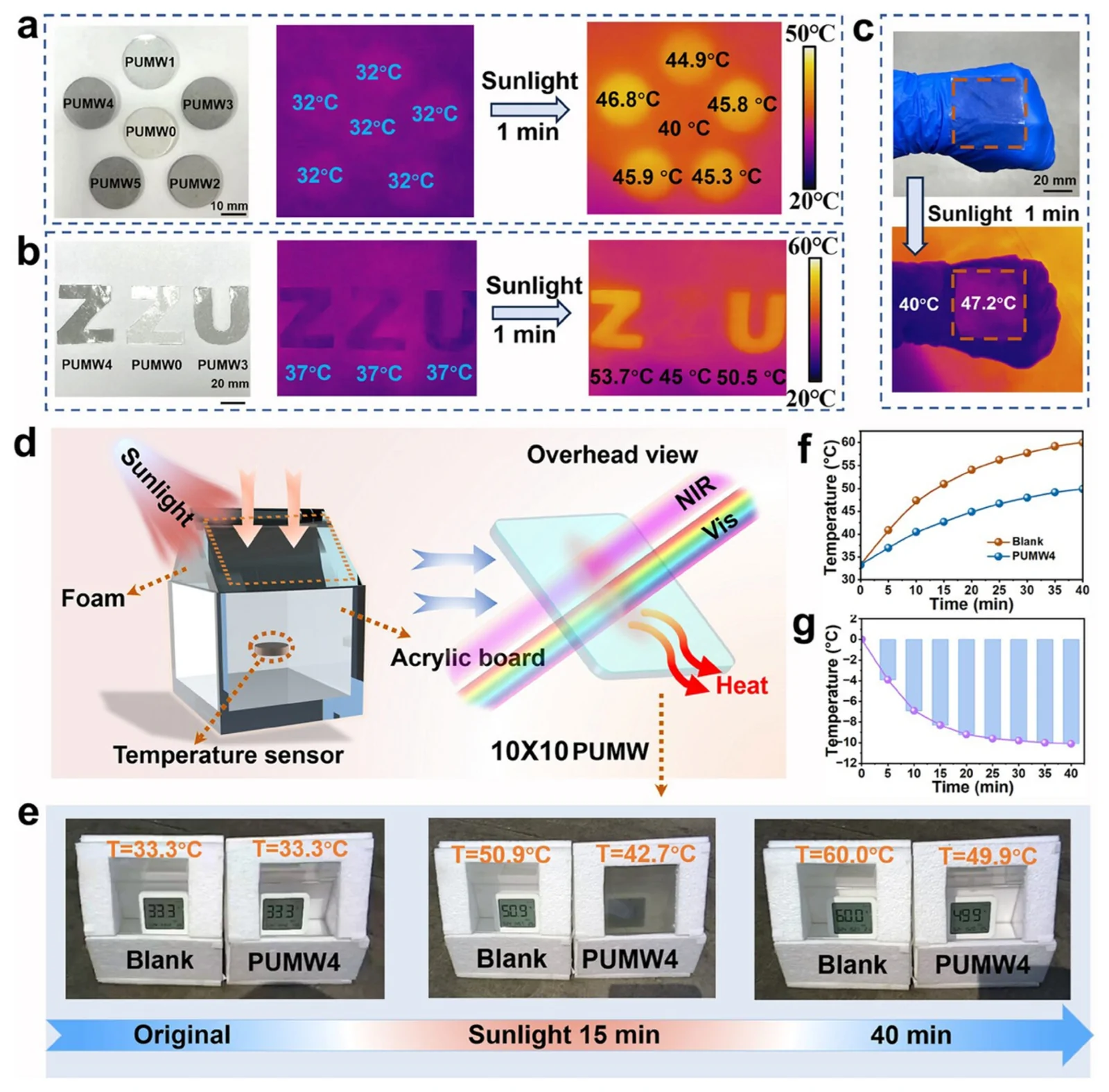
The thermal-management applications of PUMW films. (**a**,**b**) Photograph of different 1 mm thick PUMW samples. IR thermal images of different PUMW samples before and after solar irradiation for 1 min. (**c**) Photograph of a ≈200 µm thick PUMW4 applied onto the hand of a volunteer (top). IR thermal images after solar irradiation for 1 min (bottom). (**d**) Diagram and (**e**) photographs of the outdoor cooling performance test system. (**f**,**g**) Cooling performance of the model covered with PUMW4 in comparison with blank model. Reprinted with permission from [206]. © 2025 The Author(s). Advanced Science published by Wiley-VCH GmbH; Creative Commons CC BY 4.0.

Polyurethane with plasmonic-enhanced MXene (Ti_3_C_2_T_x_ nanosheets) and W_18_O_49_ has been designed in the form of transparent films, where a visible light transmittance (VLT) of ~79% has been achieved and reached 102 °C within 5 min and surface scratch disappeared after 2 min, along with tensile strength up to 30 MPa. Thus, exhibiting not only self-healing behavior and high mechanical performance, but also excellent photothermal conversion for energy-conserving applications [206]. The thermal-management performance of polyurethane films was demonstrated using sunlight exposure on circular samples and “ZZU” patterned samples (Figure 20a,b). One of the samples, PUMW4, exhibited a 6–9 °C higher temperature than pure PU, while a transparent PUMW4 film applied to a volunteer’s hand increased its temperature by 7.2 °C after 1 min (Figure 20c). In outdoor model-device tests (Figure 20d,e), the PUMW4 coating reduced the internal temperature by approximately 10 °C compared with the uncovered blank (Figure 20f,g). Combined with its stable photothermal and mechanical properties after prolonged outdoor exposure, PUMW4 shows potential for thermal-management applications, including building window coatings and flexible wearable heaters [206]. The second key difference lies in the physical reinforcing character of MXenes, which act as nanofillers and dynamic interaction hubs. By designing multi-level bonding networks within a single hybrid system, both high strength and efficient repairability can be achieved in a single material. To achieve controlled and rapid healing, as well as high tensile strength, a hybrid polyurethane-based photothermal material was prepared via chemical integration of MXene (Ti_3_C_2_T_x_ nanosheets) with polydopamine nanoparticles (dopamine hydrochloride). Such a design is used to explore their synergistic NIR photothermal response and integrated with a multi-level network of dynamic hydrogen bonds and oxime–carbamate linkages. The resulting nanocomposite exhibits high tensile strength of 58.8 MPa, elongation at break up to 790% and excellent toughness of approximately 149 MJ/m^3^, without compromising healing efficiency, which upon NIR irradiation (808 nm) heats to ~91 °C within 30 min at 1.00 W/cm^2^ with an efficiency of 82.7%. The proposed strategy of rapid on-demand repair via photothermal conversion offers a novel pathway for extending the use of self-healing materials in outdoor environments, particularly in flexible electronics, soft robotic, and smart protective coatings [207].

**Figure 21:**
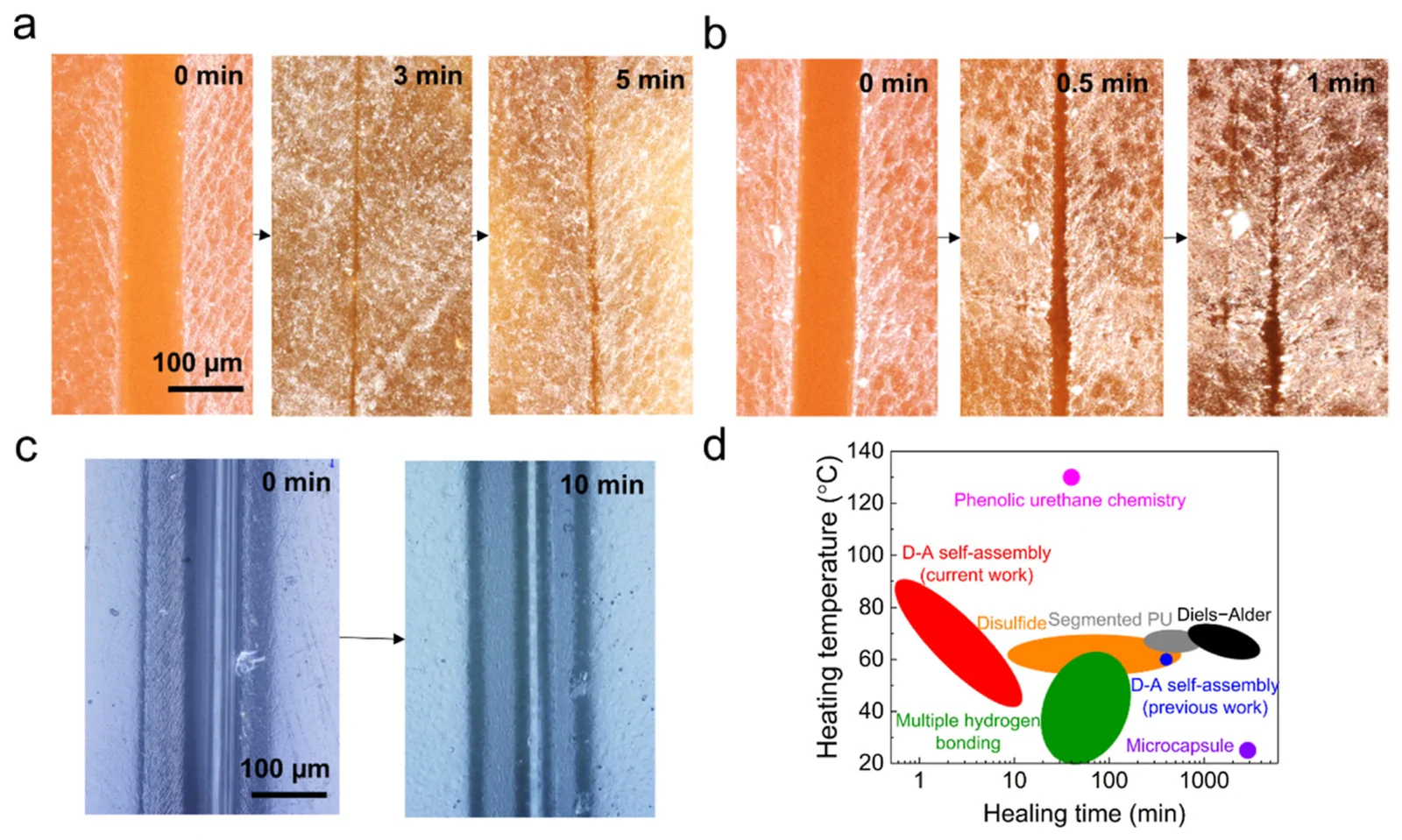
Self-healing properties of the notched DA-PU and PU films. (**a**) Optical microscope images of the notched DA-PU (left, 0 min) and healed films after 3 and 5 min (right) at 60 °C. (**b**) Optical microscope images of the notched DA-PU film showing fast disappearance of the scar during self-healing at 80 °C for 1 min. (**c**) Optical microscope images of the notched PU film showing slowly healing of the scar at 80 °C for 10 min. Scale bar: 100 μm. (**d**) Comparison of the self-healing properties of DA-PU with different repair mechanisms. Reprinted with permission from [214]. © 2023 The Authors. Published by American Chemical Society This publication is licensed under a Creative Commons Attribution 4.0 International License.

In the work of Wen et al., MXene plays a dual role in the system, forming a synergistic conductive network with ionic liquids that significantly enhances electrical conductivity up to 37.5 mS/m while also providing strong photothermal conversion for rapid and remote self-healing (efficiency above 80% under thermal or light activation). The material design relies on dynamic multiple hydrogen bonds introduced via imidazolidinyl urea together with ionic interactions (1-ethyl-3-methylimidazolium dicyanamide), enabling network reconstruction and energy dissipation during deformation. As a result, an optimized ionogel exhibits a tensile strength of approx. 2 MPa, elongation at break of approx. 590%, conductivity above 27 mS/m and gauge factor of 1.8. These combined properties make the material highly suitable for wearable strain/pressure sensors which has been experimentally proven, as shown in Figure 21a. Figure 21b,c shows that an ionogel-based sensing array could accurately detect both the position and magnitude of applied pressure, demonstrating its potential for human-motion monitoring systems, particularly in flexible and self-repairable electronic devices [208].

Another example of using MXenes in smart applications is designing a multifunctional fabric coating consisting of MXene, carbon nanotubes and polyurethane with hydrogen, disulfide and borate bonds. The dynamic covalent disulfide and borate exchange provide efficient reconnection after surface damage due to rearrangements of the molecular chains, whereas hydrogen bonds contribute to rapid network rearrangements. MXene nanosheets and CNTs form a highly conductive, interconnected network that enhances electrical sensitivity, electromagnetic interference shielding, and electrothermal performance, while also reinforcing the polymer matrix. The resulting fabric sensor exhibits a high gauge factor of 8.78 and strong EMI shielding with a minimum reflection loss of -57.6 dB at 7.6 GHz. This sensor allowed for monitoring the fingers bent at different positions, stable performance under repeatable 1000 cycle tests, and detectable relative resistance changes. These results demonstrate multifunctionality beyond sensing and highlight its potential for wearable smart devices and smart textiles, particularly in continuous infant healthcare monitoring and protection [209].

##### Metal–Organic Frameworks, MOFs

Metal–organic frameworks, nowadays also called coordination polymers (PCPs), are crystalline hybrid materials constructed from metal ions or clusters coordinated with organic linkers. They form highly ordered porous networks with exceptionally large surface areas and tunable structure. They can be classified based on dimensionality (1D, 2D, and 3D), topology, or composition (ZIF, ZMOF, MAF, MPF, bioMOF, and UiO), offering vast structural diversity and precise control over pore size and functionality [210]. The key features of MOFs include ultrahigh porosity (>7000 m^2^g^−1^), chemical tunability, and the ability to tailor host–guest interactions, which arise from coordination bonds and functionalized organic linkers. These properties enable applications in gas storage and separation, heterogenous catalysis, sensing, energy storage and drug delivery [211,212].

Given the vast number of MOF structures and their excellent properties, they are also slowly being introduced into PU as active multifunctional nanofillers. They enable additional dynamic interactions through reversible coordination and enhancing hydrogen bonds [213,214], which in turn enhances interfacial interactions and chain mobility, and they promote energy dissipation and crack propagation, as well as reversible deformation [215,216].

In analogy to PU/MXene systems, PU/MOF-based architectures are frequently explored for corrosion protection purposes. One recent study reported a multifunctional polyurethane-based superhydrophobic anticorrosion coating reinforced with graphene oxide (GO) on a dual-ligand cerium-based metal–organic framework (Ce-MOF) modified with polydopamine (PDA) referred to as GO/Ce-MOF@PDA. Graphene oxide provides a diffusion barrier, Ce-MOF introduces active inhibition and self-healing (via Ce ions and 2-mercaptobenzimidazole), and PDA enhances interfacial adhesion and nanoparticle dispersion. The composite architecture yields a highly dense coating (~55 µm thickness) with superhydrophobic behavior (contact angle > 150°) and strong substrate bonding (5B adhesion), while also significantly improving mechanical durability and wear resistance. Electrochemically, the coating exhibits an ultrahigh impedance modulus of 2.54 × 10^9^ Ω·cm^2^ initially, remaining at 3.82 × 10^9^ Ω·cm^2^ after 30 days in 3.5 wt% NaCl. Long-term salt spray exposure for 40 days shows minimal degradation and clear self-healing. Herein, 2-mercaptobenzimidazole and cerium ions generate insoluble precipitates and protective films at damaged regions, effectively suppressing corrosion of the aluminum substrate and enabling a smart self-healing response, in agreement with electrochemical findings [217].

A similar study reports a polyurethane-based composite reinforced with a 2-ureido-4[1H]-pyrimidinone-functionalized zinc imidazolate framework-7 (ZIF-7-UPy). The key mechanism relies on reversible multiple hydrogen bonding between UPy motifs and polymer chains acting as dynamic physical crosslinks. These supramolecular interactions significantly enhance mechanical performance, yielding a tensile strength of 48 MPa and toughness of 272 MJ/m^3^, and at the same time enabling efficient room temperature self-healing with ~87% recovery of original strength. The zinc imidazolate framework-7 nanoparticles function not only as reinforcing fillers but also as multifunctional nodes that improve network integrity and trigger release of corrosion inhibitors under acidic conditions. The incorporation of MOF enhanced anticorrosion performance, as compared to pristine PU, evidenced by an increase in the low-frequency impedance modulus |Z|_0.01 Hz_ from 2015 Ω·cm^2^ (for neat PU) to 7263 Ω·cm^2^. This effect is caused by the pH-responsive decomposition of MOF and release of benzimidazole inhibitors, which form protective films at damaged sites, leading to noticeable impedance recovery over 24 h and effectively suppressing corrosion even after 120 h exposure in 0.1 M HCl. Such a design highlights its potential for protective coatings in infrastructure and industrial systems requiring long-term durability and self-repair autonomous healing [218].

Zhang et al. [214] developed a self-healing, liquid-repellent polyurethane coating by combining donor–acceptor supramolecular interactions with hydrophobic MOF nanoparticles. The reversible electrostatic interactions and hierarchical hydrogen bonding enabled rapid self-healing (Figure 21a–c) of damaged coatings within 1 min at 80 °C, while providing strong adhesion to glass, metals, and PTFE. Incorporation of alkyl-functionalized MOFs and subsequent silanization generated a nanohierarchical superhydrophobic surface with self-cleaning properties and resistance to tape peeling and high-speed liquid impacts. Moreover, liquid repellency was recovered after damage and the coating maintained low ice adhesion over 50 icing/deicing cycles. The porous MOF structure also enabled the fabrication of fluorine-free slippery liquid-infused porous surfaces (SLIPS) with stable anti-icing performance, demonstrating the potential of this hybrid PU/MOF design for durable self-healing protective coatings with the shortest healing time compared to other reports (Figure 21d).

Finally, a self-healable and recyclable waterborne polyurethane membrane (WPU-SS-MOF) was developed by incorporating disulfide bonds and UiO-66-NH_2_ MOF for dye wastewater adsorption. The membrane showed pH-dependent adsorption capacities of 83.2 mg·g^−1^ for methyl orange and 42.4 mg·g^−1^ for Congo red, with removal efficiencies of 82.4% and 80.5% after five adsorption–desorption cycles, respectively. Increasing the number of MOF layers improved hydrophilicity and dye removal, with optimal performance at three to four layers. Although the membrane exhibited a high self-healing efficiency of 96.1%, healing required 24 h at 110 °C, which may limit its practical applicability [219].

##### Covalent Organic Frameworks, COFs

**Figure 22:**
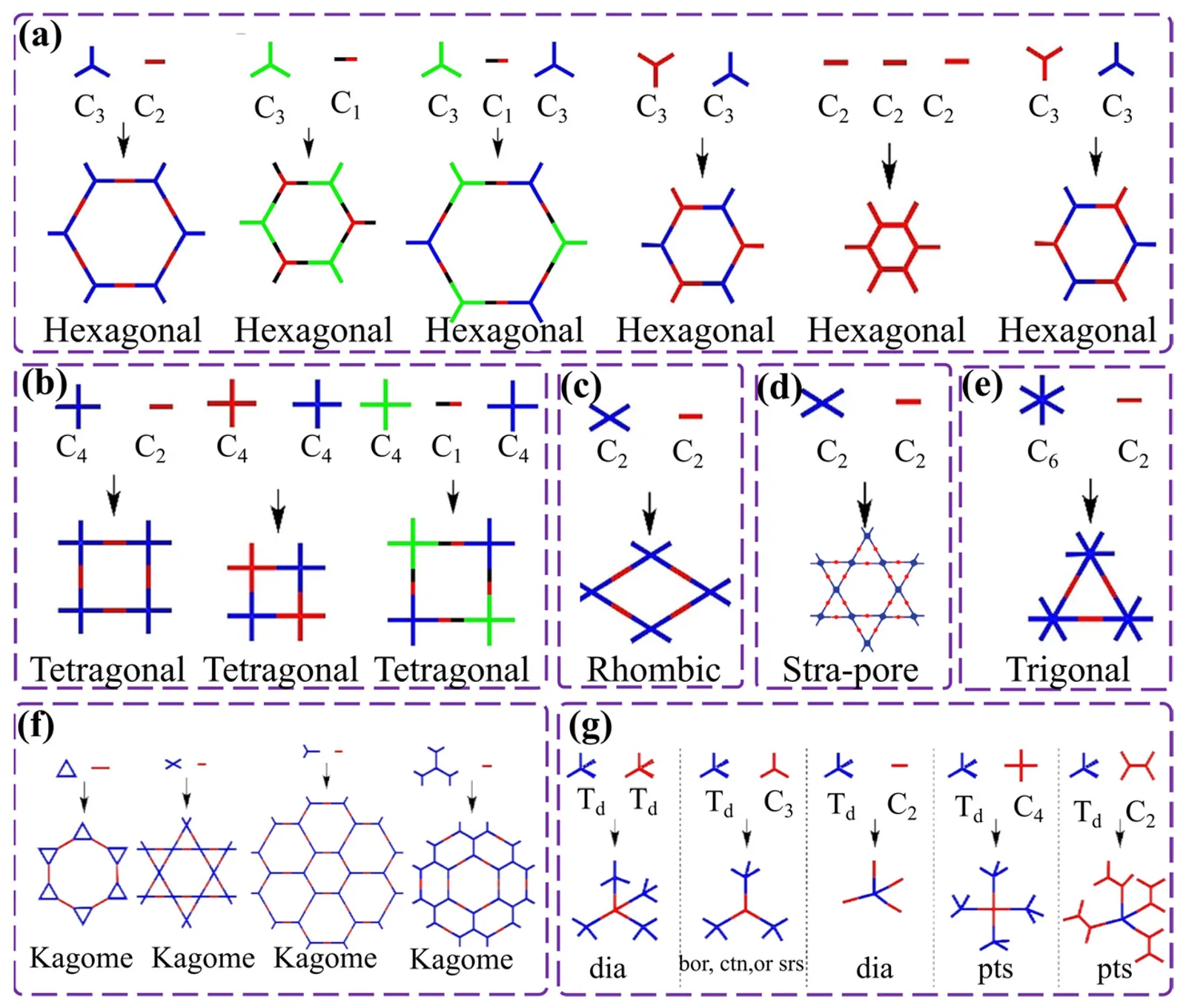
Topological structure diagram of various COFs. (**a**) Hexagonal COFs, (**b**) tetragonal COFs, (**c**) rhombic COFs, and (**d**) star-pore COFs. (**e**) trigonal COFs, (**f**) Kagome COFs, and (**g**) 3D COFs. Reprinted from [220]. © 2024 The Author(s). Published by the Royal Society of Chemistry. Reproduced by Open Access—Creative Commons CC BY 3.0.

Covalent organic frameworks (COFs) are crystalline porous organic materials constructed by linking organic building blocks through covalent bonds, forming highly ordered two- or three-dimensional networks. Typical examples include boronic acid trimerization, boronate ester formation, Schiff base reactions, and nitrile trimerization. Their structural diversity arises from the selection of building units, linkage chemistry and framework topology, which enable precise control over pore size, geometry and chemical functionality [44,181]. The spatial structure of COFs, which depends on the geometry of the building blocks and reaction conditions, is presented in Figure 22. Various topologies can be distinguished, including hexagonal (Figure 22a), tetragonal (Figure 22b), rhombic (Figure 22c), star-pore (Figure 22d), trigonal (Figure 22e), Kagome (Figure 22f), and three-dimensional (3D) structures (Figure 22g) [220].

Key features of COFs include high specific surface areas, low density, permanent porosity and tunable surface chemistry, alongside generally good thermal and chemical stability. Moreover, in comparison with MOFs, they exhibit improved biocompatibility and reduced toxicity due to the absence of toxic heavy metal ions [44]. These properties have led to their extensive investigation in gas adsorption and separation [221], photocatalysis [222], water treatment and separation membranes [223], energy storage and conversion [224], as well as anticorrosion prevention in coatings [225].

Given their structural tunability and functional versatility, covalent organic frameworks have recently emerged as promising functional additives for polyurethane matrices. In a recent study, Chen et al. incorporated silver nanoparticles (AgNPs) into two-dimensional COF nanosheets and dispersed the resulting hybrid in a waterborne elastic PU matrix. Under NIR irradiation at 500 mW cm^−2^, photothermal heating raised the film temperature to approximately 80 °C within 12 s. The damaged film achieved a reported healing efficiency above 98% after 4 min irradiation, while retaining high optical transmittance. Localized heating increases PU chain mobility and facilitates the rejoining of the damaged interface. The composite was proposed for transparent wearable coatings and goggles with defogging functionality [226].

A complementary approach was reported by Zhang et al., who incorporated a porphyrin-based biomass COF (Por-COF) into bio-based waterborne PU containing dynamic disulfide bonds. Under 730 nm irradiation at 2 W cm^−2^ for 10 min, the composite reached 79.3 °C and exhibited NIR-triggered healing efficiency above 68% (cut and healed at 60 °C for 12 h). Incorporation of Por-COF increased the tensile strength from 3.1 to 12.0 MPa, while antibacterial inhibition approached 100% against *Staphylococcus aureus* and *Escherichia coli*. To further demonstrate the sustainability of the developed bio-based material, the recyclability of composite films was demonstrated through dissolution in THF and subsequent reprocessing. The recovered material retained properties comparable to pristine polyurethane, highlighting its potential for sustainable antibacterial coating applications, including potential biomedical applications [227].

In another example, stretchable Sb^3+^-doped Cs_2_KTbCl_6_ lead-free double perovskite halide covalent organic frameworks were incorporated into polyurethane matrix containing both disulfide bonds and hydrogen bonds. These films exhibited excellent tensile strength above 31 MPa and fracture elongation of 987%. They also maintain stable photoluminescence intensity after 100 stretching cycles at 200% strain, demonstrating their potential for strain sensing and excellent tensile durability. Their original shape can be restored by heat treatment due to the synergistic effects of hydrogen and dynamic disulfide bonds, which facilitate molecular chain reorganization. The self-healing ability was evaluated by cutting the COF film into the letters “GXU” and removing the lower-right corner of the letter “G”. The damaged sample was then heated overnight, resulting in restoration of the original pattern. Unfortunately, the authors did not specify the exact conditions for self-treatment [45].

The selected examples discussed above are summarized in Table 5, which presents an overview of self-healing polyurethane systems incorporating different nanofillers and dynamic covalent and/or non-covalent interactions, together with their key characteristics and reported healing performance.

## 4. Challenges and Future Perspective

**Figure 23:**
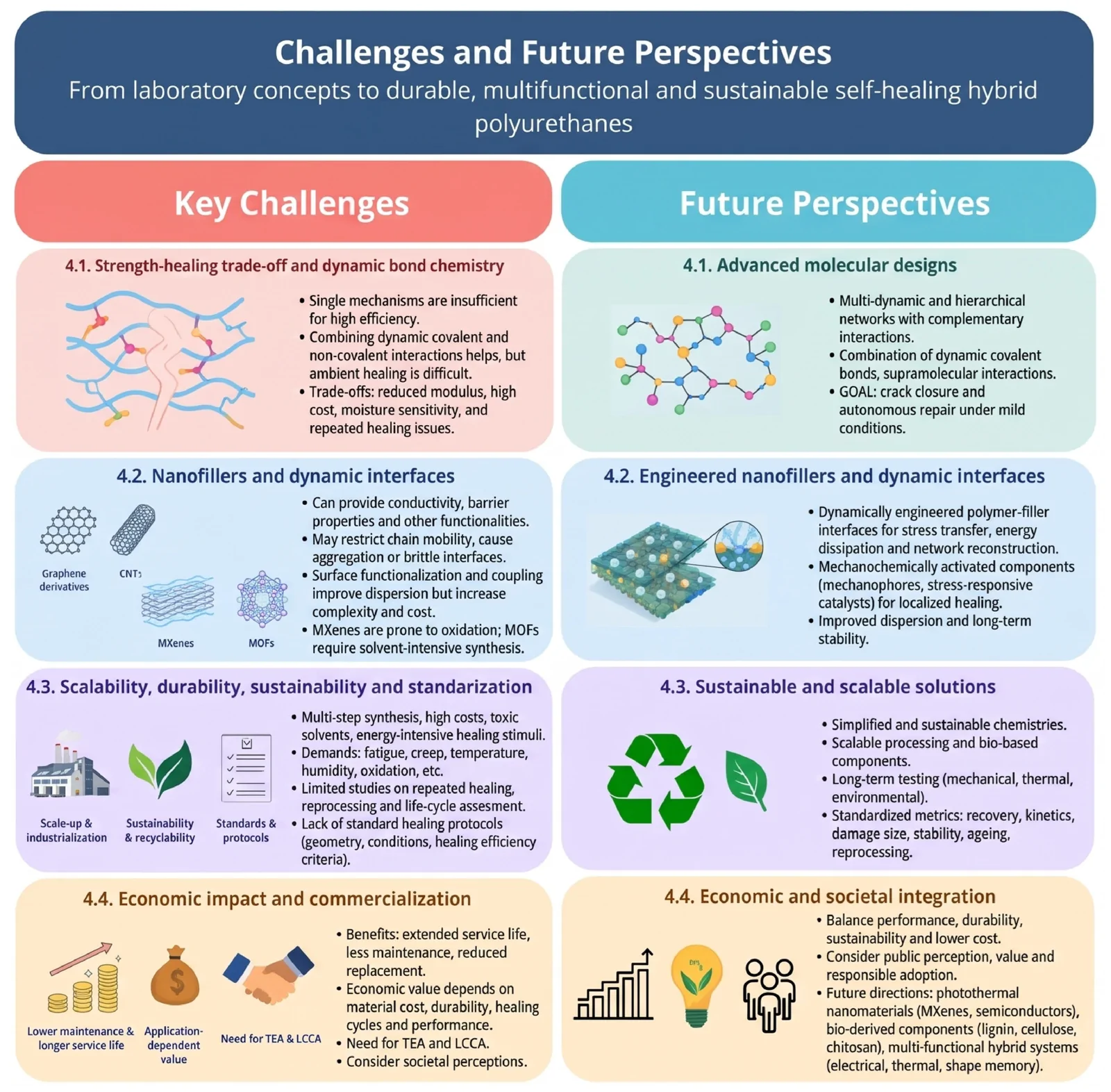
Graphical representation of challenges and future perspectives.

Self-healing hybrid polyurethanes now deliver room temperature healing efficiencies up to 95–100% while reaching tensile strengths of 14–72 MPa and toughness of >1300 MJ/m^3^ in optimized dual-network designs [228,229,230,231]. Despite these milestones, large gaps remain in scalability, durability under service conditions, and industrial implementation, especially for coatings, flexible electronics, and harsh environment applications. There are a few main points that must be taken into account [33,232]. A graphical representation of this description is shown in Figure 23.

### 4.1. Strength–Healing Trade-Off and Dynamic Bond Chemistry

The main limitation is the contradiction between mechanical strength and dynamic chain mobility required for healing. Reviews emphasize that single mechanisms cannot achieve high efficiency, motivating introducing dynamic covalent/supramolecular or multi-network designs [140]. Although combining dynamic covalent and non-covalent interactions can partially overcome this limitation, efficient recovery of macroscopic damage under ambient or mild conditions remains challenging [50]. The intrinsic stability of individual dynamic chemistries also remains an important limitation: aromatic disulfides may be associated with reduced modulus, high monomer cost, and synthetic complexity, while imine and boronic ester bonds can be sensitive to moisture and hydrolysis, and oxime–carbamate systems may exhibit reduced efficiency during repeated healing cycles [46,230,233]. Strong hydrogen-bonding or metal–ligand interactions can likewise restrict chain mobility when excessively concentrated, while repeated damage may induce creep or irreversible rearrangement [136,137,228]. Future molecular designs should focus on multi-dynamic and hierarchical networks in which complementary reversible interactions operate on different temporal and spatial scales. Promising approaches include combinations of multiple dynamic covalent bonds, hierarchical supramolecular interactions, and self-healing assisted by shape memory ability to facilitate crack closure and promote autonomous repair under mild conditions [234,235,236].

### 4.2. Nanofillers and Dynamic Interfaces

Functional nanofillers such as graphene derivatives, CNTs, MXenes, and MOFs can introduce conductivity, photothermal responsiveness, barrier properties, and other functionalities. However, they may simultaneously restrict polymer chain mobility, promote aggregation, or generate brittle interfaces. Surface functionalization and covalent or supramolecular coupling can improve dispersion and enable the polymer–filler interface to participate in reversible interactions, although these approaches often increase synthetic complexity and hinder scalability [140]. In addition, MXenes remain susceptible to oxidation, while large-scale processing and safe handling remain challenging. On the other hand, MOFs may suffer from solvent-intensive synthesis and aggregation during processing [237,238]. Future research should therefore move from passive filler incorporation toward dynamically engineered polymer–filler interfaces that contribute simultaneously to stress transfer, energy dissipation, and network reconstruction. Mechanochemically activated components, such as mechanophores or stress-responsive catalytic sites, may provide an additional route toward localized healing without continuous external stimuli.

### 4.3. Scalability, Durability, Sustainability, and Standardization

The transition from laboratory-scale to industrial implementation remains limited by multistep synthesis, high component costs, toxic solvents, energy-intensive healing stimuli, and the difficulty of achieving reproducible manufacturing [33,232]. These limitations are particularly relevant for coatings, flexible electronics, and harsh environment applications, where materials must withstand mechanical fatigue, creep, temperature fluctuations, humidity, oxidation, and other conditions [22,33,239]. Although recyclability and reprocessability are frequently reported for dynamic PUs, systematic studies of property retention after repeated healing and reprocessing, together with lifecycle and environmental assessments, remain limited [33,133,230]. A further obstacle is the lack of standardized healing protocols, as reported efficiencies depend strongly on damage geometry, healing conditions, strain rate, and recovery criteria, making direct comparison between systems difficult [240,241]. Future studies should therefore combine simplified and sustainable chemistries with scalable processing and long-term testing, while establishing standardized metrics for mechanical recovery, healing kinetics, damage size, cycling stability, ageing, and reprocessing. Such a comprehensive perspective would make the comparison between hybrid PU systems easier. It would also help to translate promising laboratory concepts into durable multifunctional materials for practical applications.

### 4.4. Economic Impact and Pathways to Commercialization and Future Directions

Self-healing hybrid polyurethanes may offer economic benefits by extending material service life, reducing maintenance frequency, and limiting the need for recoating or component replacement. These benefits could be particularly relevant to corrosion protection and components that are expensive or difficult to access for repair. However, the economic advantage remains application-dependent and should not be derived from healing efficiency alone: it also depends on the initial material cost, healing conditions, durability over repeated damage/healing cycles, and the extent to which the recovered properties meet service requirements [242,243]. Consequently, lifecycle cost assessments comparing self-healing systems with conventional PU alternatives are needed to establish whether higher upfront costs can be offset by reduced maintenance, downtime, and material consumption [244].

From an industrial perspective, the most promising strategies are likely to combine scalable processing with readily available or bio-based feedstocks, lower-cost dynamic chemistries, and healing mechanisms that operate under mild conditions. Hybrid fillers such as MXenes and metal–organic frameworks (MOFs) may provide valuable multifunctionality, but their costs, dispersion requirements, long-term stability, and potential effects on processing must also be considered [242,245,246]. Future studies should therefore integrate techno-economic assessment (TEA) and lifecycle cost assessments (LCCAs) with standardized mechanical-recovery and ageing tests. Reporting cost-related parameters alongside healing performance would help to identify which hybrid PU systems offer a realistic balance between performance, durability, sustainability, and commercial feasibility [244,246]. Beyond technical performance and economic feasibility, future research should also consider how societal perceptions of self-healing materials, including their perceived value, sustainability, and role in everyday life, can inform design strategies and guide their responsible development and adoption [247].

Looking ahead, self-healing hybrid PUs are expected to evolve from materials designed primarily to restore mechanical integrity into multifunctional, repairable and more sustainable polymer systems. Recent directions include incorporating bio-derived components such as lignin, cellulose, chitosan and vegetable-oil derivatives [248]; integrating photothermal nanomaterials, including MXenes [160] and semiconductor heterostructures [249], to activate healing on demand; and combining reversible networks with electrical, thermal or shape-memory functionality [231].

## 5. Conclusions

In conclusion, the development of self-healing hybrid polyurethanes depends on achieving an effective balance between mechanical performance, healing efficiency, durability, and processability. As discussed in this review, promising strategies include the design of multifunctional dynamic covalent and supramolecular networks, and the incorporation of functional modifiers such as nanomaterials. In particular, the combination of dynamic polymer networks with functional nanofillers offers opportunities to improve mechanical reinforcement, introduce additional functionalities, and enable externally triggered healing, although the resulting interfacial interactions and potential effects on chain mobility must be carefully controlled. Future research should focus on improving the stability and reproducibility of healing performance, clarifying the role of hybrid interfaces, and developing scalable and sustainable processing strategies. Addressing these challenges will be essential for translating laboratory advances into practical applications in protective coatings, flexible and wearable devices, and other advanced functional materials.

## Figures and Tables

**Table 1:** Studies on self-healing with dynamic covalent or non-covalent bonding in polyurethane network.

Dynamic Covalent or Non-Covalent Bonds	Tensile Strength (MPa)	Elongation at Break (%)	Healing Conditions	Max. Healing Efficiency * (%)	Ref.
Ditteluride bonds	17.5	860	NIR, 30 min	85.4% stress recovery/91.99% strain recovery	[60]
Diselenide bonds	6.5	468	70 °C, 2 h	60.6% stress-relaxation	[61]
Disulfide bonds	28.78	208.99	100 °C, 4 h	>99% of stress/strain recovery	[91]
Imine bonds	12.8	60	80 °C, 30 min	78.5	[96]
Boroxine bonds	1.63	1577.79	Water assisted (1 min), 60 °C, 20 min	92.02	[68]
Alkoxyamine bonds	<12	>250	80 °C under Ar, 2.5 h	70	[100]
Diels–Alder bonds	91.95	16.53	130 °C, 2 h	49.24	[103]
Hydrogen bonds	15.3	1132.8	100 °C, 4 h	83.6	[112]
Metal–ligand coordination bonds	1.4	2800	RT, 6 h	92	[116]
Host–guest interactions	<1.0	157	RT, 24 h/80 °C, 24 h	38/62	[118]
π-π stacking	>28	>750	50 °C, 12 h	~100	[123]
Ionic bonds	20.49	1007	60 °C, 1 h	97	[131]

* Values are reproduced from the studies cited in the text; missing data are not inferred. Because the measured properties and protocols differ, the entries should not be interpreted as a universal ranking.

**Table 2:** Mechanical performance and healing efficiency of self-healing polyurethanes over repeated healing cycles.

Healing Mechanisms	Healing Conditions ^a^	Stress (MPa)			Strain (%)			Healing Efficiency ^b^ (%)		Ref.
		Pristine ^c^	1st Healed	3rd Healed	Pristine ^c^	1st Healed	3rd Healed	1st Healed	3rd Healed	
Diels–Alder and imine bonds	120 °C, 120 min	~27.5	25	21	>500	~480	~420	94.3	79.2	[145]
Metal–ligand coordination bonds	simulated sunlight exposure (50 W xenon lamp), 30 min	~28	~27.5	~26.5	~1770	~1750	~1630	98.6	95.5	[146]
Disulfide and imine bonds	70 °C, 150 min	~13	<12	<10	~625	~580	~440	92.5	73.4	[147]
Disulfide bonds	NIR, 15 min	~35	~33	~32.5	~880	~810	~800	91	~90	[148]
Diels–Alder bonds	120 °C for 15 min followed by 60 °C for 24 h	6.55	5.23	3.78	444	304	283	71	38	[149]
Diels–Alder and disulfide bonds	120 °C for 10 min followed by 60 °C for 24 h	~10	8.5	7	~650	<600	400	90.6	71	[150]
Diels–Alder bonds	130 °C for 15 min followed by 60 °C for 12 h	~14	13.5	10	~285	~243	~160	96.4	71.4	[151]

^a^ All of the samples were scratched to the depth of 0.5 mm; ^b^ mechanical recovery; ^c^ without scratch.

**Table 3:** Reprocessing and recyclability of self-healing polyurethane materials.

Intrinsic Self-Healing Mechanisms	Reprocessing Method and Conditions	Mechanical Performance After Reprocessing		Self-Healing After Reprocessing	Ref.
		Stress (MPa) Pristine ^a^/3rd Cycle ^b^	Strain (%) Pristine ^a^/3rd Cycle ^b^		
Hydrogen bonds and disulfide bonds	Hot pressing: 100 °C, 5 MPa, 30 min; also DMAc at 80 °C	50 ^a^/45 ^b^	~820 ^a^/~810 ^b^	Not reported	[153]
Hydrogen bonds	Hot pressing: 105 °C, 10 MPa, 10 min	~30 ^a^/~26 ^b^	~1000 ^a^/~980 ^b^	92.1%, 88.3%, 80.5% of tensile strength over three successive damage-repair cycles	[154]
Disulfide bonds and imine bonds	Hot pressing: 140 °C, 20 MPa, 10 min	3.22 ^a^/4.34 ^b^	234 ^a^/121 ^b^	Not reported	[155]
Imine bonds	Hot pressing: 140 °C, 20 MPa, 10 min	2.70 ^a^/2.02 ^b^	~275 ^a^/~140 ^b^	Not reported	[155]
Hydrogen bonds	Solvent-assisted recycling in DMF at 110 °C for 20–30 min	62 ^a^/44 ^b^	~700 ^a^/564 ^b^	Not reported	[156]

^a^ pristine elastomer before recycling; ^b^ elastomer after the third recycling cycle.

**Table 4:** Studies on self-healing based on multiple bond interactions in polyurethane networks.

Dynamic Covalent Bond	Non-Covalent Bond	Tensile Strength (MPa)	Elongation at Break (%)	Healing Conditions	Max. Healing Efficiency * (%)	Ref.
Disulfide bonds	Hydrogen bonds	3.35	334	Full crack closure at 60 °C, 15 min	-	[165]
Disulfide bonds	Hydrogen bonds	<1	1100	−10 °C, 12 h	93	[166]
Disulfide bonds	Hydrogen bonds	30	850	RT, 48 h	35	[162]
Disulfide bonds	Hydrogen bonds	11.8	2423	80 °C, 8 h	93	[169]
	Metal–ligand coordination bonds					
Disulfide bonds	Metal–ligand coordination bonds	23.9	~1000	70 °C, 24 h	85.5	[170]
Disulfide bonds	-	25.5	<400	100 °C, 3 h	82	[173]
Diels–Alder bonds						
Disulfide bonds	-	21.1	755.17	120 °C for 30 min followed by 70 °C for 24 h	90.38	[174]
Diels–Alder bonds						
Disulfide bonds	-	12.95	623	80 °C, 150 min	95.2	[147]
Imine bonds						
Disulfide bonds	Hydrogen bonds	<2.50	~1600	60 °C, 48 h	90	[176]
	Metal–ligand coordination bonds					
-	Hydrogen bonds	16.14	532	30 °C, 50 h	94.8	[177]
	π-π interactions					
-	Metal–ligand coordination bonds	20.09	1040.65	90 °C, 25 h	89.49	[178]
	Hydrogen bonds					
Diels–Alder bonds	Hydrogen bonds	~1	300	60 °C, 12 h	95	[179]
Disulfide bonds						

* Values are reproduced from the studies cited in the text; missing data are not inferred. Because the measured properties and protocols differ, the entries should not be interpreted as a universal ranking.

**Table 5:** Studies on self-healing with nanofillers and dynamic covalent or/and non-covalent bonding in polyurethane network.

Dynamic Covalent or/and Non-Covalent Bond	Nanofiller	Tensile Strength (MPa)	Elongation at Break (%)	Healing Conditions	Max. Healing Efficiency * (%)	Ref.
Hydrogen bonds	Graphene	5	<2500	80 °C, 1 h	88	[183]
Hydrogen bonds	Graphene	19.73	361.76	80 °C, 24 h; solvent assisted	<80	[184]
Disulfide bonds and hydrogen bonds	Graphene oxide	78	1273	25 °C, 24 h	89	[39]
Hydrogen bonds and metal–ligand coordination bonds	Graphene oxide	18.65	>1000	90 °C, 15 h	92	[187]
Hydrogen bonds	Reduced graphene oxide	-	-	NIR, 10 min	80.29	[189]
Disulfide bonds	Carbon nanotubes	8	-	60 °C, 16 min; or UV for 48 h	88.4	[193]
Hydrogen bonds	Carbon nanotubes	17.5	420	80 °C, 24 h, water assisted	78	[194]
Disulfide bonds and hydrogen bonds	Carbon nanotubes	22.2	>1000	80 °C, 4 h	90	[195]
Hydrogen bonds	MXene (Ti_3_C2Tx)	29.4	1009.3	NIR, 5 min	95	[206]
Hydrogen bonds	Mxene (Ti_3_C2Tx)	58.8	790	NIR, 30 min	82.7	[207]
Hydrogen bonds	Mxene	1.65	571	IR, 15 min	81.3	[208]
Disulfide bonds	MXene and carbon nanotubes	8.82	809.1	RT, 2 h	Crack closure	[209]
Diels–Alder bonds and hydrogen bonds	MOF (alkyl-UiO-66)	<30	818	80 °C, <1 min	Notch closure	[214]
Hydrogen bonds	MOF (ZIF-7-UPy)	48	1200	RT, 24 h	87	[218]
Disulfide bonds	MOF (UiO-66-NH_2_)	5.16	1117.1	110 °C, 24 h	96.1	[219]
-	*N*,*N*,*N*′,*N*′-tetrakis(4-aminopheneyl)-1,4-phenylenediamine (TAPPDA) and *N*,*N*,*N*′,*N*′-tetrakis(4-formylpheneyl)-1,4-phenylenediamine (TFPPDA) as COF nanosheets and plasmonic silver nanoparticle	15.47	552.81	NIR, 4 min	98.39	[226]
Disulfide bonds	Porphyrin-based biomass COF	12	2722	60 °C, 12 h	68	[227]
Disulfide bonds and hydrogen bonds	Sb^3+^-doped Cs_2_KTbCl_6_ lead-free double perovskite halide	31	987	-	-	[45]

* Values are reproduced from the studies cited in the text; missing data are not inferred. Because the measured properties and protocols differ, the entries should not be interpreted as a universal ranking.

## Data Availability

No new data were created or analyzed in this study. Data sharing is not applicable to this article.

## References

[1] Singh N., Walker T.R. (2024). Plastic Recycling: A Panacea or Environmental Pollution Problem. npj Mater. Sustain..

[2] OECD Global Plastics Outlook: Economic Drivers, Environmental Impacts and Policy Options. https://www.oecd.org/en/publications/global-plastics-outlook_de747aef-en.html.

[3] Full Report: Global Plastics Outlook. https://www.oecd.org/en/publications/global-plastics-outlook_aa1edf33-en/full-report.html.

[4] Fleck A. Infographic: Recycling Efforts Not Enough to Solve Plastic Waste Problem. https://www.statista.com/chart/27756/global-waste-management-projections.

[5] Agostinho B., Silvestre A.J.D., Coutinho J.A.P., Sousa A.F. (2023). Synthetic (Bio)Degradable Polymers—When Does Recycling Fail?. Green. Chem..

[6] Kim M.S., Chang H., Zheng L., Yan Q., Pfleger B.F., Klier J., Nelson K., Majumder E.L.-W., Huber G.W. (2023). A Review of Biodegradable Plastics: Chemistry, Applications, Properties, and Future Research Needs. Chem. Rev..

[7] Briassoulis D., Pikasi A., Hiskakis M., Arias A., Moreira M.T., Ioannidou S.M., Ladakis D., Koutinas A. (2023). Life-Cycle Sustainability Assessment for the Production of Bio-Based Polymers and Their Post-Consumer Materials Recirculation through Industrial Symbiosis. Curr. Opin. Green. Sustain. Chem..

[8] Joseph T.M., Unni A.B., Joshy K.S., Kar Mahapatra D., Haponiuk J., Thomas S. (2023). Emerging Bio-Based Polymers from Lab to Market: Current Strategies, Market Dynamics and Research Trends. C.

[9] Marturano V., Marotta A., Salazar S.A., Ambrogi V., Cerruti P. (2023). Recent Advances in Bio-Based Functional Additives for Polymers. Prog. Mater. Sci..

[10] Schade A., Melzer M., Zimmermann S., Schwarz T., Stoewe K., Kuhn H. (2024). Plastic Waste Recycling—A Chemical Recycling Perspective. ACS Sustain. Chem. Eng..

[11] Liu L., Barlaz M.A., Johnson J.X. (2024). Economic and Environmental Comparison of Emerging Plastic Waste Management Technologies. Resour. Conserv. Recycl..

[12] Suzuki G., Uchida N., Tanaka K., Higashi O., Takahashi Y., Kuramochi H., Yamaguchi N., Osako M. (2024). Global Discharge of Microplastics from Mechanical Recycling of Plastic Waste. Environ. Pollut..

[13] Belyamani I., Bourdon S., Brossard J.-M., Cauret L., Fontaine L., Montembault V., Maris J. (2024). A Sustainable Approach toward Mechanical Recycling Unsortable Post-Consumer WEEE: Reactive and Non-Reactive Compatibilization. Waste Manag..

[14] Martínez-Narro G., Hassan S., Phan A.N. (2024). Chemical Recycling of Plastic Waste for Sustainable Polymer Manufacturing—A Critical Review. J. Environ. Chem. Eng..

[15] Plastic Market Size, Share and Trends Report, 2026–2033. https://www.grandviewresearch.com/industry-analysis/global-plastics-market.

[16] Zechel S., Hager M.D., Priemel T., Harrington M.J. (2019). Healing through Histidine: Bioinspired Pathways to Self-Healing Polymers via Imidazole–Metal Coordination. Biomimetics.

[17] IOS Press Ebooks—Self Healing Materials—Pioneering Research in The Netherlands. https://ebooks.iospress.nl/book/self-healing-materials-pioneering-research-in-the-netherlands.

[18] Utrera-Barrios S., Verdejo R., López-Manchado M.A., Hernández Santana M. (2020). Evolution of Self-Healing Elastomers, from Extrinsic to Combined Intrinsic Mechanisms: A Review. Mater. Horiz..

[19] Gai Y., Li H., Li Z. (2021). Self-Healing Functional Electronic Devices. Small.

[20] Mashkoor F., Lee S.J., Yi H., Noh S.M., Jeong C. (2022). Self-Healing Materials for Electronics Applications. Int. J. Mol. Sci..

[21] Blaiszik B.J., Kramer S.L.B., Olugebefola S.C., Moore J.S., Sottos N.R., White S.R. (2010). Self-Healing Polymers and Composites. Annu. Rev. Mater. Res..

[22] Chen B., Zhang Y., Mao C., Gan Y., Li B., Cai H. (2022). Research on Electrospinning Thermosetting-Thermoplastic Core-Shell Nanofiber for Rapid Self-Healing of Carbon Fiber/Epoxy Composites. Compos. Sci. Technol..

[23] Xu Y., Shen R., Tang J., Zou X., Wan W., Guo H. (2024). Optimizing Mechanical Properties and Corrosion Resistance in Core-Shell Nanofiber Epoxy Self-Healing Coatings: Impact of Shell Material Variation. Polym. Eng. Sci..

[24] Chung U.S., Min J.H., Lee P.-C., Koh W.-G. (2017). Polyurethane Matrix Incorporating PDMS-Based Self-Healing Microcapsules with Enhanced Mechanical and Thermal Stability. Colloids Surf. A Physicochem. Eng. Asp..

[25] Lee M.W., An S., Kim Y.-I., Yoon S.S., Yarin A.L. (2018). Self-Healing Three-Dimensional Bulk Materials Based on Core-Shell Nanofibers. Chem. Eng. J..

[26] Li C., Guo H., Wu Z., Wang P., Zhang D., Sun Y. (2023). Self-Healable Triboelectric Nanogenerators: Marriage between Self-Healing Polymer Chemistry and Triboelectric Devices. Adv. Funct. Mater..

[27] Wan X., Mu T., Yin G. (2023). Intrinsic Self-Healing Chemistry for Next-Generation Flexible Energy Storage Devices. Nano-Micro Lett..

[28] Li Y., Jin Y., Zeng W., Jin H., Shang X., Zhou R. (2023). Bioinspired Fast Room-Temperature Self-Healing, Robust, Adhesive, and AIE Fluorescent Waterborne Polyurethane via Hierarchical Hydrogen Bonds and Use as a Strain Sensor. ACS Appl. Mater. Interfaces.

[29] Zhang Z., Qiu J., Sha Y., He Y., Ma X., Li G., Yang R., Wang R., Zhang M., Luo Y. (2024). Molecularly Engineered Tough and Room-Temperature Self-Healing Polyurethanes for Resistive Strain Sensors. ACS Appl. Polym. Mater..

[30] Duprey C., Ajeev A., Hong D., Webb K., Veres S., Chen G., Linn E., Lusvardi G., Liu Z., Wang R. (2025). A Recyclable PANI/PAAMPSA Nanocomposite with Repeatable, Rapid, Autonomous Self-Healing, and Unprecedented Electro-Mechanical Properties. Adv. Compos. Hybrid Mater..

[31] Liu Y., Xu B., Xie Z., Yang J., Liu Y., Yang Y., Xu H. (2023). Intrinsically Stretchable, Self-Healing, and Large-Scale Epidermal Bioelectrode Arrays for Electrophysiology and Gesture Recognition. ACS Appl. Mater. Interfaces.

[32] Yimyai T., Crespy D., Rohwerder M. (2023). Corrosion-Responsive Self-Healing Coatings. Adv. Mater..

[33] Willocq B., Odent J., Dubois P., Raquez J.-M. (2020). Advances in Intrinsic Self-Healing Polyurethanes and Related Composites. RSC Adv..

[34] Xie J., Yang M., Liang J., Hu J., Li Q., He J. (2021). Self-Healing of Internal Damage in Mechanically Robust Polymers Utilizing a Reversibly Convertible Molecular Network. J. Mater. Chem. A.

[35] Wang J., Tang J., Chen D., Xing S., Liu X., Hao J. (2023). Intrinsic and Extrinsic Self-Healing Fiber-Reinforced Polymer Composites: A Review. Polym. Compos..

[36] Tan T., Siew W.H., Han L., Given M., McKendry C., Liggat J., Li Q., He J. (2023). Self-Healing of Electrical Damage in Microphase-Separated Polyurethane Elastomers with Robust Dielectric Strength Utilizing Dynamic Hydrogen Bonding Networks. ACS Appl. Polym. Mater..

[37] Somarathna H.M.C.C., Raman S.N., Mohotti D., Mutalib A.A., Badri K.H. (2018). The Use of Polyurethane for Structural and Infrastructural Engineering Applications: A State-of-the-Art Review. Constr. Build. Mater..

[38] Hofmann T., Ghoshal S., Tufenkji N., Adamowski J.F., Bayen S., Chen Q., Demokritou P., Flury M., Hüffer T., Ivleva N.P. (2023). Plastics Can Be Used More Sustainably in Agriculture. Commun. Earth Environ..

[39] Zhu X., Zhang W., Lu G., Zhao H., Wang L. (2022). Ultrahigh Mechanical Strength and Robust Room-Temperature Self-Healing Properties of a Polyurethane–Graphene Oxide Network Resulting from Multiple Dynamic Bonds. ACS Nano.

[40] Yelwa J.M., Musa H., Fasanya O.O., Drammeh A., Yahaya J.Y., Abdulkadir J., Mohammed M., Yamani A.M., Aliyu B.M., Khanna V. (2026). Carbon-Based Nanocomposites for Self-Healing Materials. Carbon-Based Nanocomposites for Sustainable Applications, Volume III: Biomedicine, Electronics, and Catalysis.

[41] Rajeev S., Amayeka S.A., Panicker U.G. (2026). Self-Healing and Sense-Healing: Metal–Organic Framework-Filled Polymers for Robust and Durable Soft Robotics. Mater. Horiz..

[42] Haswani S., Kandasubramanian B. (2026). Symphony of Strength-Polyurethane-Infused MXene/Graphene Composites for Self-Healing Smart Material. Polym.-Plast. Technol. Mater..

[43] Zhao B., He C., Sun Z., Guan R., Zheng H., He Y., Tao L., Wang X., Yin J. (2026). Confinement-Locked Dual Dynamic Networks Enable Waterborne Polyurethane for Multifunctional Wearable Electronic Leather. Chem. Eng. J..

[44] Qin Y., Zhu X., Huang R. (2023). Covalent Organic Frameworks: Linkage Types, Synthetic Methods and Bio-Related Applications. Biomater. Sci..

[45] Xie S., Dong Y., Wang C., Li W., Hu H., Xue J., Li Z., Gao W., Zou B. (2025). In Situ Fabricated Self-Healing, Highly Stretchable Sb3+-Doped Non-Lead Double Perovskite Halide COFs Exhibits Narrow-Band Emission Properties. Adv. Mater. Technol..

[46] An Z.-W., Xue R., Ye K., Zhao H., Liu Y., Li P., Chen Z.-M., Huang C.-X., Hu G.-H. (2023). Recent Advances in Self-Healing Polyurethane Based on Dynamic Covalent Bonds Combined with Other Self-Healing Methods. Nanoscale.

[47] Lee W.-J., Oh H.-G., Cha S.-H. (2021). A Brief Review of Self-Healing Polyurethane Based on Dynamic Chemistry. Macromol. Res..

[48] Aguirresarobe R.H., Nevejans S., Reck B., Irusta L., Sardon H., Asua J.M., Ballard N. (2021). Healable and Self-Healing Polyurethanes Using Dynamic Chemistry. Prog. Polym. Sci..

[49] Menon A.V., Madras G., Bose S. (2019). The Journey of Self-Healing and Shape Memory Polyurethanes from Bench to Translational Research. Polym. Chem..

[50] Zhao J., Zhu J., Zhang J., Huang Z., Qi D. (2024). Review of Research on Thermoplastic Self-Healing Polyurethanes. React. Funct. Polym..

[51] Luo Y.-L., Gao W.-T., Luo Z.-Y., Li C.-H. (2024). Toward Mechanically Robust Self-Healing Polyurethanes Using Dynamics Chemistry. Mater. Chem. Front..

[52] Li Y., Jin Y., Fan W., Zhou R. (2022). A Review on Room-Temperature Self-Healing Polyurethane: Synthesis, Self-Healing Mechanism and Application. J. Leather Sci. Eng..

[53] Healing Efficiency—An Overview|ScienceDirect Topics. https://www.sciencedirect.com/topics/engineering/healing-efficiency.

[54] Wu D.Y., Meure S., Solomon D. (2008). Self-Healing Polymeric Materials: A Review of Recent Developments. Prog. Polym. Sci..

[55] Panahi P., Khorasani S.N., Mensah R.A., Das O., Neisiany R.E. (2024). A Review of the Characterization Methods for Self-Healing Assessment in Polymeric Coatings. Prog. Org. Coat..

[56] Bekas D.G., Tsirka K., Baltzis D., Paipetis A.S. (2016). Self-Healing Materials: A Review of Advances in Materials, Evaluation, Characterization and Monitoring Techniques. Compos. Part B Eng..

[57] Choufi N.E., Mustapha S., B A.T., Grady B.P. (2022). An Overview of Self-Healable Polymers and Recent Advances in the Field. Macromol. Rapid Commun..

[58] Ma Z., Li H., Jing X., Liu Y., Mi H.-Y. (2021). Recent Advancements in Self-Healing Composite Elastomers for Flexible Strain Sensors: Materials, Healing Systems, and Features. Sens. Actuators A Phys..

[59] Zeng W., Jin Y., Zhou R., Li Y., Chen H. (2024). Double Crosslinked Networks Waterborne Polyurethane with Self-Healing, Recyclable and Antibacterial Functions Based on Dynamic Bonds and Used for Temperature/Light Sensor. Chem. Eng. J..

[60] Zeng W., Jin Y., Li Y., Zhou R., Shi L., Bai L., Shang X., Li J. (2023). Ambient Temperature Self-Healing Waterborne Polyurethane Based on Dynamic Ditelluride Bonds with Recyclable and Antibacterial Functions. Prog. Org. Coat..

[61] Liu R., Li S., Yao N., Xia J., Li M., Ding H., Xu L., Yang X. (2022). Castor Oil-Based Polyurethane Networks Containing Diselenide Bonds: Self-Healing, Shape Memory, and High Flexibility. Prog. Org. Coat..

[62] Du W., Jin Y., Pan J., Fan W., Lai S., Sun X. (2018). Thermal Induced Shape-Memory and Self-Healing of Segmented Polyurethane Containing Diselenide Bonds. J. Appl. Polym. Sci..

[63] Ye G., Jiang T. (2021). Preparation and Properties of Self-Healing Waterborne Polyurethane Based on Dynamic Disulfide Bond. Polymers.

[64] Zhang M., Zhao F., Luo Y. (2019). Self-Healing Mechanism of Microcracks on Waterborne Polyurethane with Tunable Disulfide Bond Contents. ACS Omega.

[65] Xu X., Ma X., Cui M., Zhao H., Stott N.E., Zhu J., Yan N., Chen J. (2024). Fully Biomass-Derived Polyurethane Based on Dynamic Imine with Self-Healing, Rapid Degradability, and Editable Shape Memory Capabilities. Chem. Eng. J..

[66] Sun Y., Sheng D., Wu H., Tian X., Xie H., Shi B., Liu X., Yang Y. (2021). Bio-Based Vitrimer-like Polyurethane Based on Dynamic Imine Bond with High-Strength, Reprocessability, Rapid-Degradability and Antibacterial Ability. Polymer.

[67] Bao C., Miao R., Yin Y., Xu Y., Ding Y., Liu J., Xin Z. (2024). Mechanically Robust yet Body-Temperature Self-Healable Polyurethane Elastomer via the Cross-Linking of Dynamic Boroxines. ACS Appl. Polym. Mater..

[68] Longfang R., Congcong L., Taotao Q. (2022). Preparation of Boroxine-Based Self-Healing Polyurethane with Repeatable Adhesive Property under Mild Conditions. Mater. Today Chem..

[69] Jia Y., Delaittre G., Tsotsalas M. (2022). Covalent Adaptable Networks Based on Dynamic Alkoxyamine Bonds. Macromol. Mater. Eng..

[70] Elizalde F., Pertici V., Aguirresarobe R., Ximenis M., Vozzolo G., Lezama L., Ruipérez F., Gigmes D., Sardon H. (2024). Tuning Reprocessing Temperature of Aliphatic Polyurethane Networks by Alkoxyamine Selection. ACS Appl. Polym. Mater..

[71] Yu C., Wang Z., Fei G., Zhang X., de Luna M.S., Lavorgna M., Xia H. (2024). Robust Self-Healing Waterborne Polyurethane Coatings via Dynamic Covalent Diels-Alder Bonds for Corrosion Protection. J. Polym. Sci..

[72] Fortunato G., van den Tempel P., Bose R.K. (2024). Advances in Self-Healing Coatings Based on Diels-Alder Chemistry. Polymer.

[73] Yang T., Lu X., Wang X., Li Y., Wei X., Wang W., Sun J. (2023). Healable, Recyclable, and Scratch-Resistant Polyurethane Elastomers Cross-Linked with Multiple Hydrogen Bonds. ACS Appl. Polym. Mater..

[74] Gao Y., Wu Y., Zhao J., Ma Y., Liu Y., Gao C. (2022). Self-Assembly Strategy Based on Multiple Hydrogen Bonds for Super Tough, Self-Healing Polyurethane Elastomers. Polymer.

[75] Pan G.-F., Wang Z., Gong X.-B., Wang Y.-F., Ge X., Xing R.-G. (2022). Self-Healable Recyclable Thermoplastic Polyurethane Elastomers: Enabled by Metal–Ligand Bonds between the Cerium(III) Triflate and Phloretin. Chem. Eng. J..

[76] Li M.X., Rong M.Z., Zhang M.Q. (2021). Enhancement of Mechanochemical Self-Blocking and Self-Healing of Early Minor Damages in Polymers through Hyperbranched Architecture. Express Polym. Lett..

[77] Tian C., Ning J., Yang Y., Zeng F., Huang L., Liu Q., Lv J., Zhao F., Kong W., Cai X. (2022). Super Tough and Stable Solid–Solid Phase Change Material Based on π-π Stacking. Chem. Eng. J..

[78] Mao H., Zhang Q., Lin L., He X., Wang L. (2023). A Self-Healable and Recyclable Zwitterionic Polyurethane Based on Dynamic Ionic Interactions. Polymers.

[79] Singh N.S.S., Fizuli M.G., Khazal H., Kumar S., Sharma R., Moradi P. (2026). Supramolecular Chemistry in Polyurethane for Self-Healing: Design, Challenges, and Future Insights. React. Funct. Polym..

[80] Li B., Cao P.-F., Saito T., Sokolov A.P. (2023). Intrinsically Self-Healing Polymers: From Mechanistic Insight to Current Challenges. Chem. Rev..

[81] Shahi S., Roghani-Mamaqani H., Hoogenboom R., Talebi S., Mardani H. (2022). Stimuli-Responsive Covalent Adaptable Hydrogels Based on Homolytic Bond Dissociation and Chain Transfer Reactions. Chem. Mater..

[82] Fan W., Jin Y., Shi L., Du W., Zhou R., Lai S., Shen Y., Li Y. (2020). Achieving Fast Self-Healing and Reprocessing of Supertough Water-Dispersed “Living” Supramolecular Polymers Containing Dynamic Ditelluride Bonds under Visible Light. ACS Appl. Mater. Interfaces.

[83] Ji S., Cao W., Yu Y., Xu H. (2014). Dynamic Diselenide Bonds: Exchange Reaction Induced by Visible Light without Catalysis. Angew. Chem. Int. Ed..

[84] Ji S., Fan F., Sun C., Yu Y., Xu H. (2017). Visible Light-Induced Plasticity of Shape Memory Polymers. ACS Appl. Mater. Interfaces.

[85] Cao Z., Huang L., Xu S., Gao X., Zhou B. (2025). Introducing Dynamic Disulfide Bonds: A New Strategy for Enhancing the Comprehensive Performance of High Content Polyurethane Modified Asphalt. Constr. Build. Mater..

[86] Xi J., Wang N., Wei L. (2025). Design and Preparation of a High Mechanical Strength Recyclable Polyurethane Adhesive Based on Dynamic Disulfide Bonds. J. Polym. Sci..

[87] Li Y., Zhou M., Xia L., Yang X., Shi Y. (2024). Room-Temperature Self-Healing Polyurethane Elastomers with High Strength and Superior Self-Healing Efficiency Based on Aromatic Disulfide-Induced. Colloids Surf. A Physicochem. Eng. Asp..

[88] Xia W., Sun B., He Y., Li Y., Xia T., Yang Y., Yang C. (2024). Effect of Disulfide Bond Content on Thermal-Healing Properties of Castor Oil-Based Polyurethanes. Polym. Degrad. Stab..

[89] Otsuka H., Nagano S., Kobashi Y., Maeda T., Takahara A. (2010). A Dynamic Covalent Polymer Driven by Disulfide Metathesis under Photoirradiation. Chem. Commun..

[90] Kim S.-M., Jeon H., Shin S.-H., Park S.-A., Jegal J., Hwang S.Y., Oh D.X., Park J. (2018). Superior Toughness and Fast Self-Healing at Room Temperature Engineered by Transparent Elastomers. Adv. Mater..

[91] Xian C., Deng T., Sun B., Yang W., Li B., Li Y., Xia T., Yang C., Yang Y. (2025). Castor Oil-Segmented Polyurethanes with Disulfide Bonds: Excellent Self-Healing and Mechanical Properties. J. Polym. Res..

[92] Zhang M., He B., Yang B., Yang Z., Sui H., Chen H., Duan J., Tang Q. (2025). In Situ Polymerization of Disulfide Bonds Based Polyurethane for Wide-Temperature Self-Healing Perovskite Solar Cells. Chem. Eng. J..

[93] Chao A., Negulescu I., Zhang D. (2016). Dynamic Covalent Polymer Networks Based on Degenerative Imine Bond Exchange: Tuning the Malleability and Self-Healing Properties by Solvent. Macromolecules.

[94] Hu J., Mo R., Sheng X., Zhang X. (2020). A Self-Healing Polyurethane Elastomer with Excellent Mechanical Properties Based on Phase-Locked Dynamic Imine Bonds. Polym. Chem..

[95] Tang X., Huang Y., Guo X., Xu C.-A., Yuan T. (2025). Light Cured Bio-Based Self-Healing Polyurethane Coating Based on Imine Bond. J. Appl. Polym. Sci..

[96] Banan A.R., Keshavarz S.M. (2025). Biobased Self Healing Waterborne Polyurethane with Vanillin Derived Dynamic Imine Bonds for Enhanced Mechanical Strength and Performance. Sci. Rep..

[97] Mao H., Zhang Y., Zhao L., Li H., Wu H., Lin L. (2025). Self-Healing and Recyclable Fabric Coatings Based on Boroxine–Polyurethane. ACS Appl. Polym. Mater..

[98] Fischer H. (2001). The Persistent Radical Effect: A Principle for Selective Radical Reactions and Living Radical Polymerizations. Chem. Rev..

[99] Gaudel-Siri A., Siri D., Tordo P. (2006). Homolysis of N-Alkoxyamines: A Computational Study. ChemPhysChem.

[100] Yuan C., Rong M.Z., Zhang M.Q. (2014). Self-Healing Polyurethane Elastomer with Thermally Reversible Alkoxyamines as Crosslinkages. Polymer.

[101] Sattar M.A., Patnaik A. (2020). Design Principles of Interfacial Dynamic Bonds in Self-Healing Materials: What Are the Parameters?. Chem.—Asian J..

[102] Cheng M., Fu Q., Tan B., Ma Y., Fang L., Lu C., Xu Z. (2022). Build a Bridge from Polymeric Structure Design to Engineering Application of Self-Healing Coatings: A Review. Prog. Org. Coat..

[103] Xi J., Wang N. (2024). Synthesis of High Mechanical Strength and Thermally Recyclable and Reversible Polyurethane Adhesive by Diels–Alder Reaction. Macromol. Chem. Phys..

[104] Du X., Tong Y., Wang T., Zhang A., Xu Q. (2025). Self-Healing and Recyclable Polyurethane-Based Solid-State Polymer Electrolyte via Diels-Alder Dynamic Network. J. Mol. Struct..

[105] Srivastav R.S., More A.P. (2025). A Comprehensive Review of Self-Healing Polymers: Mechanisms, Types, and Industry Implications. Polym. Adv. Technol..

[106] Xu H., Fan Z., Li H., Zhang X., Deng C., Guo X. (2025). Intrinsic Self-Healing Polymers: Research Progress, Application Direction, and Prospects. Polym. -Plast. Technol. Mater..

[107] MacLeod J.M., Rosei F., Andrews D.L., Scholes G.D., Wiederrecht G.P. (2011). 3.02—Directed Assembly of Nanostructures. Comprehensive Nanoscience and Technology.

[108] Xie Z., Hu B.-L., Li R.-W., Zhang Q. (2021). Hydrogen Bonding in Self-Healing Elastomers. ACS Omega.

[109] Liu L., Li X., Ren X., Wu G. (2020). feng Flexible Strain Sensors with Rapid Self-Healing by Multiple Hydrogen Bonds. Polymer.

[110] Li J., Du X., Zhang A., Wen J., Shuai L., Li S., Zhu M., Nie Y. (2024). Hydrogen-Bonded Polymeric Materials with High Mechanical Properties and High Self-Healing Capacity. Mater. Chem. Front..

[111] Karimi Alavijeh S., Khorasani S.N., Masoomi M., Neisiany R.E., Dinari M., Gomes D.R., Pei Y., Raffa P. (2024). Synthesis and Properties of a Shape Memory–Assisted Self-Healing Supramolecular Polyurethane Coating Based on Quadrupole Hydrogen Bonding. Polym. Adv. Technol..

[112] Song Y., Li J., Song G., Li X. (2024). Tough and Self-Healing Waterborne Polyurethane Elastomers via Dynamic Hydrogen Bonds Design for Flexible Conductive Substrate Applications. ACS Appl. Mater. Interfaces.

[113] Mauro M. (2018). Dynamic Metal–Ligand Bonds as Scaffolds for Autonomously Healing Multi-Responsive Materials. Eur. J. Inorg. Chem..

[114] Lai J.-C., Jia X.-Y., Wang D.-P., Deng Y.-B., Zheng P., Li C.-H., Zuo J.-L., Bao Z. (2019). Thermodynamically Stable Whilst Kinetically Labile Coordination Bonds Lead to Strong and Tough Self-Healing Polymers. Nat. Commun..

[115] Li J., Zhu X., Xu Z., Ren H., Muhammad S., Yang H., Luo B., Javed M.U., Yu C., Zhang Y. (2025). Dynamic Metal-Ligand Coordinated Self-Healing Polymer Electrolytes for Lithium-Ion Batteries: Correlating Coordination Mechanisms with Electrochemical Properties. Adv. Funct. Mater..

[116] Chen Y.-A., Lai R.-H., Lin W.-C., Huang H.-Y., Chen S.-J., Yeh C.-M., Huang H.-L., Elsenety M.M., Hu C.-C., Yu C.-H. (2024). Enhancing Self-Healing and Mechanical Robustness through Aluminum Acetylacetonate-Driven Metal–Ligand Coordination for Skin-Inspired Sensing. ACS Appl. Polym. Mater..

[117] Islam S., Bhat G. (2021). Progress and Challenges in Self-Healing Composite Materials. Mater. Adv..

[118] Miyagawa R., Shibata M. (2025). Self-Healing Thermoset Polyurethanes Driven by Host–Guest Interactions Between α-Cyclodextrin and Poly(Ethylene Glycol) Monomethyl Ether or Dodecanol Moieties. Molecules.

[119] Yi L., Zhao H., Yu J., Guo Q., Qiu H., Chen K. (2025). Design and Synthesis of Asymmetric *Comb*-like Waterborne Polyurethane for Self-Healing Chemical Protective Coatings. Chem. Eng. J..

[120] Xie P., Wu G., Zhen X., Wang G., Meng Y., Hu K., Liu Y., Pei X., Wu Y., Zhou F. (2025). Tailoring Microphase Separation via Host–Guest Stoichiometry in Supramolecular Polyurethanes for Ultratoughening and Self-Healing. ACS Appl. Polym. Mater..

[121] Yang Y., Ding X., Urban M.W. (2015). Chemical and Physical Aspects of Self-Healing Materials. Prog. Polym. Sci..

[122] Burattini S., Greenland B.W., Hayes W., Mackay M.E., Rowan S.J., Colquhoun H.M. (2011). A Supramolecular Polymer Based on Tweezer-Type Π−π Stacking Interactions: Molecular Design for Healability and Enhanced Toughness. Chem. Mater..

[123] Xiong H., Wu H., Zhang J., Huang S., Gu S., Hou Y., Wu Q., Wu J. (2023). Healable and Recyclable Polyurethane with Natural-Rubber-like Resilience via π-Type Tweezer Structure Stabilizing Dynamical Hard Domains. Macromolecules.

[124] Burattini S., Greenland B.W., Merino D.H., Weng W., Seppala J., Colquhoun H.M., Hayes W., Mackay M.E., Hamley I.W., Rowan S.J. (2010). A Healable Supramolecular Polymer Blend Based on Aromatic Π−π Stacking and Hydrogen-Bonding Interactions. J. Am. Chem. Soc..

[125] Trentin A., Uvida M.C., de Araújo Almeida A., de Souza T.A.C., Hammer P., Song H., Nguyen T.A., Yasin G., Singh N.B., Gupta R.K. (2022). Chapter 18—Self-Healing Nanocoatings. Nanotechnology in the Automotive Industry.

[126] Lai Y., Kuang X., Yang W.-H., Wang Y., Zhu P., Li J.-P., Dong X., Wang D.-J. (2021). Dynamic Bonds Mediate π-π Interaction via Phase Locking Effect for Enhanced Heat Resistant Thermoplastic Polyurethane. Chin. J. Polym. Sci..

[127] Yuan Z., Yan J., Gao F., Cheng J., Zhang J. (2023). High-Performance, Fluorescent, UV-Shielding, Triboelectric, Super-Flexible Polyurea Elastomers via Strong π–π Stacking of Pyrene and Hydrogen Bonding Strategies. J. Mater. Chem. C.

[128] Dai J., Wang Z., Wu Z., Fang Z., Heliu S., Yang W., Bai Y., Zhang X. (2023). Shape Memory Polymer Constructed by π–π Stacking with Ultrafast Photoresponse and Self-Healing Performance. ACS Appl. Polym. Mater..

[129] Peng Y., Zhao L., Yang C., Yang Y., Song C., Wu Q., Huang G., Wu J. (2018). Super Tough and Strong Self-Healing Elastomers Based on Polyampholytes. J. Mater. Chem. A.

[130] Joo H., Gwak S., Park H., Yoon H.-J., Ryu H., Han S.A., Lee J.-H. (2024). Engineering Self-Healable and Biodegradable Ionic Polyurethane with Highly Tribopositive Behavior. Nano Energy.

[131] Li H., Guo Y., Zheng C., Lin L., Wu H., Mao H. (2025). Ionic Interactions Reinforced Polyurethane with Self-Healing and Recyclable Properties for Composite Conductors. Eur. Polym. J..

[132] Yang Y., Urban M.W. (2013). Self-Healing Polymeric Materials. Chem. Soc. Rev..

[133] Ghanbari R., Serajian A., Ataei S., Nazarzadeh Zare E. (2024). Intrinsic Self-Healing Polyurethanes: Advances, Applications, and Future Prospects. Eur. Polym. J..

[134] Winne J.M., Leibler L., Du Prez F.E. (2019). Dynamic Covalent Chemistry in Polymer Networks: A Mechanistic Perspective. Polym. Chem..

[135] Wang S., Urban M.W. (2020). Self-Healing Polymers. Nat. Rev. Mater..

[136] Cao Q., Zhang L., Zhang F., Wang D., Wu D., Huang K. (2024). Self-Healing Polyurethane Based on a Substance Supplement and Dynamic Chemistry: Repair Mechanisms and Applications. J. Appl. Polym. Sci..

[137] Li Y., Zhou M., Wang R., Han H., Huang Z., Wang J. (2024). Self-Healing Polyurethane Elastomers: An Essential Review and Prospects for Future Research. Eur. Polym. J..

[138] Yu C., Kim H., Choi J., Song S., Choi J., Kwon M.S. (2026). Highly Tough, Notch-Insensitive, and Fast Self-Healing Thermoplastic Polyurethane Elastomers by Tailored Soft Segment Design. Angew. Chem. Int. Ed..

[139] Kojio K., Nozaki S., Takahara A., Yamasaki S. (2020). Influence of Chemical Structure of Hard Segments on Physical Properties of Polyurethane Elastomers: A Review. J. Polym. Res..

[140] Hao Y., Zhu G. (2025). The Latest Advances in Mechanically Robust Self-Healing Polyurea Based on Dynamic Chemistry. Adv. Sci..

[141] Zhang A., Li J., Fan H., Xiang J., Wang L., Yan J. (2022). Effect of Mechanical Properties on the Self-Healing Behavior of Waterborne Polyurethane Coatings. J. Appl. Polym. Sci..

[142] Wang Z., Lu X., Sun S., Yu C., Xia H. (2019). Preparation, Characterization and Properties of Intrinsic Self-Healing Elastomers. J. Mater. Chem. B.

[143] Roppolo I., Caprioli M., Pirri C.F., Magdassi S. (2024). 3D Printing of Self-Healing Materials. Adv. Mater..

[144] Tsangouri E., Aggelis D., Van Hemelrijck D. (2015). Quantifying Thermoset Polymers Healing Efficiency: A Systematic Review of Mechanical Testing. Prog. Polym. Sci..

[145] Feng L., Zhu D., Zhang H., Bu Q., Chen H., Guo C., Jiao K., Liu Y., Zhang J., Ye J. (2026). Degradable and Self-Repairable Polyurethanes with Superior Mechanical Properties and Remarkable Healing Capability. Polymer.

[146] Guo C., Chen H., Ye J., Ran J., Feng L., Liu Y., Zhang J., Li D. (2026). Resolving Mechanical Properties and Healing Performance Trade-off via TiO_2_/CuS Heterojunction Nanoparticles for Sunlight-Driven Self-Healing Polyurethane. Prog. Org. Coat..

[147] Ye J., Zhang J., Feng L., Liu H., Zhu D., Liu Y. (2024). Polyurethane Combining Preeminent Toughness, Puncture-Resistance, and Self-Healing Capabilities by Multiple Repair Options Based on Dual Dynamic Covalent Bonds. Colloids Surf. A Physicochem. Eng. Asp..

[148] Tian W., Wang S., Guo Z., Yu H., Tian L. (2023). Antifouling and Anticorrosion Function of Repeatable Self-Healing Polyurethane Composite Inspired by the Self-Healing Principle of Cartilage Tissue. Chem. Eng. J..

[149] Feng L., Yu Z., Bian Y., Wang Y., Zhao Y., Gou L. (2018). Effect of Failure Modes on Healing Behavior and Multiple Healing Capability of Self-Healing Polyurethanes. Constr. Build. Mater..

[150] Zhu D., Guo C., Liu H., Ye J., Chen H., Zhang J., Liu Y., Feng L. (2025). Thermo-Reversible DA Bonds-Based Polyurethanes with Both Excellent Comprehensive Mechanical Property and Remarkable Room Temperature Self-Healing Capability. Colloids Surf. A Physicochem. Eng. Asp..

[151] Wang Y., Deng Y., Wang K. (2026). Thermally Self-Healing Silver Nanowires–Diels–Alder Polyurethane Composite Films for Flexible Transparent Conductive Electrodes. ACS Appl. Electron. Mater..

[152] Liu X., Wu J., Wu J., Huang Z., Shen W., Yin X., Sun Y., Lin X., Lin W., Yi G. (2024). Tough, Self-Healing, and Recyclable Cross-Linked Polyurethane Elastomers via Synergistic Effect of Dual Dynamic Covalent Bonds. J. Appl. Polym. Sci..

[153] Huang L.-Q., Huang H.-X., Yu N., Chen C.-Z., Liu Y., Hu G.-H., Du J., Zhao H. (2025). High-Strength and Excellent Self-Healing Polyurethane Elastomer Based on Rigid Chain Segment Reinforcement. Macromolecules.

[154] Liu X., Huang Z., Wu J., Wu J., Luo H., Sun Y., Lin X., Lin W., Yi G. (2024). Photothermal-Responsive Lignin-Based Polyurethane with Mechanically Robust, Fast Self-Healing, Solid-State Plasticity and Shape-Memory Performance. Int. J. Biol. Macromol..

[155] (2024). Self-Healing and Reprocessable Biobased Non-Isocyanate Polyurethane Elastomer with Dual Dynamic Covalent Adaptive Network for Flexible Strain Sensor. Chem. Eng. J..

[156] Pan T., Yuan L., Liang G., Gu A. (2025). Mechanically Robust and Thermal Stable Polyurethane Elastomers with Self-Healing and Recycling Ability. Polym. Eng. Sci..

[157] Yang T., Lin C., Huang M., Ying P., Zhang P., Wu J., Wang T., Kovalev A., Myshkin N., Levchenko V. (2024). Self-Healing and Recyclable Polyurethane/Nanocellulose Elastomer Based on the Diels–Alder Reaction. Polymers.

[158] Mou X., Guo Y., Lai X., Ding J., Li H., Zeng X. (2025). Self-Healing and Reprocessable Polyurethane Elastomer with Triple Dynamic Crosslinked Networks. Macromol. Rapid Commun..

[159] Meng Q., Xu Z., Yu Y., Li Y., Diaby A.L., Araby S. (2026). Facile Synthesis of High-Performance and Self-Healing Polyurethane-Urea Nanocomposites Reinforced with Graphene. Nano Mater. Sci..

[160] Jiang C., Yin X., Liang S., Wang X., An H. (2026). Efficient NIR and Heat Co-Triggered Self-Healing MXene-Polyurethane Hybrid Coating With Superior Corrosion Resistance. Small.

[161] Zhu Y., Deng H., Luo H., Luo Y., Chen Y., Chen Z., Zhang C. (2025). Progress in the Development of Self-Healing Polyurethane Materials. Resour. Chem. Mater..

[162] Jung J., Lee S., Kim H., Lee W., Chong J., You I., Kang J. (2024). Self-Healing Electronic Skin with High Fracture Strength and Toughness. Nat. Commun..

[163] Huang J., Zhang L., Tang Z., Guo B. (2018). Bioinspired Engineering of Sacrificial Bonds into Rubber Networks towards High-Performance and Functional Elastomers. Compos. Commun..

[164] Zhou X., Guo B., Zhang L., Hu G.-H. (2017). Progress in Bio-Inspired Sacrificial Bonds in Artificial Polymeric Materials. Chem. Soc. Rev..

[165] Wen H.-T., Kuo C.-Y., Cheng Y.-W. (2025). Multifunctional Self-Healing Polyurethane Sensors Enabled by Disulfide-Hydrogen Bond Networks for Human Motion Monitoring. Eur. Polym. J..

[166] (2025). High-Performance Low-Temperature Self-Healing Bio-Based Polyurethane Triboelectric Nanogenerator for Wireless Intelligent Target Systems. Nano Energy.

[167] Li C.-H., Zuo J.-L. (2020). Self-Healing Polymers Based on Coordination Bonds. Adv. Mater..

[168] Liu J., Liu Y., Wang Y., Zhu J., Yu J., Hu Z. (2017). Disulfide Bonds and Metal-Ligand Co-Crosslinked Network with Improved Mechanical and Self-Healing Properties. Mater. Today Commun..

[169] Cai M.-Y., Su S., Du W.-T., Xu R.-J., Xiang H.-P. (2026). Self-Healing, High-Resilience, and UV-Curable Polyurethane-Urea Elastomers Enabled by Dual Crosslinked Networks. Chin. J. Polym. Sci..

[170] Cheng B.-X., Zhang J.-L., Jiang Y., Wang S., Zhao H. (2023). High Toughness, Multi-Dynamic Self-Healing Polyurethane for Outstanding Energy Harvesting and Sensing. ACS Appl. Mater. Interfaces.

[171] Yin C., Zhao Q., Li H., Liu L., Wang W., Li W. (2025). ZnS/Ti3C2Tx-Based Light-Triggered Self-Healing Coating Featuring Dual-Dynamic Diels-Alder and Disulfide Bonds for Sustainable Corrosion Protection. Chem. Eng. J..

[172] Tian W., Xiong F., Han H., Li W. (2025). Recyclable and Self-Healing Polyurethane Composite Film Based on Disulfide and DA Bond Modulation Strategies for Marine Anticorrosion Applications. Prog. Org. Coat..

[173] Mao J., Wang J., Yan L., Cao Y., Huang M., Chen W., Chen K., Liang Y., Chen W., Wang Y. (2025). Synergistic Dual Dynamic Covalent Networks Enable High-Performance and Self-Healing Polyurethane and Its Anticorrosion Applications. Macromolecules.

[174] Li M., He Z., Sun M., Yu L., Kong L. (2026). Preparation and Self-Healing Properties of Polyurethane with Dual Dynamic Covalent Bonds. Coatings.

[175] Li Y., Jin Y., Chen H., Zhou R., Mei J., Mao Z. (2025). A Visible Light-Responsive, Fast Room-Temperature Self- Healing, Mechanically Robust, Antibacterial Waterborne Polyurethane Based on Triple Dynamic Bonds. ACS Appl. Mater. Interfaces.

[176] Tahir N.A., Ahmad Zubir S., Shuib R.K. (2026). Designing Ambient Self-Healing, Anticorrosive, Transparent, and Resilient Hydroxyl Terminated Polybutadiene Based Polyurethane Coatings: Beyond One Dynamic Mechanism. J. Polym. Sci..

[177] Zhang W., Xuan H., Xu X., Lou J., Guan Q., You Z. (2025). Multiple Dynamic Bonds Enable High Mechanical Strength and Efficient Room-Temperature Self-Healable Polyurethane for Triboelectric Nanogenerators. Sci. China Chem..

[178] Huang R., Yang A., Ma R., Yang H., Tang X., Zhao F., He R., Zhang K., Yang W. (2025). Polyurethane Solid-Solid Phase Change Materials Based on Triple Dynamic Bonds with Excellent Mechanical and Self-Healing Properties for Sustainable Thermal Energy Storage. J. Energy Storage.

[179] Wang H., Xu D., Zhang Y., Yang W., Xu P., Niu D., Chen C., Ma P., Ma P. (2025). Biobased Polyurethane Materials with Self-Healing and Recycling Capabilities for Sustainable Applications in Triboelectric Devices. ACS Sustain. Chem. Eng..

[180] Pešić I., Durand J.-O., Radović D.V., Ostojić S., Petrović M., Radojević V., Spasenović M., Pergal M.V. (2025). Synthesis and Properties of in Situ Prepared Polyurethane/PEG-MXene Nanocomposites. Prog. Org. Coat..

[181] Zhang J., Zhu L., Li H., Cheng C., Jin S. (2026). Recent Progress of Morphology-Controllable Covalent Organic Frameworks: From Microscopic to Macroscopic Scale Synthesis and Their Multifunctional Applications. Nano Res..

[182] Xiao N., Chen Y., Lin H., liaquat H., Zhang F., Yang K. (2022). Multidimensional Nanoadditives in Tribology. Appl. Mater. Today.

[183] Gołąbek J., Sulewska N., Strankowski M. (2025). Self-Healing 3D-Printed Polyurethane Nanocomposites Based on Graphene. Micromachines.

[184] Yu Y., Xu Z., Xu L., Li Y., Liu T., Meng Q., Su X., Kuan H.-C., Dai J., Ma J. (2024). Highly Stretchable, Sensitive and Healable Polyurethane-Urea/Graphene Nanocomposite Sensor for Multifunctional Applications. Thin-Walled Struct..

[185] Niu H., Liu L., Zhu Y., Zhou C., Wu G. (2024). Liquid Metal-Enhanced Self-Healing Dual-Hard-Phase Cross-Linked Waterborne Polyurethane for Flexible Sensors. Eur. Polym. J..

[186] Yuan H., Ji C., Yuan H. (2023). Photothermal Self-Healing Polyurethane/Graphene Oxide Composites Based on Diselenide Bond. Polym. Test..

[187] Huang R., Zhao F., Li Y., Jiang Z., He F., Zhang K., Chen Z., He G., Yang W. (2025). GO-Enhanced Polyurethane Phase Change Materials with Triple Dynamic Bonds: Oxime-Urethane, Coordination and Hydrogen Bonds. Chem. Eng. J..

[188] Tian W., Xiong F., Han H., Li W. (2025). A Polyurethane/Functionalized Reduced Graphene Oxide Coating Based on a Hybrid Cross-Linking Strategy for Marine Corrosion Protection. Prog. Org. Coat..

[189] Cui Y.-Y., Zhang M.-Y., Gong L.-J., Liu C.-S., Han Y.-L., Li H.-J., Liu Y., Pan H.-W., Wu Y.-C. (2026). Photothermal-Activated Self-Healing Polyurethane for Comprehensive Marine Protection Integrating Antifouling and Anticorrosion Functions. Adv. Funct. Mater..

[190] Baughman R.H., Zakhidov A.A., de Heer W.A. (2002). Carbon Nanotubes—The Route Toward Applications. Science.

[191] De Volder M.F.L., Tawfick S.H., Baughman R.H., Hart A.J. (2013). Carbon Nanotubes: Present and Future Commercial Applications. Science.

[192] Kausar A. (2021). Self-Healing Polymer/Carbon Nanotube Nanocomposite: A Review. J. Plast. Film. Sheeting.

[193] Kong H., Luo X., Zhang P., Feng J., Li P., Hu W., Wang X., Liu X. (2023). Self-Healing, Solvent-Free, Anti-Corrosion Coating Based on Skin-like Polyurethane/Carbon Nanotubes Composites with Real-Time Damage Monitoring. Nanomaterials.

[194] Dong F., Yang X., Guo L., Qian Y., Sun P., Huang Z., Xu X., Liu H. (2023). A Tough, Healable, and Recyclable Conductive Polyurethane/Carbon Nanotube Composite. J. Colloid. Interface Sci..

[195] Zeng Y., Long L., Li Y., Li Y., Zhou W. (2025). Noncovalent Modification of Self-Healing Carbon Nanotubes/Polyurethane Composites for High-Efficiency Electromagnetic Wave Absorption and Multifunctionality. J. Alloys Compd..

[196] Alhabeb M., Maleski K., Anasori B., Lelyukh P., Clark L., Sin S., Gogotsi Y. (2017). Guidelines for Synthesis and Processing of Two-Dimensional Titanium Carbide (Ti3C2Tx MXene). Chem. Mater..

[197] Anasori B., Naguib M., Guest Editors (2023). Two-Dimensional MXenes. MRS Bull..

[198] Zhan X., Si C., Zhou J., Sun Z. (2020). MXene and MXene-Based Composites: Synthesis, Properties and Environment-Related Applications. Nanoscale Horiz..

[199] Yücer S., Sarac B., Adıgüzel B., Ciftci F. (2026). Wearable Electronic Patch for Physicochemical Data Transmission: MXene-Based MEMS/NEMS Biosensors. Mater. Today Phys..

[200] MXene-Infused Bioelectronic Interfaces for Multiscale Electrophysiology and Stimulation|Science Translational Medicine. https://www.science.org/doi/10.1126/scitranslmed.abf8629.

[201] Samejo B.A., Naseer K., Samejo S., Janjhi F.A., Memon N., Castro-Muñoz R., Boczkaj G. (2024). MXene-Based Composites for Capacitive Deionization—The Advantages, Progress, and Their Role in Desalination—A Review. Water Resour. Ind..

[202] Zhu Z., Huang J., Zhao Y., Hu Z., Chen Y., Guo L., Ding M. (2026). Review on the Rise of MXenes in Ion-Selective Technologies: A Decade of Progress and Future Directions. Desalination.

[203] Gürbüz B., Ciftci F. (2024). Bio-Electric-Electronics and Tissue Engineering Applications of MXenes Wearable Materials: A Review. Chem. Eng. J..

[204] Lin G., Guo T., Lin W., Fan H., Guo L., Zhang Z., Li B., Wang J., Ji H., Song W. (2025). Machine Learning Accelerated Screening Advanced Single-Atom Anchored MXenes Electrocatalyst for Nitrogen Fixation. ACS Catal..

[205] Naguib M., Barsoum M.W., Gogotsi Y. (2021). Ten Years of Progress in the Synthesis and Development of MXenes. Adv. Mater..

[206] Sui X., Yao W., Chen J., Han S., Yang D., Li J., Wang K., Wu W., Pei H., Tian Q. (2025). Light-Driven, Super-Fast Self-Healing Transparent MXene/W18O49/Polyurethane Films with Superior Toughness for Thermal Management. Adv. Sci..

[207] Li Z., Song Y., Wang X., Li J., Yang X., Liu H., Song G., Shen Z., Li Q., Chen Z. (2025). Mechanically Robust, Intrinsic Self-Healing Polyurethane Enabled by NIR-Photothermal Conversion of MXene/Polydopamine Hybrids. ACS Appl. Mater. Interfaces.

[208] Wen X., Deng Z., Wang H., Shi J., Wang S., Wang H., Song Y., Du Z., Qiu J., Cheng X. (2023). High Strength, Self-Healing Sensitive Ionogel Sensor Based on MXene/Ionic Liquid Synergistic Conductive Network for Human-Motion Detection. J. Mater. Chem. B.

[209] Yan K., Chen H., Li X., Xu F., Wang J., Xu Q., Zong Y., Zhang Y. (2024). Scalable and Multifunctional Polyurethane/MXene/Carbon Nanotube-Based Fabric Sensor toward Baby Healthcare. ACS Appl. Mater. Interfaces.

[210] Howarth A.J., Peters A.W., Vermeulen N.A., Wang T.C., Hupp J.T., Farha O.K. (2017). Best Practices for the Synthesis, Activation, and Characterization of Metal–Organic Frameworks. Chem. Mater..

[211] Li D., Yadav A., Zhou H., Roy K., Thanasekaran P., Lee C. (2024). Advances and Applications of Metal-Organic Frameworks (MOFs) in Emerging Technologies: A Comprehensive Review. Glob. Chall..

[212] Yusuf V.F., Malek N.I., Kailasa S.K. (2024). Correction to “Review on Metal–Organic Framework Classification, Synthetic Approaches, and Influencing Factors: Applications in Energy, Drug Delivery, and Wastewater Treatment.”. ACS Omega.

[213] Liu P., Gao S., Wang Y., Zhou F., Huang Y., Luo J. (2020). Metal-Organic Polymer Coordination Materials Derived Co/N-Doped Porous Carbon Composites for Frequency-Selective Microwave Absorption. Compos. Part B Eng..

[214] Zhang J., Singh V., Huang W., Mandal P., Tiwari M.K. (2023). Self-Healing, Robust, Liquid-Repellent Coatings Exploiting the Donor–Acceptor Self-Assembly. ACS Appl. Mater. Interfaces.

[215] Wang X., Xu J., Zhang Y., Wang T., Wang Q., Li S., Yang Z., Zhang X. (2023). A Stretchable, Mechanically Robust Polymer Exhibiting Shape-Memory-Assisted Self-Healing and Clustering-Triggered Emission. Nat. Commun..

[216] Liu F., Ye P., Cheng Q., Zhang D., Nie Y., Shen X., Zhu M., Xu H., Li S. (2024). By Introducing Multiple Hydrogen Bonds Endows MOF Electrodes with an Enhanced Structural Stability. Inorg. Chem..

[217] Li W., Xie C., Zhang P., Zhang Z., Xue M., Yin Z., Luo Y., Hong Z. (2025). Constructing Dual-Ligand Ce-MOF on Graphene Oxide Modified with Polydopamine Endowing Polyurethane Coating with Long-Term Smart Anti-Corrosion and Mechanical Robustness. J. Colloid Interface Sci..

[218] Li C., Song Y., Chen D., Li G.L. (2026). Room-Temperature Self-Healing Composites with Excellent Strength and Toughness for Anticorrosion Applications. ACS Appl. Mater. Interfaces.

[219] Li H.-W., Zhang J.-L., Xue R., An Z.-W., Wu W., Liu Y., Hu G.-H., Zhao H. (2023). Construction of Self-Healable and Recyclable Waterborne Polyurethane-MOF Membrane for Adsorption of Dye Wastewater Based on Solvent Etching Deposition Method. Sep. Purif. Technol..

[220] Ge S., Wei K., Peng W., Huang R., Akinlabi E., Xia H., Shahzad M.W., Zhang X., Xu B.B., Jiang J. (2024). A Comprehensive Review of Covalent Organic Frameworks (COFs) and Their Derivatives in Environmental Pollution Control. Chem. Soc. Rev..

[221] Jiang Y., Ren J., Yuan Y., Keshta B.E., Zhang Y., Guan X., He Y., Zhao D., Chen B. (2026). Covalent Organic Frameworks for Gas Storage and Separation. Chem. Soc. Rev..

[222] Chen Y., Jiang D. (2024). Photocatalysis with Covalent Organic Frameworks. Acc. Chem. Res..

[223] Asif M.B., Kim S., Nguyen T.S., Mahmood J., Yavuz C.T. (2024). Covalent Organic Framework Membranes and Water Treatment. J. Am. Chem. Soc..

[224] Zhu L., Cao Y., Xu T., Yang H., Wang L., Dai L., Pan F., Chen C., Si C. (2025). Covalent Organic Framework Membranes for Energy Storage and Conversion. Energy Environ. Sci..

[225] Singh P., Dave P.N. (2025). Covalent Organic Frameworks (COFs) as Advanced Carriers for Corrosion Prevention: Cutting-Edge Advances and Promising Future Opportunities. ChemNanoMat.

[226] Chen G., Lou Y., Li J., Chen L., Xing Z., Zhang T., Gu D., Peng Y., Wu H. (2024). Transparent, Self-Healing, and Defogging Wearable Devices Enabled by Plasmonic Silver Nanoparticle-Embedded Covalent–Organic Framework Nanosheets. ACS Mater. Lett..

[227] Zhang Y., Dong H., Ma Z., Yang W., Zhang H., Wei C., Zhu S., Lu H., Xuan S., Yang W. (2025). Near-Infrared-Triggered Self-Healing and Antibacterial Biobased Waterborne Polyurethane Enabled by Porphyrin Covalent Organic Frameworks. ACS Appl. Polym. Mater..

[228] Xu J., Wang X., Zhang X., Zhang Y., Yang Z., Li S., Tao L., Wang Q., Wang T. (2023). Room-Temperature Self-Healing Supramolecular Polyurethanes Based on the Synergistic Strengthening of Biomimetic Hierarchical Hydrogen-Bonding Interactions and Coordination Bonds. Chem. Eng. J..

[229] Duan X., Cao W., He X., Wang M., Cong R., Zhang Z., Ning C., Wang C., Zhao S., Li Z. (2023). Realization of Dual Crosslinked Network Robust, High Toughness Self-Healing Polyurethane Elastomers for Electronics Applications. Chem. Eng. J..

[230] Wang X., Wang L., Liu C., Cao Y., He P., Cui Y., Li H. (2024). Self-Healing Polyurethane Elastomers with Superior Tensile Strength and Elastic Recovery Based on Dynamic Oxime-Carbamate and Hydrogen Bond Interactions. Macromol. Rapid Commun..

[231] Wang X., Li S., Yang Z., Zhang Y., Wang Q., Wang T., Zhang X. (2025). Multifunctional Polyurethane Exhibiting High Mechanical Performance and Shape-Memory-Assisted Self-Healing. Small.

[232] Kim S., Jeon H., Koo J.M., Oh D.X., Park J. (2024). Practical Applications of Self-Healing Polymers Beyond Mechanical and Electrical Recovery. Adv. Sci..

[233] Du J., Zhao X., Li Y., Lei W., Zhou X. (2025). Development of Self-Healing Polyurethane and Applications in Flexible Electronic Devices: A Review. Polymers.

[234] Xie H., Liu X., Sheng D., Wu H., Zhou Y., Tian X., Sun Y., Shi B., Yang Y. (2021). Novel Titin-Inspired High-Performance Polyurethanes with Self-Healing and Recyclable Capacities Based on Dual Dynamic Network. Polymer.

[235] Devi R., Gupta P., Khatua C., Naskar K., Chattopadhyay S. (2025). Thermoplastic Polyurethane-Based Stimuli-Responsive Nanocomposites: A Review on Self-Healing and Shape Memory Properties. Polym. Eng. Sci..

[236] Integrating Photothermal-Responsive Shape Memory and Self-Healing Polymers in 4D-Printed Thermally Comfortable Smart Wearables | ACS Applied Engineering Materials|ACS Publications. https://pubs.acs.org/aaemdr/article-abstract/2/11/2569/347725/Integrating-Photothermal-Responsive-Shape-Memory?redirectedFrom=fulltext.

[237] Zheng S., Zhou K., Zhang X., Ren N. (2025). Metal–Organic Framework/MXene-Based Materials: Preparations and Applications. RSC Adv..

[238] Stoll J.L., Paul M., Pritchett L., Snover A., Woods L., Antony Jose S., Menezes P.L. (2025). Tribology of MXene Materials: Advances, Challenges, and Future Directions. Materials.

[239] Zhou Y., Zhang M., Hu H., Bi C., Zhang H., Ye Y., Yan C. (2026). Research Progress of Polymer-Based Coatings for Anti-Corrosion Application: A Mini Review. J. Polym. Sci..

[240] Yang H., Yang Y., Li Y., Hope J., Choo W. (2023). Extrinsic Conditions for the Occurrence and Characterizations of Self-Healing Polyurea Coatings for Improved Medical Device Reliability: A Mini Review. ACS Omega.

[241] Luo J., Wang T., Sim C., Li Y. (2022). Mini-Review of Self-Healing Mechanism and Formulation Optimization of Polyurea Coating. Polymers.

[242] Antony Jose S., Arvisu E., Menezes P.L. (2026). Self-Healing Materials: Mechanisms, Properties, and Applications. Processes.

[243] Priyadharshini R., Xavier J. (2026). Recent Progress in Environmentally Friendly Coatings with Improved Corrosion Resistance, Mechanical Durability, and Antibiofouling Properties. Polym. Bull..

[244] Xing K., Li H., Wang X. (2026). Durability Enhancement of Prestressed Concrete Using Smart Materials: Integrating Self-Healing Mechanisms and Monitoring Systems. Sustain. Resilient Infrastruct..

[245] Zaed M.A., Tan K.H., Abdullah N., Saidur R., Pandey A.K., Saleque A.M. (2024). Cost Analysis of MXene for Low-Cost Production, and Pinpointing of Its Economic Footprint. Open Ceram..

[246] Yousef S. (2025). Techno-Economic Assessment of Scale-up of Metal-Organic Framework Production. J. Indian Chem. Soc..

[247] Vekemans V., Parisi S., Wu J., Karana E. (2026). Societal Implications of Self-Healing Engineered Living Materials: Insights from a Co-Design Study. Biotechnol. Des..

[248] Feng H., Wang Y., Zhang T., Wang J., Jia Z., Jiang S., Han X. (2025). Review on Research Progress on Bio-Based Self-Healing Polyurethane. ACS Mater. Lett..

[249] Wang X., Zhang X., Li W., Wang W., Zhu J., Chen S. (2025). Preparation and Performance of High-Efficiency Photothermal Multifunctional Self-Healing Polyurethane Coating Based on MoS2@CuS@ZIS Ternary Heterojunction and Double Dynamic Covalent Bond. Compos. Part A Appl. Sci. Manuf..

